# Electronic structure of metal oxide dications with ammonia ligands and their reactivity towards the selective conversion of methane to methanol

**DOI:** 10.3389/fchem.2024.1508515

**Published:** 2024-12-11

**Authors:** Emily E. Claveau, Evangelos Miliordos

**Affiliations:** Department of Chemistry and Biochemistry, Auburn University, Auburn, AL, United States

**Keywords:** multireference approach, catalysis, methane-to-methanol, hydrogen bonding, ammonia ligand, first-row transition metal complexes

## Abstract

High-level quantum chemical calculations are performed for the (NH_3_)MO^2+^ and (NH_3_)_5_MO^2+^ species (M = Ti-Cu), extending our previous work on the bare MO^2+^ ions. The potential energy curves along the M-O distance are constructed for the ground and multiple excited electronic states of (NH_3_)MO^2+^ and are compared to those of MO^2+^. We see that ammonia stabilizes the oxo states (M^4+^O^2−^) over the oxyl (M^3+^O^⋅−^) ones. This trend is intensified in the (NH_3_)_5_MO^2+^ species. We then examined the reaction of the latter species with both methane and methanol. We find that the oxyl states activate a C-H bond easily with barriers smaller than 10 kcal/mol across all first-row transition metals, while the barriers for the oxo states start from about 50 kcal/mol for M = Ti and decrease linearly to 10 kcal/mol going toward M = Ni. This is attributed to the increasing spin density on the oxygen atom observed for the oxo states. The most important finding is that the formation of hydrogen bonds between the OH group of methanol and the N-H bonds of the ammonia ligands increases the activation barriers for methanol considerably, making them comparable to and slightly higher than those of methane. This finding suggests a new strategy to slow the oxidation of methanol, leading to the long-desired higher methane-to-methanol selectivity.

## Graphical Abstract

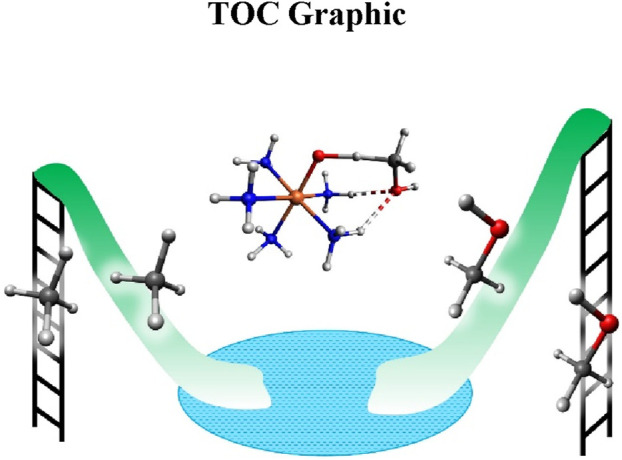

## 1 Introduction 

Transition metal oxides have been widely studied in all forms, from bare diatomic species to molecular complexes and crystalline forms (heterogeneous catalysts). Bare (early) transition metal mono-oxides have been found in the observable regions of high-temperature outer planetary atmospheres and have been used to classify M supergiants (White and Wing, 1978; Merer, 1989; Sedaghati et al., 2017), while metal oxide molecular complexes and heterogeneous catalysts are promising candidates for a variety of industrial applications. The present computational work focuses on the role of molecular metal oxides in the purer conversion of methane to methanol, an important chemical transformation for cleaner energy solutions.

Earlier studies have investigated their electronic structure and reactivity with multireference, density functional theory (DFT), and machine learning methods (Periana et al., 1998; Gaggioli et al., 2019; Nandy et al., 2022). Molecular transition metal oxides seem promising as potential catalysts for this process if certain issues can be overcome. One significant problem presented by Ravi et al. (2017) and Latimer et al. (2018) is that methanol continues to react, making formaldehyde and other overoxidized products. Therefore, both a method and a suitable catalyst need to be found to allow for efficient reactivity with a mechanism that prevents the oxidation of methanol. This presents a potential problem because the C-H bond of methanol is weaker than methane, so a proper catalyst and reaction are necessary to carry out the CH bond activation of methane without activating the methanol bonds (Gaggioli et al., 2019). No industrially viable solution has been identified to date, and only a few studies focus on the activation of methanol by metal oxides.

Transition metal oxide dications may be suitable catalysts for these processes due to their unique ability to switch between two forms: oxo (M^4+^O^2−^) and oxyl (M^3+^O^⋅−^). (Claveau et al., 2023b). Nature chooses dications for several natural and biological processes, such as the heme iron complexes found in blood and in various enzymes (Hohenberger et al., 2012; Poulos, 2014; Ray et al., 2014; Martinie et al., 2017; Mitchell et al., 2017; Follmer et al., 2019). These dications have also important industrial applications (Hohenberger et al., 2012; Ray et al., 2014). The oxo form is characterized by a closed shell oxygen side, and the oxyl form has a significant electronic spin density on oxygen (Shimoyama and Kojima, 2019). Each form follows different reaction mechanisms. The oxo form will follow either the 2 + 2 mechanism or the proton-coupled electron transfer (PCET) mechanism.(Khan and Miliordos, 2021). Both of these mechanisms can be selective but usually possess high energy barriers that the reaction must overcome. The oxyl form utilizes the radical mechanism, otherwise known as hydrogen atom transfer (HAT) (Gaggioli et al., 2019). The HAT mechanism is not selective but is characterized by having low energy barriers.

Our group has done extensive analysis on transition metal oxide cations, neutral forms, and anions to understand their electronic structure and potential reactivity (Almeida et al., 2018; Ariyarathna and Miliordos, 2018; Claveau and Miliordos, 2019; Khan and Miliordos, 2019; Liu et al., 2019; Ariyarathna et al., 2020; Ariyarathna and Miliordos, 2020; Jackson and Miliordos, 2020; Ariyarathna and Miliordos, 2021; Sader and Miliordos, 2021; Ariyarathna and Miliordos, 2022; Sader and Miliordos, 2022). Our computational work showed that anionic metal centers interact weakly with the produced methanol, which allows for its quick removal from the catalytic cycle to avoid overoxidation. Investigations into transition metal oxide dications have been ongoing for both first- and second-row transition metal oxides to categorize metals as oxo or oxyl based on their electronic structure as well as to explore their potential catalytic activity for methane activation (Kirkland et al., 2018; Almeida et al., 2019; Claveau and Miliordos, 2021; Khan and Miliordos, 2021; Claveau et al., 2023a).

The effect of different ligands on transition metal oxide dications to stabilize the oxo or oxyl character has begun to be investigated in the literature (Yang et al., 2014; Kirkland et al., 2018). A study of FeO^2+^ by Kirkland et al. (2018) showed that a strong field ligand, such as ammonia, can stabilize a higher energy oxo state to the ground state of the system (Kirkland et al., 2018). Yang et al. discuss a study of multiple (H_2_O)_6_MO^n+^ complexes to resemble hematite, and of the dications mentioned (V-Cu), all had a higher spin ground state as a result of ligand attachment, indicating a possible correlation between ligand effects and metal oxide stabilization (Yang et al., 2014). It is expected that the 2 + 2 and PCET mechanisms will be favored with a strong field ligand such as ammonia, similar to what is shown in the FeO^2+^ study. On the other hand, the oxyl form and HAT mechanisms are preferred with electron-withdrawing ligands, such as halogens, as was demonstrated for ZrO (Jackson and Miliordos, 2020).

Many DFT studies have been done concerning the quantification of the oxophilicity, the ability to form a metal–oxygen bond, for transition metal oxides (Kepp, 2016; Moltved and Kepp, 2019; Deraet et al., 2021; Kayode and Montemore, 2021). Work has also been done at the DFT level using machine learning methods to try and characterize proper functionals to give a reliable description of the ground state for neutral transition metal oxides (Liu et al., 2020; Østrøm et al., 2022). Several multireference studies have been carried out on neutral and singly charged transition metal oxides; however, few studies exist for the dication systems, and they refer to bare (i.e., no ligand) systems (Blomberg and Siegbahn, 1992; Nakao et al., 2001; Miliordos and Mavridis, 2010; Yang et al., 2014; Jiang et al., 2021).

Presently, our goal is to examine the electronic structure and reactivity of ligated MO^2+^ units, where M is a first-row transition metal. We are inspired by the work of Que and co-workers (Rasheed et al., 2018), who synthesized a series of FeO^2+^ complexes (N_5_FeO^2+^) with various ligands binding to the metal via tertiary (NR_3_, R = organic chain) or pyridinic nitrogen atoms (always forming a coordinative bond with their lone pair) and studied their reactivity toward methane activation. Similar complexes have been made for Ni, Mn, and Mo (Karunadasa et al., 2010; Leto et al., 2016; Bok et al., 2017). To simplify our calculations, we assess the replacement of the experimentally used ligands with simple ammonia for FeO^2+^ in terms of electronic structure and activation barriers for their reaction with methane, and we find minimal differences. We then study the reaction of these (NH_3_)_5_MO^2+^ complexes with methanol (such studies are rare in the literature), and we find that the hydrogen bonding between the ammonia ligands and the incoming methanol (missing in the experimentally synthesized complexes) can make the difference. The complexes increase the activation barriers for methanol by ∼20 kcal/mol or more and thus kinetically favor the activation of methane, suggesting higher overall selectivity for methanol production (smaller amounts of overoxidized products, such as formaldehyde), as happens for (NH_3_)_4_RhO^2+^ (Claveau et al., 2023a). Ammonia ligands should be replaced by more applicable ligands for real-life applications, perhaps functionalizing existing ligands and adding OH or NH_2_ groups, which will be the topic of future articles.

In Section 2, we discuss the computational approaches employed in this study. Section 3 discusses our results starting from mono-ligated (NH_3_)MO^2+^, which are compared with the bare MO^2+^ species we recently studied (Claveau and Miliordos, 2021). The interest then switches to the (NH_3_)_5_MO^2+^ complexes, and the FeO^2+^ complexes are compared to the experimental N_5_FeO^2+^ species. Section 4 summarizes our conclusions.

## 2 Methods

We initially optimized the geometry of the ground state for each (NH_3_)MO^2+^ at the DFT/MN15 (Khan and Miliordos, 2019) level of theory with the cc-pVTZ (N,H,M)/aug-cc-pVTZ(O) (Dunning, 1989; Kendall et al., 1992; Woon and Dunning, 1994; Balabanov and Peterson, 2005) basis sets. The MN15 functional was used for this process, as it is designed for characterizing transition metals, and we have seen excellent consistency between MN15 and CCSD(T) results in the past regarding energetics for similar transition metal systems [see, for example, Claveau et al. (2023a) and Khan and Miliordos, 2019)]. The aug-cc-pVTZ basis set is used on the oxygen to account for the polarized character of the metal–oxygen bond. Using this geometry, the potential energy curves as a function of the metal–oxygen distance are constructed via the complete active space self-consistent field (CASSCF) and multireference configuration interaction (MRCI) techniques. The active space of the reference CASSCF calculation consists of the three 2p orbitals of oxygen and the five 3d and one 4s orbitals of the metal, resulting in a nine-orbital active space. Electronic configurations were gathered at distances of interest (minima, shoulders) for the ground and several low-lying excited states. All valence electrons were correlated with MRCI.

Calculations for the (NH_3_)_5_MO^2+^ coordination complexes were carried out using Gaussian 16 software and the MN15 functional to first optimize the geometries, ensuring only real frequencies were returned from harmonic frequency calculations. The same triple-ζ basis was used here, and natural orbitals are analyzed at the same level of theory to describe the chemical bonding in the complexes. For the N_5_FeO^2+^ species, we used cc-pVDZ (N,H,M)/aug-cc-pVDZ(O) (Dunning, 1989; Kendall et al., 1992; Woon and Dunning, 1994; Balabanov and Peterson, 2005).

To investigate the reaction of these clusters with methane and methanol, the relative encounter complexes [(NH_3_)_5_MO^2+^(CH_4_)] and transition states were also obtained for the lowest state of each spin multiplicity. The latter returned only one imaginary frequency from harmonic frequency calculations. These optimizations were completed using the same MN15/triple-ζ methodology.

All DFT and multireference calculations were completed using the Gaussian 16 and MOLPRO 2021 software packages, respectively (Werner et al., 2015; Frisch et al., 2016). All optimized geometries are given in Supplementary Tables S1–S9, S12, S13.

## 3 Results and discussion

### 3.1 (NH_3_)MO^2+^ species

Our earlier work on MO^2+^ revealed that the plain mono-oxides can be divided into three groups: early (M = Ti-Cr), middle (M = Mn, Fe), and late (M = Co-Cu) transition metals (Claveau and Miliordos, 2021). This separation is based on the electronic structure of the low-lying electronic states. The first group has well-defined M^4+^O^2−^ oxo ground states that are well separated from the M^3+^O^•−^ oxyl excited states. Six of the valence electrons occupy the σ_MO_ and π_MO_ bonding orbitals (σ_MO_^2^π_MO_^4^), which are polarized toward oxygen, justifying an O^2−^ terminus. The remaining valence electrons populate the two δ_M_ orbitals, which are pure *3d* orbitals of the metal (δ_M_^0,1,2^ for Ti, V, and Cr, respectively), making the *X* ^1^Σ^+^, *X* ^2^Δ, and *X* ^3^Σ^−^/^1^Σ/^+1^Γ electronic states of TiO^2+^, VΟ^2+^, and CrO^2+^. The excited oxyl states are generated via a π_MO_ → π_MO_* (bonding → antibonding) promotion, producing the ^1,3^Φ, ^2^Π, and ^5^Σ^−^ states of the three metal oxides. The π_MO_* orbitals have a dominant metallic *3d* character. The emerged π_MO_^1^ electron creates the radical oxygen center. The middle metal oxides have ground states with long metal–oxygen bonds r_MO_ pointing to oxyl states (r_MO_ > 2.0 Å, ^4,6,8^Σ^−^ states for MnO^2+^ and ^3,5,7^Δ for FeO^2+^) but low-lying excited states of oxo character (r_MO_ ∼ 1.6 Å, ^4^Π for MnO^2+^ and ^5^Σ^+^ for FeO^2+^). In the oxo states, the additional electrons (compared to Cr) populate the π_MO_* instead of the half-filled δ_M_ orbitals. Finally, the late transition metal oxides have all long metal–oxygen bonds (r_MO_ > 2.0 Å) with no low-lying oxo states. It should be clarified that the π_MO_ orbitals change character, moving from Ti to Cu, shifting from highly localized on oxygen to highly localized on the metal, and the opposite happens for π_MO_*. This implies that the oxyl character should be assigned to a (π_MO_*)^1^ configuration for the late transition metals (rather than π_MO_^1^). For the middle metals, both π_MO_ and π_MO_* have substantial contributions from both metallic and oxygen orbitals.

**FIGURE 1:**
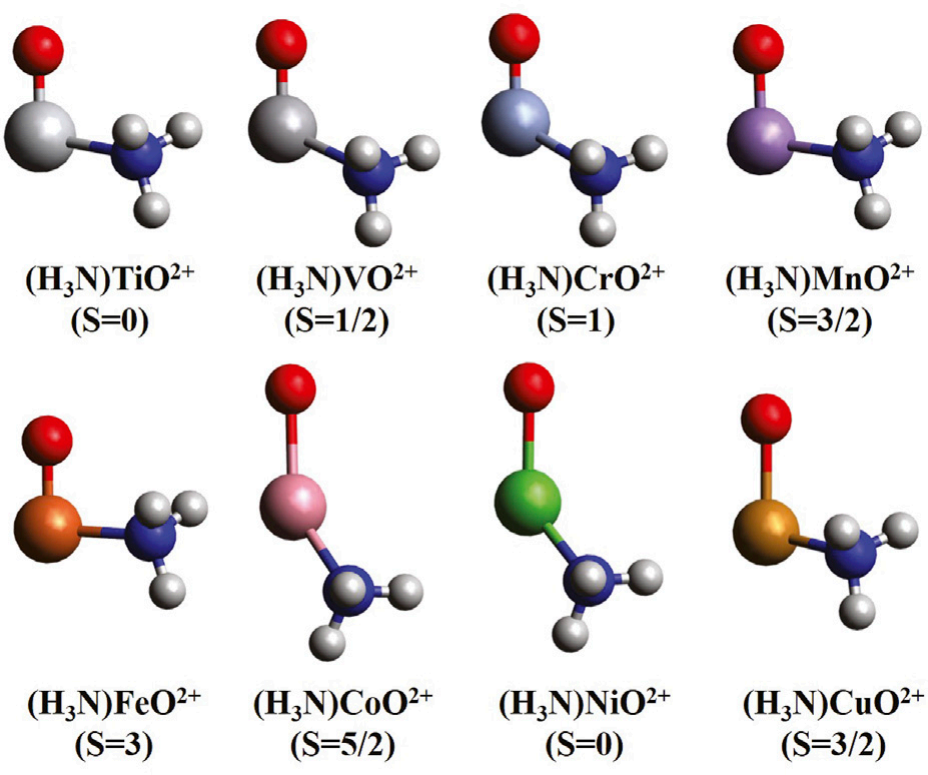
MN15 optimized equilibrium geometries of mono-ammonia ligated first-row transition metal oxide dications for the ground state of each complex.

The optimized geometries for the (NH_3_)MO^2+^ ground states are shown in Figure 1. The ammonia ligand binds at an angle with respect to the MO bond, which varies from 93.9^o^ (M = Fe) to 142^o^ (M = Co). This NMO angle is not 180^o^ as expected by conventional valence shell electron pair repulsion (VSEPR) arguments, even for M = Ti, which has no valence electrons on the metal. Although we cannot provide any explanation, it is related to the known trans-influence effect, according to which “ligands selectively weaken the bond trans to it” (Coe and Glenwright, 2000). The MO bond lengths are between 1.515 Å and 1.626 Å for Ti through Fe and between 2.001 Å and 2.079 Å for M = Co, Ni, and Cu. Compared to MO^2+^, the bond lengths for early and late transition metals remain nearly unaffected when ammonia coordinates, unlike the middle metals where the bond distances contract from values larger than 2.0 Å to 1.626 Å (M = Mn) and 1.558 Å (M = Fe). This indicates that the oxo states are stabilized over the oxyl ones for these two metals, which is confirmed by our MRCI calculations and discussed further below. The C_s_ symmetry is imposed by rotating the ammonia ligand by no more than 20° for the subsequent CASSCF calculations.

**FIGURE 2:**
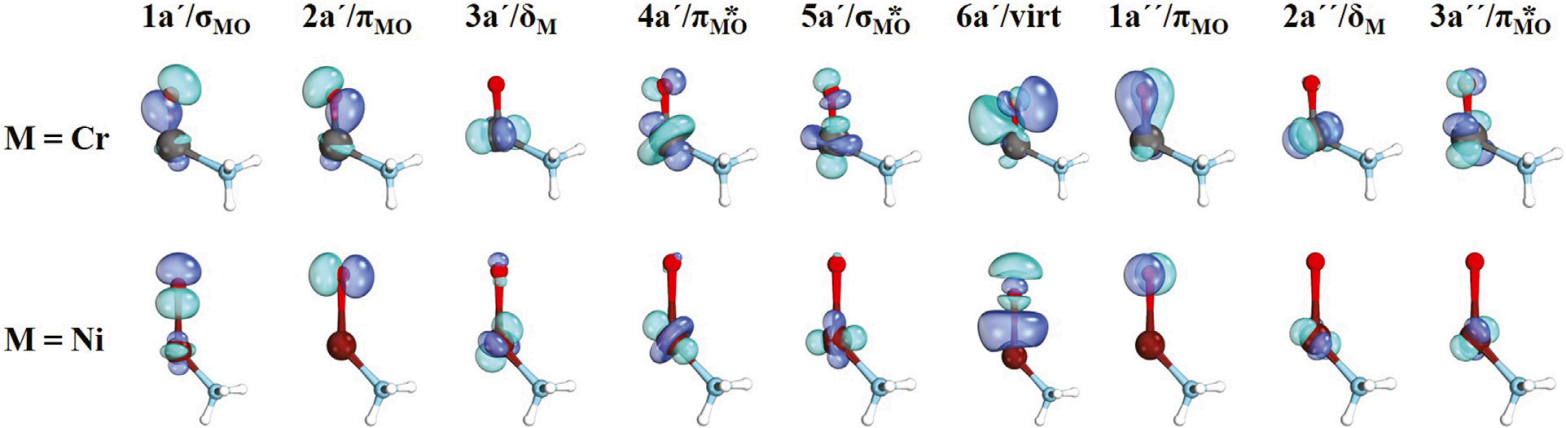
CASSCF active orbitals for (NH_3_)MO^2+^, M = Cr, Ni.

The CASSCF active orbitals for the (NH_3_)CrO^2+^ and (NH_3_)NiO^2+^ species are shown in Figure 2. Due to the lower symmetry, the σ and one of the π orbitals of the MO bond belong to the same (a΄) irreducible representation, and in principle, they can be mixed, which is observed for the case of σ_MO_/π_MO_ or 1a′/2a′ orbitals of (NH_3_)CrO^2+^ but not for (NH_3_)NiO^2+^. Generally, we were able to assign the a′ and a″ orbitals as σ_MO_, π_MO_, σ_MO_*, π_MO_*, and δ_M_. The 6a′ orbital is minimally occupied in all cases and has a 3p character of oxygen (denoted as virt in Figure 2). This was included to account for the 4s orbital of the metal, which is not populated because the metal center is of either M^4+^ or M^3+^ nature.

**FIGURE 3:**
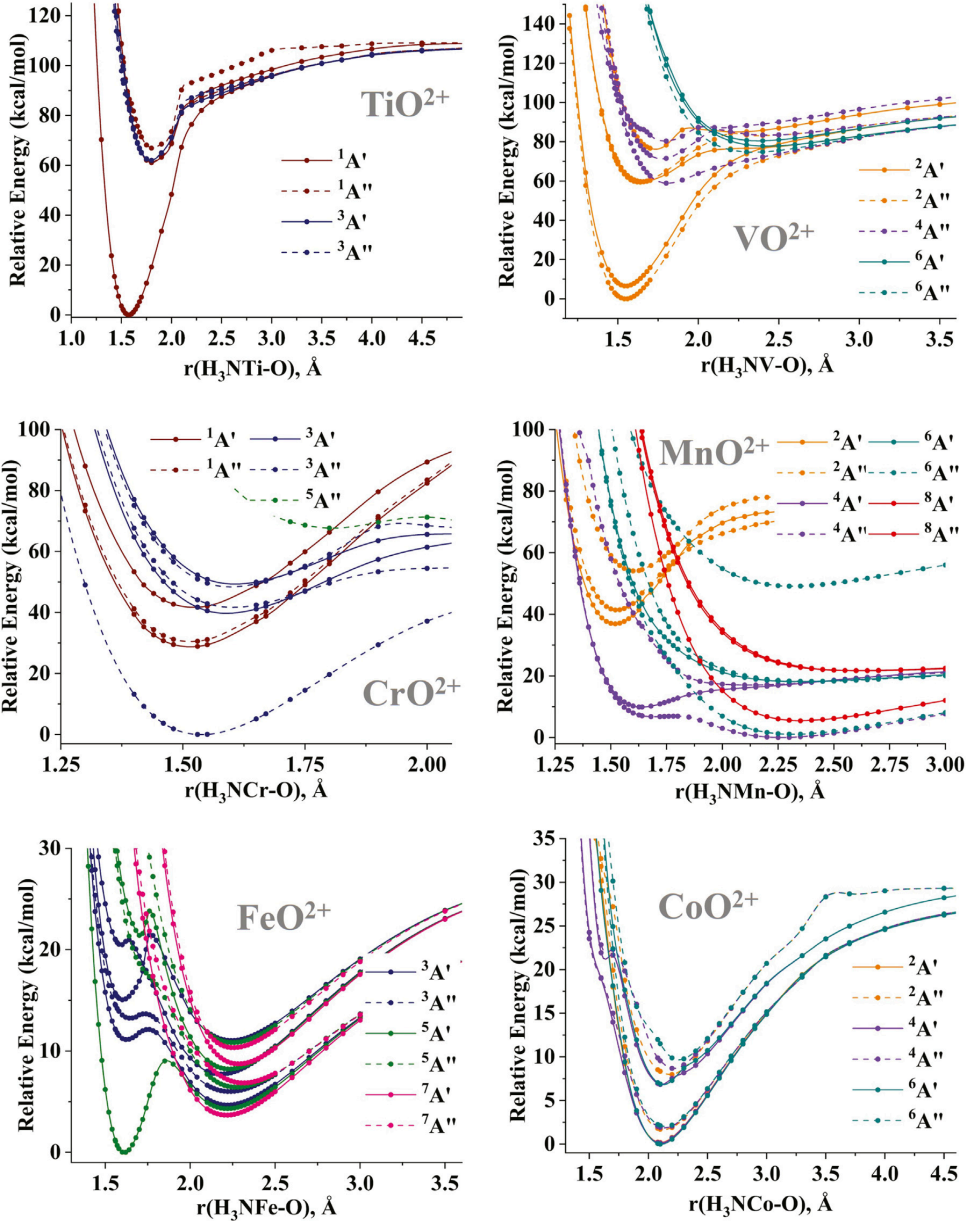
MRCI potential energy curves as a function of the M-O distance for the (NH_3_)MO^2+^ species, M = Ti-Co.

The potential energy curves (PECs) for the MO stretching of the (NH_3_)MO^2+^ species have been constructed for several electronic states. The CASSCF PECs are shown in Supplementary Figure S1 for all metals from Ti to Cu, and the MRCI PECs are given in Figure 3 for Ti through Co. The PECs for the MO^2+^ diatomics are reported in Claveau and Miliordos (2021). The addition of ammonia appears to have a smaller effect on the early transition metals (Ti-Cr). The morphology of the PECs remains the same before and after the inclusion of ammonia, with the difference being that the lower symmetry of (NH_3_)MO^2+^ leads to the splitting of the degenerate states of MO^2+^. For example, the ^1,3^Π and ^1,3^Φ (σ_MO_^2^π_MO_^3^π_MO_*^1^) excited states of TiO^2+^ split into the ^1,3^Α′ and ^1,3^Α″ components of (NH_3_)TiO^2+^, while the PEC of the ground state ^2^Δ for VO^2+^ separates into the lowest two ^2^Α′ and ^2^Α″ PECs of (NH_3_)VO^2+^. The splitting of the latter PECs is as large as 10 kcal/mol. On the other hand, the splitting for the ^1^Γ excited state of CrO^2+^ is only 2 kcal/mol.

The position of the M^3+^O^•−^ states relative to the ground M^4+^O^2−^ states shifts to higher energies. Specifically, the ^1^Σ^+^/^1^A′ (M^4+^O^2−^) → ^1^Φ/^1^A′ (M^3+^O^•−^) transitions for TiO^2+^/(NH_3_)TiO^2+^ are approximately 50–60 kcal/mol. The same effect is observed for VO^2+^/(NH_3_)VO^2+^ [^2^Δ/^2^(Α′, Α″) vs. ^4^Δ/^4^Α″]. For (NH_3_)CrO^2+^, the first excited oxo ^1^Γ/^1^(Α′,Α″) states are clearly lower than the first oxyl ^3^Σ^−^/^3^Α″ states in contrast with CrO^2+^. This stabilization of oxo states over the oxyl states becomes more obvious for the middle transition metals (M = Mn, Fe). The ^4^Π oxo state of MnO^2+^ is more than 20 kcal/mol higher than the clustered ^4,6,8^Σ^−^ lowest oxyl states [see Figure 1 of Claveau and Miliordos (2021)] but creates the local and global minima of the lowest energy ^4^A″ and ^4^Α′ PECs, respectively, of (NH_3_)MnO^2+^ (see Figure 3). These are less than 13 kcal/mol higher than the global minimum of the oxyl character of the ground ^4^A″ state.

The case of iron is even more interesting because the ^5^Σ^+^ oxo state of FeO^2+^ is 13 kcal/mol higher than the ^3^Δ ground oxyl state and becomes the ground ^5^A′ state of (NH_3_)FeO^2+^ separated by 4 kcal/mol from the first oxyl ^7^A′ state [pertaining to the ^7^Δ state of FeO^2+^; see Figure 1 of Claveau and Miliordos (2021)]. This stabilization of the oxo state has been observed in the literature for the (NH_3_)FeO^2+^/FeO^2+^ pair (Kirkland et al., 2018), but the present PECs are more complete, as they include more states, such as the aforementioned ^7^A′ state. In addition, comparing the CASSCF and MRCI PECs (Figure 3; Supplementary Figure S1) for iron, we see that the dynamic electron correlation is responsible for stabilizing the oxo states. Finally, in some of the PECs of (NH_3_)CoO^2+^ (^4^A′ and ^4^Α″), we see the appearance of local minima and shoulders at 1.6–1.7 Å and 20–23 kcal/mol, which are of oxo character. These were not present in CoO^2+^ and are expected to be stabilized even further when more ammonia ligands are coordinated to cobalt. The CASSCF/MRCI PECs for (NH_3_)CoO^2+^ show these shoulders at energies of approximately 40/20 kcal/mol above the global minimum. Such shoulders are not present at the CASSCF PECs for (NH_3_)NiO^2+^ and (NH_3_)CuO^2+^ (not at least within the 60 kcal/mol energy range of Supplementary Figure S1), and oxo minima are not expected to play an important role.

### 3.2 (NH_3_)_5_MO^2+^ species

**FIGURE 4:**
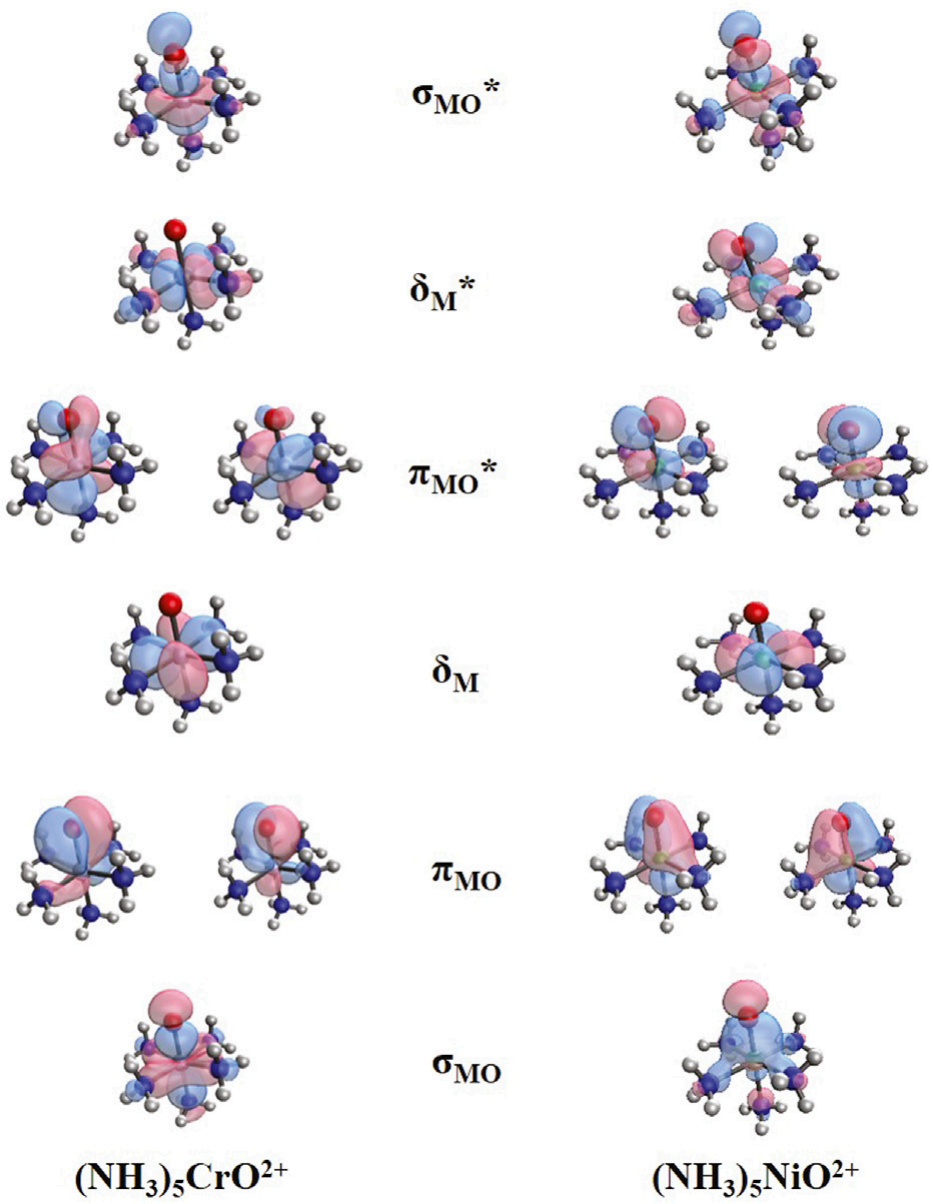
Frontier orbitals for (NH_3_)_5_CrO^2+^ and (NH_3_)_5_NiO^2+^.

The addition of more ammonia ligands is expected to stabilize the electronic configurations corresponding to the oxo states. As a demonstration, we switch our discussion to the fully coordinated (NH_3_)_5_MO^2+^ species. Figure 4 depicts the frontier orbitals of (NH_3_)_5_CrO^2+^ and (NH_3_)_5_NiO^2+^. We use the notation σ_MO_, π_MO_, σ_MO_*, π_MO_*, δ_Μ_, and δ_Μ_* to facilitate the discussion and compare directly with the smaller metal oxide systems. The main difference going from MO^2+^ to (NH_3_)_5_MO^2+^ is that one of the two non-bonding δ_M_ orbitals of MO^2+^ and (NH_3_)MO^2+^ becomes an antibonding metal–ammonia orbital (δ_M_*), and thus, the two δ_M_ orbitals are no longer degenerate, and the δ_M_* moves to higher energy between π_MO_* and σ_MO_*. The σ_MO_ and π_MO_ orbitals of (NH_3_)_5_MO^2+^ are polarized toward oxygen, the δ_M_ and π_MO_* orbitals are equivalent to the t_2g_ orbitals for an O_h_ analog, and the δ_M_* and σ_MO_* correspond to the e_g_ orbitals. Note that the π_ΜΟ_ orbitals are polarized toward oxygen for both early and late transition metals, which is different from MO^2+^/(NH_3_)MO^2+^; see, for example, π_NiO_∼*3d*_Ni_ [see Figure 2 of Claveau and Miliordos (2021)]. This is another indication that ammonia ligands stabilize the oxo character because the σ_MO_^2^π_MO_^4^ configuration can be assigned to M^4+^O^2−^ for both early and late transition metals. The π_MO_* orbitals have a higher contribution from the *3d* orbitals of the metal for (NH_3_)_5_CrO^2+^ as opposed to (NH_3_)_5_NiO^2+^, which has a higher contribution from the *2p* of oxygen. This is similar to the bare MO^2+^ species.

The metal–oxygen bond lengths (r_MO_), relative energies, and electronic configurations for the lowest energy states of various spin multiplicities obtained with DFT are listed in Table 1. It should be noted that higher energy states for each spin multiplicity should generally exist in the energy range covered in Table 1 and are not currently included.

**TABLE 1:** Metal–oxygen bond length (r_MO_ in Å), relative energies (ΔE in kcal/mol), electronic configurations (see Figure 4 for orbitals), and Mulliken spin density on the oxygen atom (ρ_S_/O) for the lowest energy spin states of (NH_3_)_5_MO^2+^ species, M = Ti-Cu, at the MN15 level of theory.

Species	S	r_MO_	ΔE	σ_MO_	π_MO_	π_MO_	δ_M_	π*_MO_	π*_MO_	δ*_M_	σ*_MO_	ρ_S_/O
(NH_3_)_5_TiO^2+^	0	1.579	0.0	2	2	2						0.00
	1	1.841	55.7	2	2	α	α					0.95
(NH_3_)_5_VO^2+^	1/2	1.547	0.0	2	2	2	α					0.18
	3/2	1.832	54.2	2	2	α	α	α				0.96
(NH_3_)_5_CrO^2+^	0	1.520	0.0	2	2	2	2					0.00
	1	1.613	1.8	2	2	2	α	α				0.38
	2	1.875	20.2	2	2	α	α	α	α			0.99
(NH_3_)_5_MnO^2+^	1/2	1.558	4.2	2	2	2	2	α				0.06
	3/2	1.620	0.0	2	2	2	α	α	α			0.62
	5/2	1.810	13.6	2	2	α	α	α	α	α		0.96
(NH_3_)_5_FeO^2+^	0^a^	1.603	7.6	2	2	2	2	α	β			0.00
	1	1.602	0.0	2	2	2	2	α	α			0.84
	2	1.594	1.1	2	2	2	α	α	α	α		0.81
	3	1.878	23.3	2	2	α	α	α	α	α	α	1.45
(NH_3_)_5_CoO^2+^	1/2	1.768	0.0	2	2	2	2	2	α			1.01
	3/2	1.643	4.5	2	2	2	2	α	α	α		1.18
	5/2	1.756	22.7	2	2	2	α	α	α	α	α	1.64
(NH_3_)_5_NiO^2+^	0^a^	1.759	1.0	2	2	2	2	2	α	β		1.14
	1	1.761	0.0	2	2	2	2	2	α	α		1.13
	2	1.777	9.2	2	2	2	2	α	α	α	α	1.77
(NH_3_)_5_CuO^2+^	1/2	1.844	0.4	2	2	2	2	2	α	2		1.06
	3/2	2.546	0.0	2	2	2	2	2	α	α	α	1.94

^a^ The spin contamination for these two open-shell singlets is 0.66 and 0.39 for (NH_3_)_5_FeO^2+^ and (NH_3_)_5_NiO^2+^, respectively.

The electronic structure of (NH_3_)_5_TiO^2+^ and (NH_3_)_5_VO^2+^ closely resembles the structures of the plain or mono-ligated metal oxide dications. The ground states retain their σ_MO_^2^π_MO_^4^ and σ_MO_^2^π_MO_^4^δ_M_^1^ oxo character being 50–60 kcal/mol away from the σ_MO_^2^π_MO_^3^δ_M_^1^ and σ_MO_^2^π_MO_^3^δ_M_^1^π_MO_^*1^ oxyl states. The bond lengths reflect the bonding patterns: the oxo states have r_MO_ = 1.579 Å and 1.547 Å, whereas for the oxyl states, we obtained r_MO_ = 1.841 Å and 1.832 Å.

The electronic structure of the (NH_3_)_5_CrO^2+^ lowest energy states is affected by the destabilization of δ_M_*. The ground state (^3^Σ^−^) of CrO^2+^ has a σ_MO_^2^π_MO_^4^δ_M_^1^δ_M_*^1^ configuration (Claveau and Miliordos, 2021), but the lowest energy triplet of (NH_3_)_5_CrO^2+^ adopts the σ_MO_^2^π_MO_^4^δ_M_^1^π_MO_*^1^ configuration, that is, δ_M_* is replaced by the lower energy π_MO_* orbital, which is still highly localized on Cr (see Figure 4). However, the introduced antibonding orbital pushes the triplet state of (NH_3_)_5_CrO^2+^ higher in energy than the singlet state (σ_MO_^2^π_MO_^4^δ_M_^2^), which was an excited state for CrO^2+^ with more than 30 kcal/mol excitation energy. CrO^2+^ has two low-lying singlet states (^1^Γ, ^1^Σ^+^) made of all three σ_MO_^2^π_MO_^4^(δ_M_δ_M_*)^2^ singlet spin configurations (Claveau and Miliordos, 2021). Only the σ_MO_^2^π_MO_^4^δ_M_^2^ configuration survives (no population of antibonding orbitals) for (NH_3_)CrO^2+^ and is the dominant configuration for the ground singlet state. The singlet and triplet states of (NH_3_)_5_CrO^2+^ are within 1.8 kcal/mol and can be both categorized as oxo states with bond lengths of 1.520 (S = 0) Å and 1.613 (S = 1) Å. On the other hand, the quintet state of (NH_3_)_5_CrO^2+^ has a bond length of 1.875 Å and a configuration σ_MO_^2^π_MO_^3^δ_M_^1^π_MO_*^2^, which makes it an oxyl state, and it is higher than the ground state by 20.2 kcal/mol.

The electronic structure changes dramatically for manganese as well. The (NH_3_)_5_MnO^2+^ complex has oxo ground states as opposed to MnO^2+^ and (NH_3_)MnO^2+^, which have oxyl ground states and low-lying oxo states. The lowest doublet and quartet states of (NH_3_)_5_MnO^2+^ have σ_MO_^2^π_MO_^3^δ_M_^2^π_MO_*^1^ and σ_MO_^2^π_MO_^4^δ_M_^1^π_MO_*^2^ configurations, respectively, with bond lengths 1.558 Å and 1.620 Å. The quartet state is the ground state separated by 4.2 kcal/mol from the doublet state. The sextet oxyl state is 13.6 kcal/mol higher with a bond length of 1.810 Å and a σ_MO_^2^π_MO_^3^δ_M_^1^π_MO_*^2^δ_M_*^1^ configuration. Overall, the oxo and oxyl states remain competitive, but the oxo states are more stable in the (NH_3_)_5_MnO^2+^ complex.

The oxo states for iron are also stabilized further, going from (NH_3_)FeO^2+^ to (NH_3_)_5_FeO^2+^. The ^5^A′ state of (NH_3_)FeO^2+^ with a 1a′^2^2a′^2^1a′^2^3a′^1^2a′^1^4a′^1^3a′^1^ (see Figure 2) ∼ σ_MO_^2^π_MO_^4^δ_M_^1^π_MO_*^2^δ_M_*^1^ configuration (see Figure 4) remains among the lowest energy states, but two other states, a singlet and a triplet, where the two 3a′^1^3a′^1^ (∼δ_M_^1^δ_M_*^1^) electrons couple together in the δ_M_ orbital. The two π_MO_*^2^ electrons couple into an open-shell singlet or a triplet spin state. All three states are within 8 kcal/mol, and they have very similar r_MO_ values (1.60 ± 0.01 Å). The equatorial Fe-N bond lengths for the quintet state are longer, though (2.17 ± 0.02 Å vs. 2.03 Å), due to the population of the δ_M_* orbital, which has M-N antibonding character (see Figure 4). The open-shell singlet state has considerable spin contamination, and a more accurate description of the wave function necessitates applying multireference methods. The septet state is the lowest energy oxyl state, similar to (NH_3_)FeO^2+^, with a σ_MO_^2^π_MO_^3^δ_M_^1^π_MO_*^2^δ_M_*^1^σ_MO_*^1^ configuration.

The lowest energy state of (NH_3_)_5_CoO^2+^ is a doublet state with a bond length of 1.768 Å. This length lies in between the bond lengths encountered so far for oxo (∼1.6 Å) and oxyl (>1.8 Å) states. Its configuration is σ_MO_^2^π_MO_^4^δ_M_^2^π_MO_*^3^, but now the π_ΜΟ_* orbital has a larger contribution from the *2p* of oxygen, similar to the (NH_3_)_5_NiO^2+^ orbitals of Figure 4. Therefore, this is the first oxyl state not corresponding to a half-filled π_MO_ orbital; it has considerable Mulliken spin density on oxygen (1.0 electron). All the π_MO_^3^ states mentioned above have a spin density of 0.95 (except the S = 3 of iron, which has 1.45) on oxygen. In the quartet state, one electron moves from π_MO_* to δ_M_*, which leads to the contraction of the MO bond to 1.643 Å and corresponds to the configuration of the shoulders observed in the PECs of (NH_3_)CoO^2+^ at around 20–25 kcal/mol (see above). Finally, the sextet state follows at 22.7 kcal/mol and populates σ_MO_* (spin density of 1.6).

Finally, all studied states of the last two oxides, (NH_3_)NiO^2+^ and (NH_3_)CuO^2+^, have long bond lengths (>1.75 Å), and their configurations have σ_MO_^2^π_MO_^4^δ_M_^2^ in common. The remaining electrons populate the π_MO_* and δ_M_* orbitals, and the σ_MO_* is populated only for the highest spin states. The metal–oxygen bond in the quartet state of (NH_3_)CuO^2+^ is clearly ruptured and is better described as (NH_3_)Cu^2+^+O (^3^P). In all of these states, the spin density on oxygen is between 1.1 and 2.0; the spin density for S = 3/2 of (NH_3_)CuO^2+^ is 1.94.

### 3.3 (NH_3_)_5_MO^2+^ + CH_4_/CH_3_OH reactions

**FIGURE 5:**
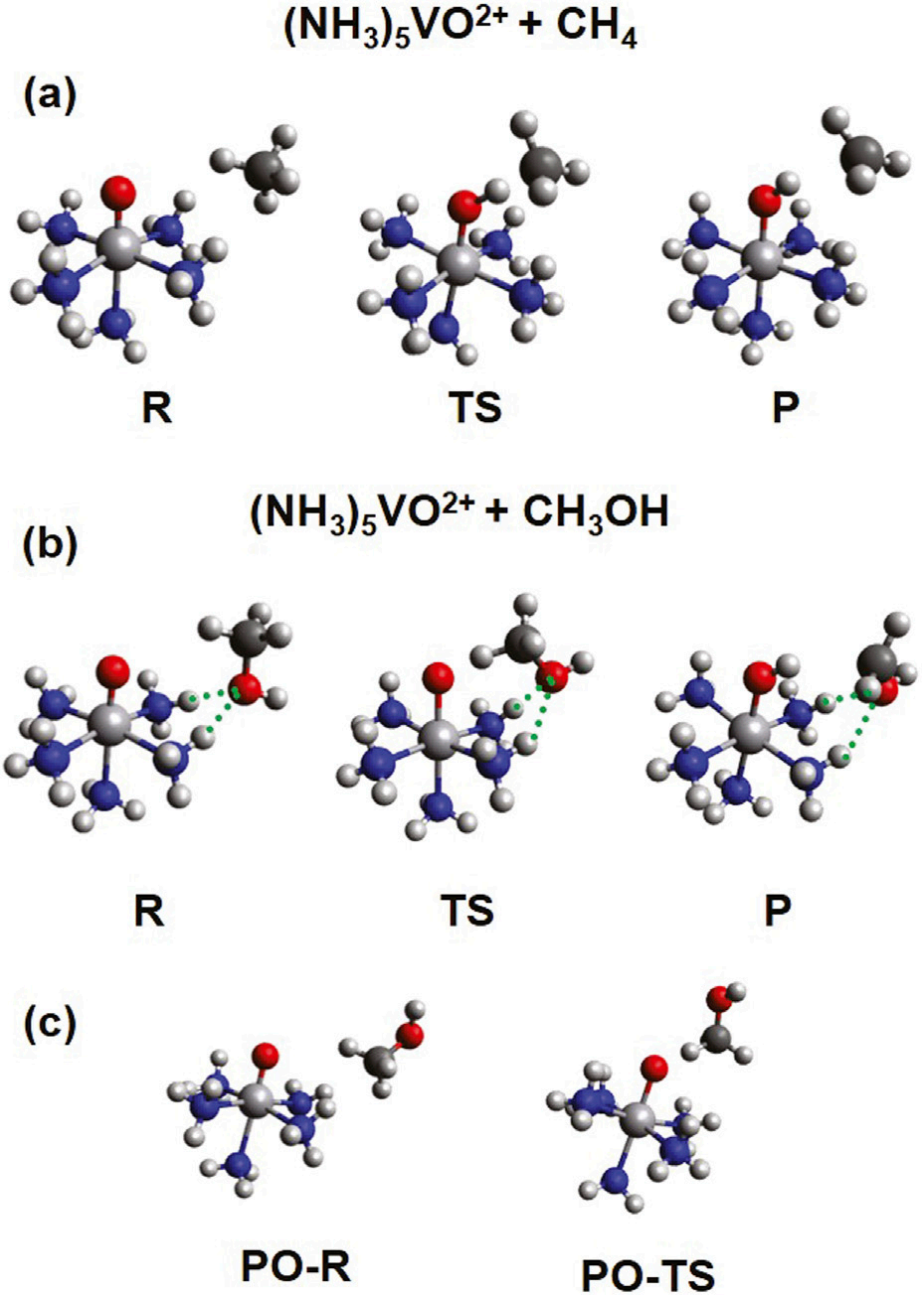
Structures for the reactants (R), transition states (TS), and products (P) of the reaction between (NH_3_)_5_VO^2+^ (S = 1/2) and CH_4_ **(A)** or CH_3_OH **(B)**. The structures in **(C)** correspond to partially optimized (PO) species for the reaction with CH_3_OH, where the OH group points away from the ammonia ligands (see text for more details).

Typical structures of the encounter complex of the reactants (R), the transition state (TS), and the encounter complex of the products (P) are shown in Figure 5. One H atom is transferred from CH_4_ or CH_3_OH via either HAT or PCET, and thus, a CH_3_ or CH_2_OH radical is released as a product. Ideally, the reaction with methanol should be slower in order to avoid overoxidation of methanol and increase its yield. However, because the C-H bond of methane is stronger than that of methanol, the activation barrier for methanol is predisposed to be lower, posing significant difficulties in the search for selective catalysts. Recent experiments showed that establishing a hydrophobic ligand environment can prevent methanol from approaching the metal center and increase the selectivity against formaldehyde (Fujisaki et al., 2023).

We suggested a different strategy in our recent computational work: The formation of hydrogen bonds between the OH group of methanol and the ligands can be exploited to increase the activation energy for methanol (Claveau et al., 2023a). Ammonia ligands can serve this role and are currently used as a model system. More applicable ligands, like the ones used to functionalize specific C-H bonds of complex organic by exploiting hydrogen bonding interactions (Olivo et al., 2017), will be explored in a future quest. Additionally, solvent effects are an important factor. For example, polar/non-polar solvents may disrupt/reinforce the hydrogen bonds between methanol and ligands.

As seen in Figure 5, in the case of (NH_3_)_5_MO^2+^, the OH group of methanol makes two hydrogen bonds with two different ammonia ligands. To assess the importance of the formation of these hydrogen bonds, we considered some structures where the OH group points away from the ammonia ligands. All our efforts to obtain fully optimized structures with the OH away from ammonia ligands resulted in the formation of hydrogen bonds. Therefore, we started from the reactants and transition states of methane and replaced one H atom with an OH group, as shown in Figure 5C. The OH unit is optimized, but every other atom is kept fixed. These structures will be referred to as partially optimized (PO).

**FIGURE 6:**
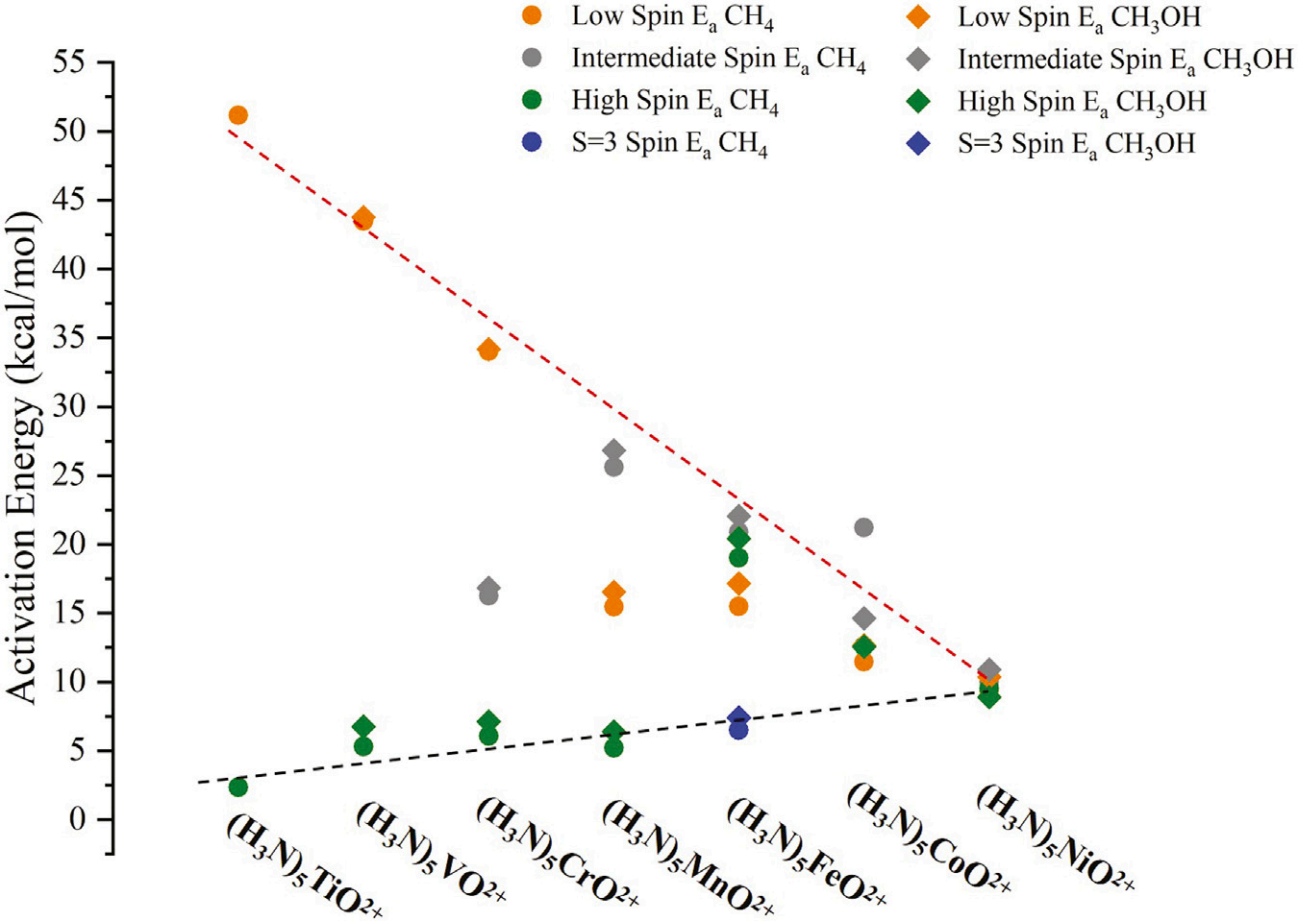
Activation energies (E_a_) for the reactions of (NH_3_)_5_MO^2+^ with CH_4_ and CH_3_OH for M = Ti-Ni and for LS, IS, and HS spin states, and S = 3 of M = Fe. The dashed red/black lines show the trend for oxo/oxyl states. All numerical values are listed in Supplementary Table S10 of the SI.

All our structures and energies are given in the SI. The resulting activation energies E_a_, calculated as the energy difference between the TS and R energies, are plotted in Figure 6. These include all different spin states: low spin (LS; the lowest spin of Table 1), high spin (HS; the highest spin of Table 1; S = 2 for iron), and intermediate spin (IS; the spin between HS and LS of Table 1). The S = 3 state of (NH_3_)_5_FeO^2+^ is also included. In all cases (except Ti/HS and Co/IS), E_a_ values are competitive (within 1.7 kcal/mol) for methane and methanol, and for these cases, the E_a_ value for methanol is larger. We could not get the TS for Ti/HS of methanol, and we are unsure why the E_a_ for methanol is lower than for methane by 6.4 kcal/mol in the case of Co/IS. These results indicate a different trend from the literature reports. For example, Nørskov and co-workers found that the E_a_ values for methanol are always 0.57 eV (13.1 kcal/mol) lower than methane for a large number/variety of heterogeneous catalysts. (Latimer et al., 2018). Similar differences can be implied for molecular catalysts based on the selectivity plots in Ravi et al. (2017). Our results confirm that hydrogen bonding can be a general future promising strategy that needs to be further explored (Claveau et al., 2023a).

The largest activation barriers for each metal correspond to states that have been characterized as oxo states, that is, LS of (NH_3_)_5_TiO^2+^ and (NH_3_)_5_VO^2+^, LS/IS for (NH_3_)_5_CrO^2+^ and (NH_3_)_5_MnO^2+^, and LS/IS/HS(S = 2) for (NH_3_)_5_FeO^2+^. Interestingly, the E_a_ values for these states decrease in a linear fashion, going from early to late transition metals, which indicates that the radical character of the oxo states increases. This is also confirmed by the Mulliken spin densities on oxygen for these states: 0.00 (Ti/LS), 0.18 (V/LS), 0.00 (Cr/LS), 0.38 (Cr/IS), 0.06 (Mn/LS), 0.62 (Mn/IS), 0.00 (Fe/LS), 0.84 (Fe/IS), and 0.81 (Fe/HS). The Fe/LS state is the open-shell singlet sister state of Fe/IS, and thus, the spin density is 0.00 due to the cancelation of the two π_ΜΟ_* electron spins.

Notice that the 0.38 spin density of Cr/IS is responsible for lowering the barrier to less than half compared to the Cr/LS with 0.00 spin density. In contrast, although Mn/IS has a higher spin density on oxygen than Mn/LS (0.62 vs. 0.06), it has a higher activation barrier (25.6 vs. 15.5 kcal/mol). The reason is that the Mn/LS has a substantial contribution of a σ_MO_^2^ π_MO_^3^ δ_M_^2^ π*_MO_^2^ (in addition to the equilibrium σ_MO_^2^ π_MO_^4^ δ_M_^2^ π*_MO_^1^) electronic configuration at the transition state geometry. The partially occupied π_MO_ is the main reason for the lowering of the barrier, as happens for the Mn/HS (σ_MO_^2^ π_MO_^3^ δ_M_^1^ π*_MO_^2^ δ_M_*^1^). This observation implies that a better quantity to consider is the spin density of the oxygen atom at the transition state of each species. These values are given in Supplementary Table S11. Indeed, the spin density for Mn/LS has dropped from 0.62 to 0.52, and that of Mn/IS has increased from 0.06 to 0.44. Plotting the average spin densities against the average E_a_ values over all oxo states for each species, we see a better correlation between E_a_ and transition state spin density on oxygen (see Supplementary Figure S4).

**FIGURE 7:**
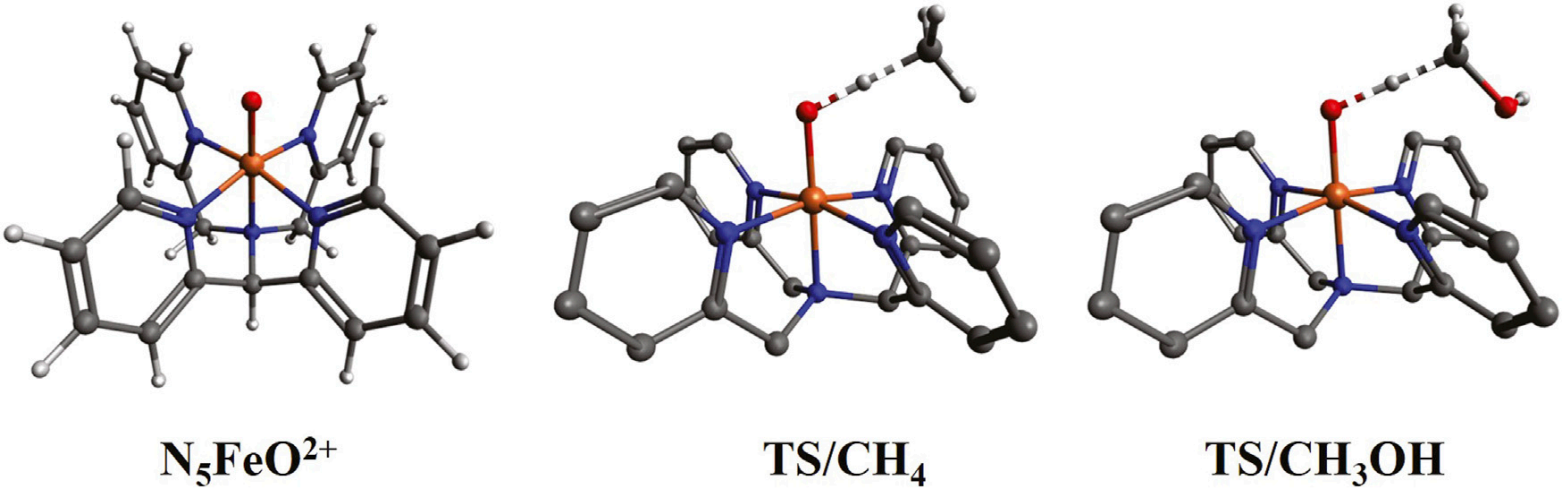
Optimized structures for N_5_FeO^2+^ (triplet ground state) and the transition states (TS) of its reaction with CH_4_ and CH_3_OH. The hydrogen atoms of N_5_FeO^2+^ are not shown in the TS structures.

A different explanation can be provided based on the potential energy curves of Claveau et al. (2023b) along the reaction coordinate of (NH_3_)_5_TiO^2+^ + CH_4_. According to Figure 7 and the corresponding discussion of Claveau et al. (2023b), the activation barrier of the ground oxo state (Ti/LS/S = 0) is controlled by the excitation energy to the oxyl state (Ti/HS/S = 1). The lower the excitation energy, the lower the E_a_ value is expected to be. Indeed, the oxo → oxyl energy difference drops as we go from Ti to Mn (55.7, 54.2, 20.2, and 13.6 kcal/mol; see Table 1) but increases for Fe (23.3 kcal/mol). Overall, the combination of the spin density on the oxygen atom (preferably at the transition states) and oxo → oxyl excitation energies can be considered good descriptors for predicting the activation barriers of the reaction of metal oxides with methane.

On the other hand, the E_a_ values for the remaining states of clear oxyl character are very small (on the order of 10 kcal/mol or smaller) and present an increasing trend. The spin density on the oxygen atom is 0.95 or higher for these cases due to the partial occupation of the π_ΜΟ_ (for M = Ti-Fe) or π_ΜΟ_* (for M = Co, Ni) orbitals (see Table 1), which are primarily composed of the 2p orbitals of oxygen (see Figure 4). The two lines (oxo and oxyl) converge to the same values for (NH_3_)_5_NiO^2+^ (see Figure 6).

The reason for the unprecedently high activation barriers for methanol has been ascribed to the disruption of both the hydrogen bonds and the “stress” imposed on the activated CH bond going from the reactants to the TS, as expressed by the increased hydrogen bond distances and the decreased OCH angles (Claveau et al., 2023a). The same observations can be made here. The latter angles range between 176° and 180° for CH_4_ (no hydrogen bonding) and between 166° and 175° for CH_3_OH (see Supplementary Figure S2). Finally, we compare the activation barriers for methanol using the fully and partially optimized structures for R and TS (see Figure 5). In several cases, the E_a_ values for the partially optimized structures are negative because these structures do not correspond to actual stationary structures. The E_a_ values for the two cases and their differences are plotted in Supplementary Figure S3. The difference between the two structures is remarkably consistent across the various metals and spin states ranging between 17 kcal/mol and 22 kcal/mol. This is a very large difference that can play an important role in developing strategies for the selective formation of methanol.

### 3.4 (NH_3_)_5_FeO^2+^ vs. N_5_FeO^2+^

In this section, we compare the all-ammonia iron oxide complex with the experimental structures of Rasheed et al. (2018), which bear tertiary and pyridinic N-ligands (see Figure 7). This comparison serves two purposes. First, we assess the use of ammonia as a simple model ligand to replace tertiary and pyridinic N-ligands, and second, we calculate the activation barriers for methane and methanol in a ligand environment (N_5_FeO^2+^) where hydrogen bond formation is not possible.

The lowest energy state of N_5_FeO^2+^ is the triplet, followed closely by the quintet (at 6.2 kcal/mol) and singlet (at 7.2 kcal/mol) states. The septet is well separated at 32.0 kcal/mol. This pattern is identical to (NH_3_)_5_FeO^2+^, and the numerical values agree well for the two species: 1.1 kcal/mol vs. 6.2 kcal/mol, 7.6 kcal/mol vs. 7.2 kcal/mol, and 23.3 kcal/mol vs. 32.0 kcal/mol (see Table 1). Overall, the electronic effects associated with the pyridinic rings (possible π-back bonding) have some effect on the electronic structure of the iron oxide unit, but ammonia can clearly provide big-picture insights at a significantly lower computational cost. Of course, ammonia ligands cannot account for steric effects or other types of interactions (such as dispersion) present in larger ligands (see next paragraph). In summary, we believe that the relative energetics provided in Table 1 for the various spin states of (NH_3_)_5_MO^2+^ are valid for the N_5_MO^2+^ species as well.

The transition states for the reaction of N_5_FeO^2+^ with methane and methanol are depicted in Figure 7. The two molecules approach N_5_FeO^2+^ with the same orientation because the OH group of methanol interacts only weakly with the pyridinic rings. The OH/CH distances at the transition states are 1.20/1.33 Å and 1.28/1.28 Å for methane and methanol, respectively, indicating that the H atom departs “earlier” from methanol. The activation barriers for methane are 24.6 kcal/mol and 24.5 kcal/mol for the triplet and quintet states, which are higher by 3.7 kcal/mol and 5.5 kcal/mol than (NH_3_)_5_FeO^2+^ (see Supplementary Table S10), possibly because of the dispersion interactions between CH_4_ and the π-system of the pyridinic rings. The activation barriers for methanol of 22.5 kcal/mol (for both spin multiplicities) are lower than methane, signifying again the importance of hydrogen bonding in the (NH_3_)_5_FeO^2+^ species.

## 4 Conclusion

In this work, we studied the electronic structure and reactivity of the dicationic transition metal oxide complexes (NH_3_)_5_MO^2+^, where M is a first-row transition metal. We started by extending our previous work on the bare metal oxide unit, MO^2+^, to the mono-ligated (NH_3_)MO^2+^ species. In both cases, we have constructed potential energy curves along the M-O distance for the ground and numerous excited electronic states. We covered a sufficiently wide energy range to include both oxo (M^4+^O^2−^) and oxyl (M^3+^O^⋅−^) electronic states. We concluded that the ammonia ligand stabilizes the oxo states. For the early transition metals (Ti, V, and Cr), the oxo states, already lower in energy for MO^2+^, become more well separated from the oxyl states. For the middle transition metals (Mn, Fe), the oxo states (higher in energy for MO^2+^) become energetically competitive with the oxyl states (Mn) or the ground state (Fe). The lowest states for the late transition metals remain of oxyl character, but for Co, we see some shoulders in the repulsive region of the potential energy curves corresponding to oxo electronic configurations. The dynamic electron correlation (at the MRCI level) was found to be important for the stabilization of the oxo states.

We then switched to the fully coordinated (NH_3_)_5_MO^2+^ complexes, where we applied DFT to study the lowest energy state of low-, intermediate-, and high-spin multiplicity. The coordination of more ammonia ligands stabilized the oxo states even further. For both early and middle transition metals, the ground states are of oxo character (low and intermediate spin), followed by the high-spin oxyl states at energies ∼55 kcal/mol for Ti and V and 14–23 kcal/mol for Cr, Mn, and Fe. The coordination of five ammonia ligands to CoO^2+^ stabilized the states observed as shoulders in (NH_3_)CoO^2+^, creating a clear minimum (intermediate spin). Going from MO^2+^ to (NH_3_)MO^2+^, we see that the π_ΜΟ_* and one δ_M_ orbitals become the pseudo-t_2g_ orbitals, and the σ_MO_* and the other δ_M_ (δ_Μ_*) become the pseudo-e_g_ ones. Therefore, the low-lying electronic states of (NH_3_)_5_MO^2+^ can be generally predicted from these of MO^2+^, considering that the energy of the MO^2+^ states that populate the δ_Μ_* increases appreciably.

We then probed the reactivity of all studied (NH_3_)_5_MO^2+^ electronic states regarding the activation of methane and methanol. We found the activation barriers for the oxo states are considerably higher for the early transition metals. The barriers for the oxo states drop as we move to the later transition metals. The barriers for the oxyl states are small (<10 kcal/mol) and show a slightly increasing trend as we move from the early to the late transition metals. Based on the spin density on the oxygen atom, we ascribed these trends to the fact that the oxyl character of the oxo states increases as we move to the later metals. Therefore, the two sets of barriers converge almost linearly, going from Ti to Ni.

The reaction with methanol, rarely studied in the literature, is important for identifying catalysts for the selective transformation of methane to methanol. Catalysts with higher activation barriers for methanol will slow its overoxidation and avoid the formation of formaldehyde. Here, we show that the hydrogen bonds between the OH group of methanol and the ammonia ligands may increase the barrier for methanol by about 20 kcal/mol, rendering it slightly higher than (or at least competitive with) that of methane.

Finally, we performed calculations for the experimentally made N_5_FeO^2+^ complex, where five ligands (four pyridinic rings and one tertiary nitrogen) make coordinative bonds with iron via a nitrogen center, but they cannot form hydrogen bonds with methanol. We showed that the electronic structure and energetics of the lowest lying states of (NH_3_)_5_FeO^2+^ and N_5_FeO^2+^ follow the same pattern and that methanol goes through a higher activation barrier for the former and lower for the latter compared to methane.

Our future work will focus on more applicable ligands to assess the effect of all kinds of interactions (hydrogen bonding, dispersion, electrostatic, and others) in the activation barriers of methane and methanol to identify systems with much larger barriers for methanol. The role of solvent molecules will also be explored. We believe that our findings provide an additional arrow to the chemists’ quiver and will lead to the design of more practical ligands with even higher activation barriers for methanol.

## Data Availability

The original contributions presented in the study are included in the article/Supplementary Material; further inquiries can be directed to the corresponding author.
