# Supplementary material for: Electronic structure of metal oxide dications with ammonia ligands and their reactivity towards the selective conversion of methane to methanol
Source: Front Chem. 2024 Dec 11;12:1508515. doi: 10.3389/fchem.2024.1508515 (PMC11668566; doi:10.3389/fchem.2024.1508515)
Supplement: Supplementary file 1 [file DataSheet1.PDF]

Supporting Information for

Electronic Structure of Metal Oxide Dications with Ammonia Ligands and Their Reactivity Towards the Selective Conversion of Methane to Methanol

Emily E. Claveau† and Evangelos Miliordos*

Department of Chemistry and Biochemistry, Auburn University, Auburn, AL 36849-5312, USA

†Current address: Department of Chemistry, Michigan State University, East Lansing, Michigan 48824, U.S.A

*emiliord@auburn.edu

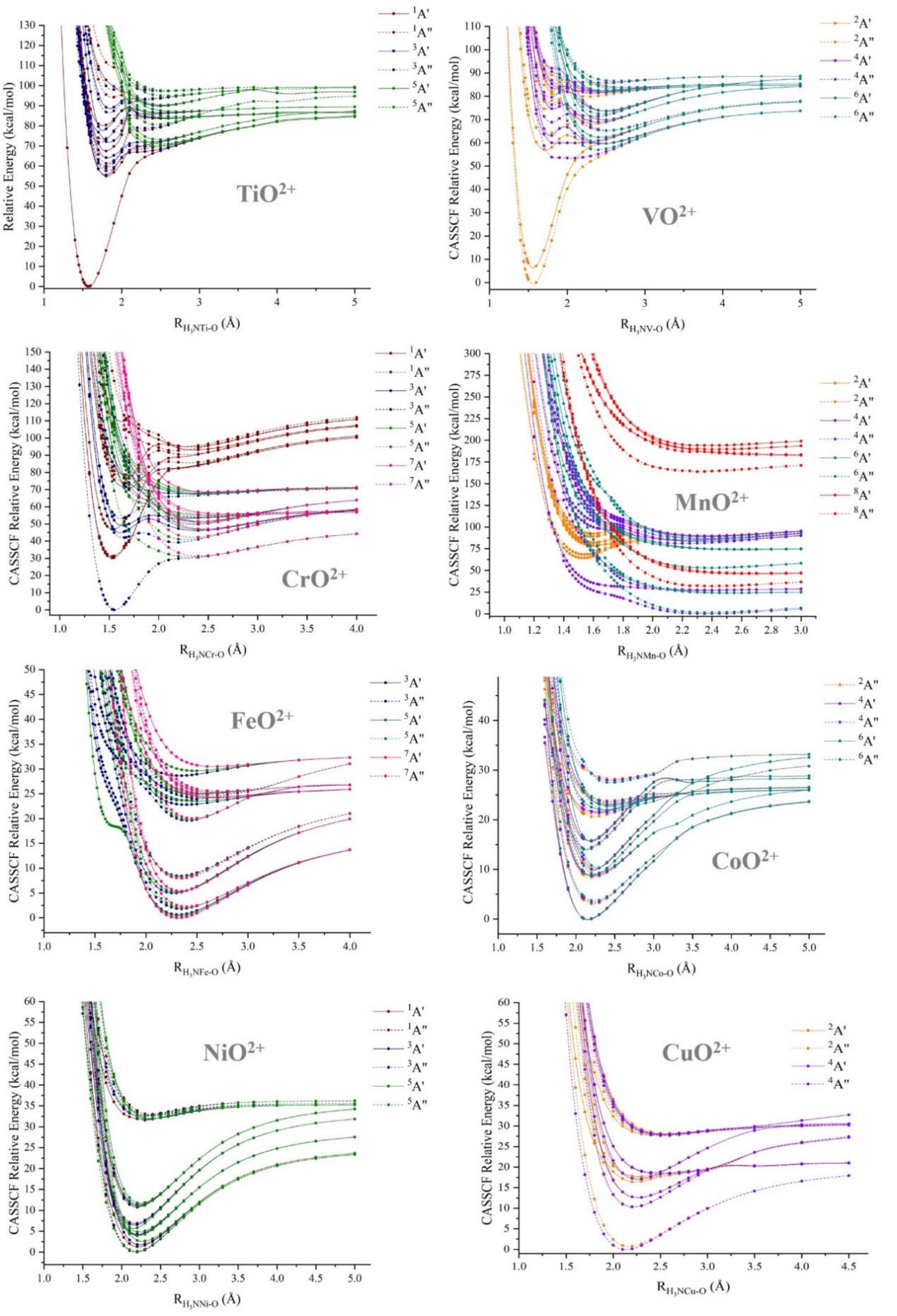

Figure S1. CASSCF potential energy curves as a function of the M-O distance for the $(NH_3)MO^{2+}$ species, M=Ti-Co.

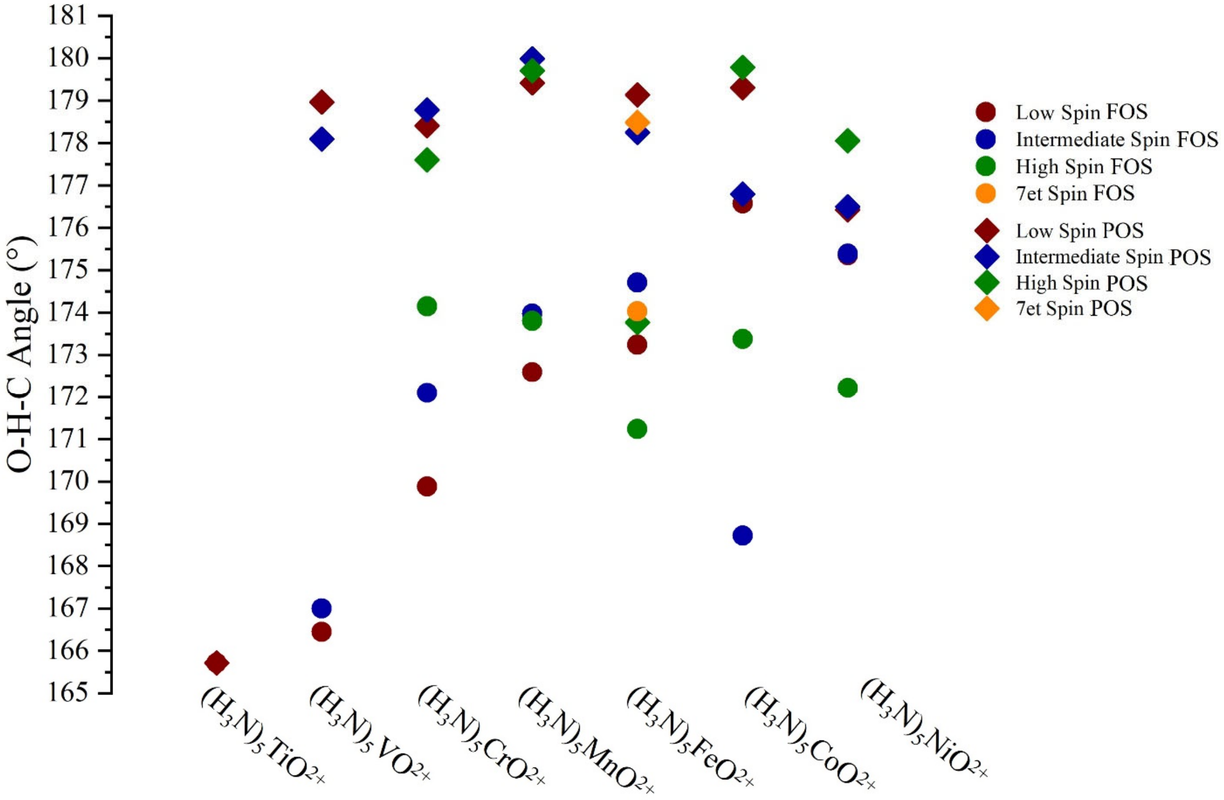

Figure S2. OCH angles for the fully optimized structures (FOS) and partially optimized structures (POS) of the transition states for the $(NH_3)MO^{2+}$ + CH_3OH reaction.

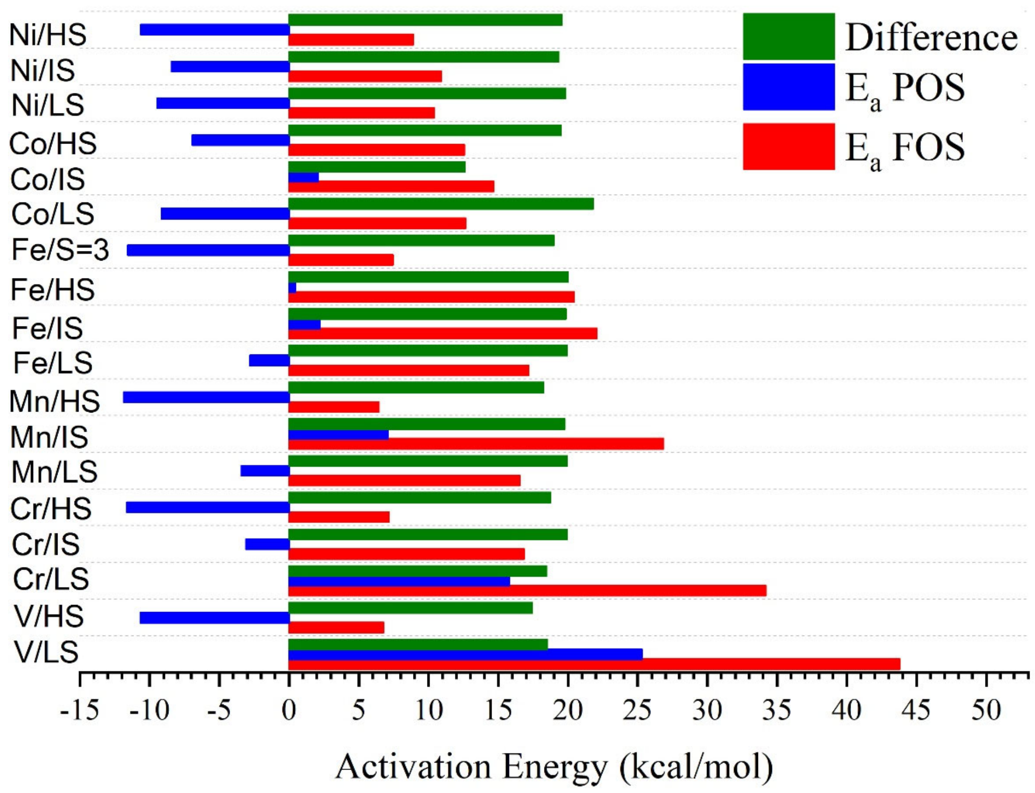

Figure S3. Activation energies (E_a values) for the fully optimized structures (FOS) and partially optimized structures (POS) of the transition states for the $(NH_3)MO^{2+}$ + CH_3OH reaction.

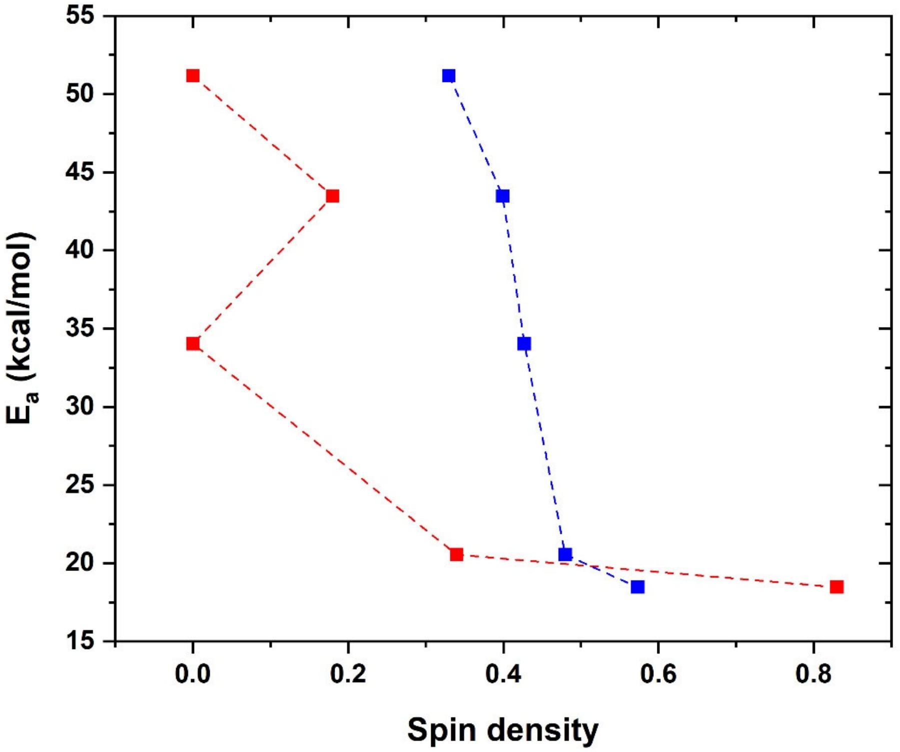

Figure S4. Activation energies (E_a values) against spin densities on oxygen at the reactants (red squares) and transition state (blue circles) structures of the $(NH_3)_5MO^{2+}$ + CH_4 reaction. The values are averaged over the oxo states of each species; see text.

Table S1. Optimized geometries (Cartesian coordinates in Å) for mono-ammonia ligated metal oxide dications.

	Optimized geometry
$H_3N\text{-}TiO^{2+}$ (S=0)	Ti 0.000000 0.587498 0.000000<br>O -1.414903 0.047295 0.000000<br>N 1.072465 -1.222394 0.000000<br>H 2.100282 -1.152949 0.000000<br>H 0.855845 -1.796807 0.829971<br>H 0.855845 -1.796807 -0.829971
$H_3N\text{-}VO^{2+}$ (S=1/2)	V 0.000000 0.508308 -0.000000<br>O -1.464454 0.231261 -0.000000<br>N 1.108824 -1.248044 0.000000<br>H 2.134516 -1.144057 0.000000<br>H 0.909677 -1.830404 0.830141<br>H 0.909677 -1.830404 -0.830141
$H_3N\text{-}CrO^{2+}$ (S=1)	Cr 0.000000 0.476408 0.000000<br>O -1.462107 0.242623 0.000000<br>N 1.105622 -1.233967 0.000000<br>H 2.129060 -1.098991 0.000000<br>H 0.914223 -1.819005 0.828511<br>H 0.914223 -1.819005 -0.828511
$H_3N\text{-}MnO^{2+}$ (S=3/2)	Mn 0.000000 0.545089 0.000000<br>O -1.466463 -0.157771 0.000000<br>N 1.108494 -1.137571 0.000000<br>H 2.124070 -0.950623 0.000000<br>H 0.924089 -1.725723 0.827144<br>H 0.924089 -1.725723 -0.827144
$H_3N\text{-}FeO^{2+}$ (S=3)	Fe 0.000000 0.534098 -0.000000<br>O -1.374389 -0.199199 0.000000<br>N 1.041257 -1.130873 0.000000<br>H 2.059606 -0.952485 0.000000<br>H 0.823352 -1.712186 0.826160<br>H 0.823352 -1.712186 -0.826160
$H_3N\text{-}CoO^{2+}$ (S=5/2)	Co 0.000000 0.285415 0.000000<br>O -2.079004 0.252159 0.000000<br>N 1.565863 -0.899404 0.000000<br>H 2.457123 -0.379869 0.000000<br>H 1.606934 -1.523890 0.819626<br>H 1.606934 -1.523890 -0.819626
$H_3N\text{-}NiO^{2+}$ (S=0)	Ni 0.000000 0.320261 0.000000<br>O -2.010942 0.057859 0.000000<br>N 1.515524 -0.870868 0.000000<br>H 2.409787 -0.352939 0.000000<br>H 1.534540 -1.490584 0.823567<br>H 1.534540 -1.490584 -0.823567
$H_3N\text{-}CuO^{2+}$ (S=3/2)	Cu 0.000000 0.502765 -0.000000<br>O -1.709139 -0.537910 0.000000<br>N 1.292341 -0.946927 0.000000<br>H 2.243460 -0.544375 -0.000000<br>H 1.191632 -1.552014 0.827609<br>H 1.191632 -1.552014 -0.827609

Table S2. Optimized geometries (Cartesian coordinates in Å) for penta-ammonia ligated metal oxide dications.

	S=0				S=1				S=2			
$(H_3N)_5TiO^{2+}$	Ti	0.002592	0.010252	-0.241796	Ti	0.004319	0.003632	-0.056174	Ti	-0.005776	0.005921	0.089259
	O	0.022974	0.074775	-1.819384	O	0.084712	0.042280	-1.894605	O	0.096574	-0.183168	-2.343833
	N	1.704498	1.427604	0.065003	N	2.238703	0.067525	0.204706	N	2.316393	0.003150	0.077733
	N	1.449686	-1.685643	-0.190759	N	0.101158	-2.199747	-0.470026	N	0.025581	-2.276326	-0.178903
	N	-0.059755	-0.170779	2.227378	N	-0.131848	-0.065127	2.246091	N	-0.110845	0.197294	2.355684
	N	-1.732710	-1.396033	-0.137954	N	-2.245547	-0.064695	0.001894	N	-2.317480	-0.012168	-0.117889
	N	-1.384413	1.747758	-0.067872	N	-0.038236	2.222277	-0.378443	N	0.026516	2.216940	-0.534902
	H	2.548929	1.026996	0.475303	H	2.686899	-0.738832	0.640645	H	2.724246	-0.762704	0.613839
	H	1.506308	2.257777	0.624556	H	2.636160	0.882480	0.672775	H	2.727192	0.846836	0.477368
	H	1.044987	-2.496880	-0.662505	H	-0.775040	-2.577172	-0.832448	H	-0.784309	-2.667063	-0.660761
	H	1.840846	-2.029975	0.685506	H	0.381881	-2.826332	0.285566	H	0.052566	-2.757358	0.721586
	H	-0.880381	0.255213	2.658304	H	-0.691805	0.694411	2.636978	H	-0.701545	0.969588	2.666252
	H	0.733086	0.260981	2.702338	H	0.763708	-0.013917	2.732557	H	0.784305	0.335760	2.824319
	H	-2.068334	-1.527660	-1.094302	H	-2.520514	-0.072965	-0.983001	H	-2.654304	-0.129247	-1.074200
	H	-2.538053	-1.085631	0.406599	H	-2.732419	0.732289	0.412717	H	-2.767549	0.842195	0.210274
	H	-1.014959	2.497625	-0.656120	H	0.709391	2.478719	-1.025816	H	0.853658	2.498679	-1.061668
	H	-1.604164	2.169189	0.834098	H	0.072186	2.837386	0.429010	H	0.018497	2.829022	0.282657
	H	1.980645	1.768405	-0.858190	H	2.605279	0.097599	-0.749293	H	2.740117	-0.075777	-0.847396
	H	2.242553	-1.434028	-0.784845	H	0.778554	-2.351396	-1.220221	H	0.837513	-2.630612	-0.685276
	H	-0.067867	-1.134257	2.562589	H	-0.574739	-0.918143	2.591967	H	-0.519182	-0.627272	2.798063
	H	-1.524567	-2.331784	0.212766	H	-2.685807	-0.887581	0.414154	H	-2.769760	-0.760071	0.407788
	H	-2.280991	1.519934	-0.502089	H	-0.896055	2.523680	-0.842473	H	-0.768118	2.520885	-1.097947
	S=1/2				S=3/2				S=5/2			
$(H_3N)_5VO^{2+}$	V	0.001678	0.008537	-0.237399	V	0.001495	-0.002446	-0.056525	V	-0.004444	0.000121	0.025053
	O	0.007275	0.050526	-1.783816	O	0.010358	-0.013142	-1.888005	O	0.204672	0.062178	-2.332620
	N	-2.142222	-0.322573	-0.137598	N	-2.153302	0.267466	0.081306	N	0.317536	2.207780	-0.085199
	N	0.370524	-2.127605	-0.103066	N	-0.263336	-2.131748	-0.377778	N	-2.195597	0.289089	-0.283338
	N	-0.033826	-0.122572	2.138599	N	-0.018409	0.008973	2.179808	N	-0.217887	-0.040751	2.222254
	N	2.135464	0.382355	-0.074290	N	2.156239	-0.256677	0.109512	N	-0.259216	-2.205092	-0.222260
	N	-0.339361	2.133183	0.047286	N	0.267685	2.125743	-0.394010	N	2.207337	-0.297045	0.082701
	H	-2.392544	-1.191959	-0.611010	H	-2.689893	-0.552567	0.366331	H	-0.403422	2.714330	-0.599660
	H	1.122986	-2.421705	0.519663	H	0.556835	-2.541795	-0.827119	H	-2.760057	-0.359911	0.265325
	H	0.880499	-0.283473	2.561571	H	0.757711	-0.505047	2.597866	H	-0.676653	-0.881705	2.572763
	H	2.375790	1.074390	-0.786466	H	2.513931	-0.205146	-0.847254	H	0.297048	-2.598360	-0.982646
	H	-0.370853	2.554778	-0.883144	H	0.415357	2.238638	-1.401344	H	2.664888	-0.299530	-0.830137
	H	-2.610280	-0.346379	0.767829	H	-2.492059	1.030520	0.667923	H	0.351350	2.655082	0.831114
	H	-2.612859	0.400915	-0.683716	H	-2.467384	0.481814	-0.868069	H	1.192356	2.473269	-0.538601
	H	-0.432733	-2.700360	0.157194	H	-0.476924	-2.732745	0.418786	H	-2.531540	1.214741	-0.017323
	H	0.635804	-2.441183	-1.038786	H	-1.017051	-2.274462	-1.053147	H	-2.512483	0.162854	-1.245729
	H	-0.392291	0.711903	2.603298	H	0.032386	0.944765	2.584580	H	0.667842	0.006113	2.725978
	H	-0.614740	-0.886463	2.485009	H	-0.856912	-0.411284	2.582051	H	-0.773225	0.732069	2.589686
	H	2.516039	0.733031	0.804238	H	2.667996	0.451940	0.635918	H	0.017757	-2.736091	0.603711
	H	2.698082	-0.434534	-0.315463	H	2.480032	-1.152703	0.475431	H	-1.214490	-2.503488	-0.419238
	H	-1.218280	2.390933	0.496787	H	-0.531827	2.719643	-0.172257	H	2.696259	0.421782	0.616616
	H	0.384534	2.640034	0.557196	H	1.068417	2.573526	0.052558	H	2.483998	-1.179246	0.513769
	S=0				S=1				S=2			
$(H_3N)_5CrO^{2+}$	Cr	-0.001798	0.009177	-0.234735	Cr	0.002502	0.000106	-0.147019	Cr	-0.001028	-0.000058	0.000634
	O	-0.012988	0.068061	-1.753797	O	0.076470	0.001034	-1.758605	O	0.054450	0.060293	-1.872296
	N	-0.603381	2.014571	0.055176	N	2.121514	-0.011694	-0.049299	N	-0.274878	2.074369	-0.225018
	N	2.012329	0.634256	-0.055666	N	0.033658	-2.109655	-0.057140	N	2.098297	0.291778	0.009488
	N	0.025208	-0.137169	2.067476	N	-0.161190	-0.000963	2.113384	N	-0.075939	-0.081803	2.158040
	N	0.609400	-2.011336	-0.129831	N	-2.116356	0.011931	-0.262450	N	0.308806	-2.052900	-0.335534
	N	-2.029061	-0.576434	-0.075707	N	0.057228	2.109339	-0.054284	N	-2.097649	-0.279423	-0.125469
	H	0.051482	2.620352	0.549436	H	2.559578	-0.826079	0.381707	H	0.504938	2.664744	0.066079
	H	-1.507097	2.158585	0.505453	H	2.568283	0.796340	0.384709	H	-1.104664	2.488799	0.200503
	H	2.704509	-0.112466	0.004891	H	-0.866990	-2.530138	-0.289312	H	2.668511	-0.516402	0.260509
	H	2.235381	1.279717	0.701686	H	0.337240	-2.549371	0.811570	H	2.473685	1.065005	0.559222
	H	-0.577885	-0.877384	2.427480	H	-0.659923	0.808306	2.484488	H	-0.631523	-0.860401	2.514630
	H	-0.274227	0.705396	2.558980	H	0.732537	-0.008504	2.605942	H	-0.486107	0.751826	2.581375
	H	0.755267	-2.312780	-1.095176	H	-2.306179	0.012627	-1.267416	H	0.669269	-2.119517	-1.292565
	H	-0.061111	-2.671106	0.263886	H	-2.598172	0.823798	0.123972	H	-0.532726	-2.629976	-0.326453
	H	-2.482654	-0.270982	-0.938599	H	0.692471	2.432657	-0.786296	H	-2.268799	-0.331997	-1.133028
	H	-2.573694	-0.172390	0.685872	H	0.365211	2.544945	0.814940	H	-2.687267	0.476255	0.224056
	H	-0.697386	2.418569	-0.878620	H	2.413905	-0.011550	-1.029338	H	-0.371827	2.222061	-1.235004
	H	2.235348	1.129393	-0.920957	H	0.664708	-2.439639	-0.789802	H	2.315180	0.492341	-0.970185
	H	0.947909	-0.347273	2.449120	H	-0.672602	-0.803091	2.482736	H	0.838146	-0.181614	2.600838
	H	1.487887	-2.198198	0.353174	H	-2.607430	-0.794006	0.124722	H	0.980613	-2.536969	0.261146
	H	-2.198147	-1.581380	-0.032749	H	-0.838416	2.540179	-0.286793	H	-2.488826	-1.129266	0.281463
	S=1/2				S=3/2				S=5/2			
$(H_3N)_5MnO^{2+}$	Mn	-0.002819	-0.006763	-0.169571	Mn	0.003807	0.001326	-0.110004	Mn	0.000724	0.000043	0.007257
	O	-0.133526	-0.071019	-1.719675	O	0.070301	0.019134	-1.728836	O	-0.055273	0.000121	-1.801928
	N	-0.256905	2.036415	-0.050626	N	-0.261408	2.047740	-0.153738	N	-2.280207	-0.000360	0.123796
	N	-2.071826	-0.260421	-0.056105	N	-2.043456	-0.291710	-0.180021	N	-0.029492	-2.110413	-0.402922
	N	0.144784	0.104269	2.050317	N	-0.128822	-0.025972	2.102832	N	0.096875	-0.000276	2.102611

	N 0.249732 -2.040597 0.074257	N 0.323325 -2.037655 -0.167913	N 2.283933 0.000363 -0.010688
	N 2.044200 0.230033 -0.302488	N 2.048063 0.289861 0.005680	N -0.030248 2.110504 -0.402539
	H -1.185372 2.336507 0.247447	H -1.115293 2.262492 -0.671128	H -2.733528 -0.810350 0.546753
	H -2.555470 -0.147100 0.834770	H -2.392434 -1.136468 0.273794	H 0.828956 -2.398579 -0.874056
	H 0.029806 -0.789441 2.528877	H -0.467849 -0.911709 2.479255	H 0.593854 0.811262 2.473406
	H 0.232099 -2.463580 -0.855606	H 1.064030 -2.189723 -0.855280	H 2.515562 0.000576 -1.005440
	H 2.284571 -0.111517 -1.235404	H 2.365281 0.368754 -0.963249	H 0.828587 2.399507 -0.872452
	H 0.402279 2.532800 0.548989	H -0.284013 2.593697 0.707749	H -2.733711 0.809490 0.546819
	H -0.132581 2.415504 -0.991264	H 0.478802 2.434219 -0.742300	H -2.571126 -0.000344 -0.855125
	H -2.498612 0.395094 -0.712194	H -2.612287 0.468700 0.193695	H -0.168070 -2.756248 0.375104
	H -2.341011 -1.177272 -0.414132	H -2.268919 -0.368511 -1.174511	H -0.776886 -2.296596 -1.074157
	H 1.037039 0.468538 2.385880	H 0.756458 0.140616 2.582001	H -0.817685 -0.000662 2.555685
	H -0.560983 0.713023 2.465936	H -0.767067 0.675343 2.479631	H 0.594335 -0.811714 2.472974
	H 1.132430 -2.328921 0.496473	H 0.575632 -2.548999 0.678058	H 2.762473 0.809007 0.386034
	H -0.482264 -2.507236 0.610217	H -0.484839 -2.513007 -0.572131	H 2.762504 -0.808441 0.385675
	H 2.376066 1.194421 -0.277464	H 2.352577 1.135496 0.489213	H -0.776951 2.296420 -1.074620
	H 2.630804 -0.281471 0.356661	H 2.578432 -0.472975 0.428120	H -0.170249 2.755911 0.375590
	S=0	S=1	S=2
$(H_3N)_5FeO^{2+}$	Fe -0.006366 -0.000020 -0.113605	Fe 0.006181 -0.000035 -0.113243	Fe -0.004305 0.000244 -0.106937
	O -0.056152 0.006423 -1.716833	O 0.056502 -0.007393 -1.714125	O 0.037896 -0.000823 -1.700846
	N -1.438801 -1.435767 -0.062920	N 1.404425 1.468333 -0.066355	N 2.184191 -0.014182 0.003244
	N -1.448329 1.426579 -0.061086	N 1.479679 -1.392579 -0.058009	N 0.032088 -2.158328 -0.148238
	N 0.092021 -0.013451 2.035292	N -0.091970 0.013920 2.031718	N -0.108148 0.001780 2.003432
	N 1.418012 1.442805 -0.142878	N -1.383097 -1.475252 -0.143033	N -2.186852 0.013301 -0.125145
	N 1.434290 -1.425189 -0.166767	N -1.466054 1.391499 -0.166233	N 0.060079 2.157688 -0.149646
	H -2.378296 -1.103581 0.155490	H 2.354585 1.154902 0.132703	H 2.603296 -0.829102 0.451323
	H -1.275882 -2.228788 0.557701	H 1.233843 2.249806 0.566784	H 2.614754 0.795983 0.449069
	H -1.102119 2.382821 0.019456	H 1.156000 -2.356201 0.028972	H -0.839139 -2.521810 -0.535862
	H -2.172728 1.330907 0.650831	H 2.202192 -1.276229 0.652817	H 0.218433 -2.694327 0.698726
	H 1.027345 0.180804 2.394387	H -1.024535 -0.194322 2.390083	H -0.596063 0.817119 2.375056
	H -0.170201 -0.905366 2.455846	H 0.156250 0.910530 2.450652	H 0.803482 -0.005738 2.461333
	H 1.344159 1.893868 -1.056942	H -1.280586 -1.943927 -1.045259	H -2.463738 0.017551 -1.107911
	H 2.376722 1.098106 -0.087047	H -2.349987 -1.150636 -0.115881	H -2.634289 0.825133 0.300480
	H 1.074236 -2.379204 -0.196263	H -1.130871 2.354993 -0.173678	H 0.770475 2.416052 -0.835756
	H 2.136574 -1.412789 0.572969	H -2.178412 1.348815 0.562720	H 0.259480 2.691127 0.695961
	H -1.490593 -1.807123 -1.013786	H 1.433784 1.853601 -1.012489	H 2.519343 -0.017835 -0.961113
	H -1.913487 1.380833 -0.969977	H 1.944495 -1.342866 -0.966724	H 0.743725 -2.426394 -0.829317
	H -0.509089 0.676769 2.486289	H 0.519859 -0.666367 2.483371	H -0.609513 -0.805298 2.375188
	H 1.343320 2.176171 0.562419	H -1.308281 -2.192485 0.578703	H -2.642015 -0.796867 0.295424
	H 1.924413 -1.299124 -1.054464	H -1.941946 1.268997 -1.062074	H -0.808980 2.532835 -0.530988
$(H_3N)_5FeO^{2+}$	S=3		
	Fe 0.003365 -0.004705 -0.026336		
	O 0.072739 -0.162423 -1.896700		
	N 2.195509 0.061440 0.187040		
	N 0.098296 -2.183297 -0.234996		
	N -0.111357 0.254519 2.188574		
	N -2.199808 -0.096481 0.029731		
	N -0.045434 2.103260 -0.621895		
	H 2.613397 -0.669373 0.763247		
	H 2.580827 0.938474 0.538028		
	H -0.715117 -2.523744 -0.749084		
	H 0.171864 -2.759161 0.604225		
	H -0.809238 0.941894 2.474301		
	H 0.759313 0.554153 2.627120		
	H -2.505960 -0.257690 -0.931147		
	H -2.672611 0.754857 0.333651		
	H 0.768268 2.307923 -1.203627		
	H -0.072221 2.820925 0.102912		
	H 2.582911 -0.058736 -0.750200		
	H 0.898228 -2.424096 -0.822250		
	H -0.373284 -0.605495 2.672273		
	H -2.607506 -0.846133 0.588623		
	H -0.848719 2.271843 -1.228922		
	S=1/2	S=3/2	S=5/2
$(H_3N)_5CoO^{2+}$	Co 0.001368 0.002373 -0.022375	Co 0.003337 0.000000 -0.094906	Co -0.001706 -0.000752 -0.122117
	O 0.047512 0.064876 -1.789348	O 0.037695 -0.000323 -1.737546	O -0.041064 0.043343 -1.876443
	N -1.415093 1.379493 -0.149977	N -2.257799 0.000142 -0.045320	N -1.756179 -1.286334 -0.148052
	N -1.395966 -1.385238 -0.204461	N 0.033869 -2.001623 -0.202716	N 1.333170 -1.717062 -0.122982
	N -0.064863 -0.083445 2.023761	N -0.101258 0.000526 1.963081	N 0.069691 -0.112246 2.098483
	N 1.428980 -1.361130 -0.173163	N 2.254179 -0.000288 0.073117	N 1.699533 1.356978 -0.140369
	N 1.406970 1.394709 -0.038654	N 0.034388 2.001499 -0.203448	N -1.311318 1.728440 0.049540
	H -1.878046 1.226222 -1.049537	H -2.716519 -0.808047 0.374120	H -1.558244 -2.167394 -0.623171
	H -1.212994 -2.290322 0.229104	H 0.255637 -2.503500 0.656708	H 2.157147 -1.611268 0.468194
	H 0.300757 -0.957850 2.401976	H 0.812340 0.001604 2.417279	H 0.992600 0.088727 2.482689
	H 1.916894 -1.161695 -1.050330	H 2.585248 -0.000769 -0.892185	H 1.552552 2.068490 -0.857307
	H 1.546244 1.582939 -1.035843	H 0.728930 2.281177 -0.897942	H -1.594168 2.055913 -0.874786
	H -2.138688 1.367530 0.569273	H -2.716435 0.808694 0.373492	H -2.185147 -1.530702 0.743483
	H -1.073134 2.340066 -0.185223	H -2.536916 -0.000225 -1.026968	H -2.489075 -0.852032 -0.710041
	H -2.329058 -1.117302 0.109165	H -0.855824 -2.366963 -0.543364	H 0.891304 -2.584217 0.181795
	H -1.459846 -1.533434 -1.215855	H 0.728680 -2.281770 -0.896754	H 1.678251 -1.898572 -1.066206

	H	0.467745	0.653956	2.485807	H	-0.600523	0.809551	2.333178	H	-0.563442	0.530629	2.573370
	H	-1.008899	-0.008835	2.403621	H	-0.598904	-0.809196	2.333826	H	-0.169717	-1.037235	2.454766
	H	2.131033	-1.370607	0.567340	H	2.688913	0.810411	0.512878	H	1.911212	1.856933	0.722164
	H	1.099336	-2.322658	-0.261420	H	2.688631	-0.810758	0.513581	H	2.562627	0.887058	-0.414191
	H	1.187658	2.283217	0.412166	H	-0.855363	2.367028	-0.543726	H	-2.175030	1.549217	0.561128
	H	2.313773	1.114974	0.336114	H	0.256782	2.503558	0.655708	H	-0.880562	2.529572	0.510473
	S=0				S=1				S=2			
$(H_3N)_5NiO^{2+}$	Ni	0.004822	-0.000817	-0.003607	Ni	-0.001938	-0.003515	0.002801	Ni	-0.000508	0.001696	-0.095461
	O	-0.049197	-0.029798	-1.761679	O	-0.016607	0.086226	-1.755982	O	-0.004660	0.053011	-1.872011
	N	-2.133522	-0.138889	-0.010705	N	0.076506	1.996829	0.011456	N	0.013950	2.142476	0.021483
	N	-0.185242	2.019659	-0.234010	N	2.161183	-0.031259	-0.234539	N	2.147475	0.031256	-0.137869
	N	0.097721	0.021225	1.985825	N	0.007840	-0.136057	1.997964	N	-0.020720	-0.111184	2.063376
	N	2.129870	0.149724	-0.143317	N	-0.068492	-1.992351	-0.190694	N	0.011038	-2.142192	-0.126582
	N	0.118953	-2.026633	-0.201478	N	-2.159820	0.102641	-0.209836	N	-2.147344	0.035092	-0.155069
	H	-2.651532	0.649123	0.376928	H	0.919740	2.400266	0.419277	H	0.828484	2.512761	0.509514
	H	-2.550303	-0.972623	0.402895	H	-0.714802	2.467113	0.450802	H	-0.799422	2.529386	0.498536
	H	0.704985	2.502797	-0.351552	H	2.551978	-0.956493	-0.408896	H	2.553334	-0.872417	-0.378266
	H	-0.690022	2.508255	0.505191	H	2.727467	0.378669	0.507685	H	2.598410	0.324531	0.727565
	H	0.791772	-0.634427	2.345243	H	-0.742645	-0.731643	2.348551	H	-0.746279	-0.736930	2.412280
	H	-0.786515	-0.222959	2.431875	H	-0.101219	0.759651	2.473387	H	-0.185107	0.780604	2.529461
	H	2.283803	0.214800	-1.151011	H	-0.027355	-2.139308	-1.201932	H	0.182867	-2.502035	-1.065728
	H	2.668930	-0.652029	0.182705	H	-0.923599	-2.434443	0.146053	H	-0.867729	-2.560842	0.175414
	H	-0.661737	-2.347025	-0.775689	H	-2.391841	0.921929	-0.771555	H	-2.477405	0.827500	-0.705772
	H	0.112912	-2.589623	0.648739	H	-2.743939	0.148288	0.624568	H	-2.618017	0.098360	0.746421
	H	-2.350244	-0.166401	-1.008419	H	0.069295	2.246208	-0.980213	H	0.024564	2.568860	-0.905387
	H	-0.703405	2.178056	-1.100432	H	2.358383	0.504088	-1.081253	H	2.474671	0.672676	-0.860513
	H	0.362900	0.932843	2.359219	H	0.875370	-0.539332	2.352700	H	0.849287	-0.466044	2.459277
	H	2.574513	0.964491	0.278459	H	0.706105	-2.506456	0.228119	H	0.730838	-2.548026	0.469917
	H	0.958050	-2.279618	-0.723922	H	-2.496323	-0.688561	-0.758310	H	-2.527774	-0.788105	-0.621102
	S=1/2				S=3/2				S=5/2			
$(H_3N)_5CuO^{2+}$	Cu	-0.138844	0.001598	-0.004894	Cu	0.119154	-0.000029	0.004469	Cu	-0.002520	0.000017	-0.027873
	O	2.570929	-0.008830	0.053940	O	-2.421869	-0.001799	-0.070117	O	-0.249502	0.017583	-2.055600
	N	0.368740	-1.352492	-1.529018	N	-0.368074	-2.051991	0.054641	N	-0.058205	-2.088291	-0.182899
	N	0.029779	-1.556447	1.336738	N	0.009221	-0.050176	-2.049447	N	2.062589	0.035151	-0.311623
	N	-2.383991	-0.015200	-0.036062	N	2.361442	0.001810	0.030645	N	0.379935	-0.018052	1.984789
	N	0.290467	1.365021	1.535399	N	-0.369401	2.051675	-0.045714	N	-0.118717	2.089015	-0.144186
	N	0.081784	1.561793	-1.336250	N	-0.083867	0.050200	2.051721	N	-2.072692	-0.030927	0.249097
	H	1.132442	-1.967759	-1.247271	H	-0.834581	-2.381092	-0.790099	H	0.858022	-2.493313	-0.405706
	H	-0.459189	-1.388646	2.215370	H	0.449536	0.750980	-2.500628	H	2.442120	0.861175	0.172672
	H	-2.800826	0.259562	0.852473	H	2.769715	0.806475	-0.443329	H	0.908491	0.844023	2.232861
	H	1.116281	1.923722	1.317639	H	-1.002558	2.324593	0.705804	H	-0.818446	2.430586	-0.813108
	H	1.069988	1.752005	-1.510347	H	-1.072646	0.057373	2.307294	H	-2.640160	-0.025918	-0.606131
	H	-0.411822	-1.959664	-1.778643	H	0.456198	-2.641239	0.171999	H	-0.329253	-2.446086	0.743293
	H	0.674379	-0.923528	-2.401687	H	-0.998413	-2.289215	0.820453	H	-0.737032	-2.437507	-0.869184
	H	-0.336737	-2.439253	0.982284	H	0.446372	-0.873991	-2.461161	H	2.468440	-0.791122	0.150468
	H	1.007856	-1.728079	1.575259	H	-0.966609	-0.055710	-2.350689	H	2.388099	0.053256	-1.284139
	H	-2.795508	0.611015	-0.726667	H	2.770598	0.008956	0.963937	H	-0.504326	-0.047848	2.524316
	H	-2.782036	-0.930682	-0.241157	H	2.769484	-0.810186	-0.430873	H	0.951314	-0.859100	2.209123
	H	-0.471137	2.026993	1.683644	H	0.454017	2.646524	0.046567	H	-0.385324	2.421754	0.792606
	H	0.476007	0.949065	2.447212	H	-0.832948	2.339829	-0.906854	H	0.781433	2.524778	-0.373953
	H	-0.344011	1.383213	-2.245211	H	0.332153	-0.752695	2.522631	H	-2.322170	-0.865238	0.795156
	H	-0.324095	2.437596	-1.008148	H	0.333933	0.874000	2.483357	H	-2.342491	0.781120	0.818603

Table S3. Optimized geometries (Cartesian coordinates in Å) for the encounter complex between methane and $(NH_3)_5MO^{2+}$ species.

	S=0				S=1							
$(H_3N)_5TiO^{2+}$	Ti	-0.382191	0.003558	-0.188626	Ti	-0.453968	0.011638	-0.018551				
	O	0.295656	-0.000188	-1.616552	O	0.495868	0.275377	-1.575073				
	N	-2.000396	1.430134	-0.770474	N	0.994808	1.532616	0.741154				
	N	-1.837666	-1.642505	-0.625240	N	-1.723275	-0.425276	1.858360				
	N	1.009573	-1.526667	0.619972	N	0.889598	-1.621003	0.735560				
	N	-1.365693	0.060203	2.080239	N	-1.525075	-1.404478	-1.393578				
	N	0.885299	1.677577	0.536714	N	-1.945370	1.637129	-0.471714				
	C	3.940543	-0.005540	-0.211300	C	3.920147	0.042249	-0.198602				
	H	0.681448	2.159471	1.411372	H	4.772457	-0.527796	-0.556757				
	H	-0.667214	-0.008685	2.820561	H	4.213918	1.087131	-0.120221				
	H	0.859848	-1.953220	1.533492	H	3.647361	-0.337231	0.788835				
	H	-1.718316	-2.497145	-0.080510	H	3.104831	-0.058882	-0.918218				
	H	-1.693933	2.403971	-0.746790	H	0.975748	2.363686	0.148093				
	H	-2.890062	1.412514	-0.272614	H	0.906472	1.874098	1.699127				
	H	-2.211123	1.253256	-1.754787	H	-1.898836	-1.421328	1.997800				
	H	-2.828112	-1.411730	-0.543748	H	-2.644106	0.014141	1.863111				
	H	-1.691977	-1.911393	-1.600349	H	1.665116	-1.625254	0.067717				
	H	1.064304	-2.290997	-0.056142	H	0.528414	-2.574950	0.747167				
	H	1.959992	-1.143338	0.629064	H	-1.789381	-0.913312	-2.249174				
	H	-1.877842	0.917392	2.288758	H	-2.374935	-1.862657	-1.062337				
	H	-2.026916	-0.691044	2.277178	H	-2.907910	1.356147	-0.659981				
	H	0.921906	2.392023	-0.192968	H	-2.014156	2.410945	0.189746				
	H	1.853639	1.350584	0.603457	H	-1.609841	2.051954	-1.343763				
	H	3.099642	0.170029	-0.886035	H	-0.906519	-2.153781	-1.706346				
	H	3.719420	0.280081	0.821228	H	1.324552	-1.483616	1.648342				
	H	4.784317	0.589361	-0.548406	H	-1.277557	-0.115801	2.723978				
	H	4.222845	-1.055865	-0.241268	H	1.949042	1.171053	0.654717				
	S=1/2				S=3/2							
$(H_3N)_5VO^{2+}$	V	-0.361689	0.002510	-0.178148	V	0.452390	0.000159	0.028247				
	O	0.383627	0.002571	-1.534821	O	-0.416329	-0.081290	1.641322				
	N	-1.729238	1.605258	-0.706142	N	1.387353	1.709401	0.988888				
	N	-1.608136	-0.019073	1.850979	N	2.087198	-1.281404	0.678524				
	N	0.961954	1.451445	0.731376	N	-0.785873	-1.729690	-0.359050				
	N	0.834378	-1.542483	0.750613	N	1.514584	0.097019	-1.936242				
	N	-1.790152	-1.492937	-0.848593	N	-1.027084	1.296560	-0.887332				
	C	3.895646	-0.009863	-0.229123	C	-3.893885	-0.030115	0.171348				
	H	4.158259	1.045680	-0.188826	H	-1.643779	0.849248	-1.567202				
	H	3.639458	-0.363429	0.772259	H	0.880358	0.141902	-2.734378				
	H	4.764048	-0.565300	-0.571140	H	-1.770334	-1.449914	-0.336850				
	H	3.083181	-0.161838	-0.942909	H	2.095314	-2.224210	0.288055				
	H	-1.382211	2.017798	-1.574034	H	1.020430	1.735317	1.944762				
	H	-2.690349	1.325748	-0.903205	H	1.171822	2.624300	0.592185				
	H	-1.361131	0.715610	2.514058	H	2.402751	1.689017	1.085848				
	H	-1.521401	-0.887541	2.379132	H	3.036638	-0.927985	0.558214				
	H	1.919643	1.137042	0.545606	H	1.958647	-1.403567	1.685543				
	H	0.908836	1.634414	1.732698	H	-0.656443	-2.263280	-1.218860				
	H	1.683301	-1.624211	0.185695	H	-0.693244	-2.397353	0.408747				
	H	0.426064	-2.476954	0.778414	H	2.125344	0.909198	-2.032007				
	H	-1.272404	-2.278953	-1.244866	H	2.111488	-0.709818	-2.120063				
	H	-2.455010	-1.891270	-0.186518	H	-0.702672	2.157107	-1.328752				
	H	-2.343166	-1.140624	-1.631061	H	-1.646288	1.578178	-0.122741				
	H	1.151138	-1.350935	1.701256	H	-3.065064	-0.115882	0.877842				
	H	0.897854	2.361969	0.274584	H	-4.131804	1.018230	0.002240				
	H	-2.609906	0.097718	1.697616	H	-4.763391	-0.517847	0.601597				
	H	-1.801896	2.370492	-0.035676	H	-3.674060	-0.508484	-0.786036				
	S=0				S=1				S=2			
$(H_3N)_5CrO^{2+}$	Cr	0.371960	-0.002696	-0.183217	Cr	-0.399731	-0.000641	0.101774	Cr	-0.471589	-0.006439	0.017734
	O	-0.330498	-0.020936	-1.532852	O	0.411150	0.007396	1.497761	O	0.490380	0.134485	-1.587174
	N	-0.817700	-1.528724	0.660564	N	-1.595097	-1.597940	0.828556	N	-1.709840	1.577798	-0.654073
	N	1.822607	-1.455161	-0.699373	N	-1.787970	1.429536	0.807249	N	-1.601971	-1.368977	-1.122720
	N	1.736061	1.529652	-0.696869	N	0.809060	1.578784	-0.622118	N	0.813786	-1.578347	0.607694
	N	1.423020	0.052972	1.872624	N	-1.592115	-0.044808	-1.825419	N	-1.578173	-0.167062	1.867237
	N	-0.933297	1.425318	0.653106	N	0.943824	-1.352201	-0.785392	N	0.939503	1.400733	0.667277
	C	-3.895445	0.000953	-0.216234	C	3.876726	-0.018672	0.213985	C	3.874493	0.045046	-0.150729
	H	-0.775296	2.377720	0.324084	H	0.897111	-1.501835	-1.792813	H	0.718795	2.005274	1.459165
	H	1.743719	0.985550	2.134260	H	-1.279041	0.619410	-2.533918	H	-0.985736	-0.341084	2.679920
	H	1.649011	2.402694	-0.177128	H	1.314759	1.407903	-1.491601	H	1.341862	-1.441281	1.470012
	H	2.802559	-1.187431	-0.611480	H	-2.267101	2.010446	0.119585	H	-1.739925	-2.304209	-0.738694
	H	-1.203324	-2.060510	-0.121900	H	-1.236992	-1.760580	1.772431	H	-1.414812	1.720900	-1.623196
	H	-1.634356	-1.189121	1.170248	H	-1.519070	-2.486156	0.332835	H	-1.578612	2.475084	-0.186141
	H	-0.362048	-2.208300	1.268977	H	-2.597069	-1.431682	0.921762	H	-2.718840	1.428270	-0.671076
	H	1.739738	-2.357442	-0.231345	H	-2.517170	1.027844	1.397482	H	-2.527050	-1.053113	-1.415126
	H	1.680013	-1.649752	-1.692090	H	-1.277748	2.063656	1.424722	H	-1.065932	-1.493696	-1.987669
	H	2.727588	1.293166	-0.670927	H	0.359924	2.487253	-0.738214	H	0.410044	-2.512103	0.682470

	H 1.535634 1.768074 -1.669868	H 1.520197 1.695658 0.104171	H 1.505917 -1.618552 -0.145570
	H 0.827416 -0.244940 2.645717	H -1.576031 -0.953680 -2.288861	H -2.114613 0.669033 2.100933
	H 2.252600 -0.536738 1.942293	H -2.584208 0.155493 -1.696103	H -2.258966 -0.927830 1.867393
	H -0.987304 1.484352 1.669384	H 0.896773 -2.275634 -0.353223	H 1.126762 2.013099 -0.131229
	H -1.876071 1.185900 0.331414	H 1.892005 -1.020121 -0.577896	H 1.846475 0.970885 0.868494
	H -3.053115 -0.301814 -0.841917	H 3.074969 0.091008 0.947385	H 3.074748 -0.041077 -0.889328
	H -3.735407 -0.243683 0.835613	H 4.109201 -1.071915 0.065677	H 4.122776 1.092453 0.009342
	H -4.780577 -0.529026 -0.555742	H 4.764385 0.473519 0.600853	H 4.755725 -0.462091 -0.532608
	H -4.075993 1.069369 -0.322540	H 3.625172 0.444074 -0.742970	H 3.606385 -0.420595 0.801148
	S=1/2	S=3/2	S=5/2
$(H_3N)_5MnO^{2+}$	Mn 0.385647 0.003874 -0.125117	Mn 0.414222 -0.000944 -0.076129	Mn 0.472535 0.032285 0.030031
	O -0.435542 0.089556 -1.446469	O -0.321700 -0.020546 -1.520755	O -0.433465 -0.452767 -1.463653
	N 1.445512 -1.613256 -0.848024	N -0.926615 -1.413500 0.583748	N -0.902491 -1.369428 0.849558
	N 1.849518 1.326367 -0.731951	N 1.384859 0.039745 1.915317	N 1.511446 0.615030 1.758241
	N -0.737875 1.606311 0.590259	N -0.816939 1.543457 0.504619	N -1.001269 1.733658 0.454358
	N 1.604994 -0.117196 1.737339	N 1.788096 1.372512 -0.794339	N 1.469419 1.210498 -1.449617
	N -0.927476 -1.250259 0.837487	N 1.682685 -1.523737 -0.680330	N 2.007399 -1.650347 -0.298541
	C -3.880022 -0.033554 -0.187672	C -3.905011 -0.000835 -0.165906	C -3.914963 -0.261740 -0.229483
	H -1.158838 -0.977411 1.792851	H -3.679353 0.273205 0.868931	H -3.673254 0.274276 0.691232
	H 1.529557 0.704315 2.337783	H -4.768127 0.578574 -0.480676	H -4.818740 0.174876 -0.644571
	H -1.723119 1.335087 0.541622	H -4.161756 -1.057236 -0.211658	H -4.110873 -1.308859 -0.006923
	H 1.924802 2.168055 -0.160630	H -3.079504 0.211333 -0.849614	H -3.117684 -0.180905 -0.971261
	H 0.797783 -2.106962 -1.464874	H -1.874891 -1.040609 0.473570	H -1.857673 -1.028397 0.701931
	H 1.802699 -2.297181 -0.181272	H -0.899964 -2.199289 -0.067782	H -0.844467 -2.240192 0.319003
	H 2.233228 -1.363971 -1.446318	H 1.987026 0.849477 2.065826	H 1.831773 1.583698 1.715695
	H 2.795046 0.950223 -0.800196	H 1.976220 -0.767913 2.112186	H 2.340691 0.050930 1.946747
	H 1.603341 1.640402 -1.672349	H -0.973444 2.105285 -0.334611	H -1.700986 1.621489 -0.281734
	H -0.581571 1.983459 1.524688	H -0.518043 2.167871 1.253637	H -0.669086 2.695323 0.385619
	H -0.660557 2.389404 -0.059342	H 1.761193 1.262881 -1.810371	H 1.724943 0.602570 -2.229865
	H 1.371818 -0.906170 2.340679	H 2.760214 1.283136 -0.498200	H 2.314753 1.716206 -1.182935
	H 2.603315 -0.217467 1.552215	H 2.670680 -1.280997 -0.756581	H 2.974306 -1.388283 -0.489773
	H -0.657686 -2.233143 0.877085	H 1.652820 -2.379717 -0.125739	H 2.065343 -2.382229 0.409406
	H -1.808900 -1.216399 0.316256	H 1.367798 -1.765214 -1.622631	H 1.673121 -2.111391 -1.146085
	H -3.072756 0.200891 -0.885396	H 1.533111 2.344857 -0.618426	H 0.826488 1.897584 -1.845324
	H -4.041407 -1.109760 -0.142927	H -1.749086 1.204733 0.754534	H -1.520854 1.693160 1.331081
	H -4.789781 0.431123 -0.556486	H 0.712319 0.067739 2.682263	H 0.935746 0.559655 2.599786
	H -3.686378 0.349748 0.816544	H -0.853711 -1.794483 1.526944	H -0.820952 -1.619930 1.835320
	S=0	S=1	S=2
$(H_3N)_5FeO^{2+}$	Fe 0.409482 -0.002479 -0.077247	Fe 0.410539 -0.001744 -0.077513	Fe 0.417333 0.001655 -0.056652
	O -0.356249 -0.020515 -1.489353	O -0.351063 -0.007243 -1.487354	O -0.359860 0.041425 -1.449309
	N 1.638065 -1.495711 -0.696894	N 1.639372 -1.489987 -0.706305	N -0.909513 -1.564884 0.707316
	N 1.728686 1.366600 -0.794769	N 1.730974 1.371170 -0.782715	N 1.485255 -0.019796 1.766100
	N -0.788237 1.497694 0.557606	N -0.788148 1.492719 0.566648	N -0.965495 1.453497 0.705622
	N 1.408600 0.023983 1.826483	N 1.406057 0.007172 1.824788	N 1.784715 1.573000 -0.728764
	N -0.898890 -1.371988 0.625766	N -0.900332 -1.372094 0.616697	N 1.703250 -1.529549 -0.865180
	C -3.889641 0.000436 -0.181735	C -3.896249 -0.000182 -0.184835	C -3.911511 0.052271 -0.325131
	H -0.897434 -1.582644 1.623564	H -0.934217 -1.545122 1.621192	H -3.684022 -0.336258 0.669967
	H 0.767162 0.157068 2.608960	H 0.764454 0.144321 2.606441	H -4.154570 1.111033 -0.260232
	H -1.665712 1.169136 0.964135	H -1.674731 1.158553 0.948508	H -4.781536 -0.476944 -0.702822
	H 1.524198 2.332735 -0.538868	H 1.518337 2.336820 -0.531726	H -3.084797 -0.096805 -1.023012
	H 1.272337 -1.792484 -1.603950	H 1.273827 -1.785881 -1.613539	H -1.651728 -1.660555 0.012020
	H 1.667048 -2.326452 -0.105557	H 1.673209 -2.322597 -0.117884	H -0.498072 -2.491430 0.818452
	H 2.611165 -1.234521 -0.855166	H 2.611231 -1.225127 -0.866483	H 1.470089 0.881355 2.243967
	H 2.713938 1.222610 -0.573618	H 2.714206 1.233136 -0.548821	H 2.472731 -0.244378 1.642974
	H 1.642597 1.327283 -1.812205	H 1.659134 1.329385 -1.801049	H -0.910640 2.307154 0.149317
	H -0.394083 2.169263 1.216375	H -0.403295 2.146860 1.248090	H -0.949890 1.738219 1.684114
	H -1.066808 2.015023 -0.278385	H -1.050327 2.032338 -0.260269	H 1.531700 1.772989 -1.697338
	H 1.914032 -0.835869 2.041395	H 1.901301 -0.859145 2.037345	H 2.779016 1.346304 -0.732754
	H 2.100052 0.767887 1.924204	H 2.104936 0.743568 1.926338	H 2.352402 -1.140128 -1.549298
	H -0.794251 -2.261586 0.137505	H -0.769806 -2.279417 0.169258	H 2.256640 -2.119506 -0.244750
	H -1.843147 -1.057112 0.382360	H -1.837809 -1.074932 0.328846	H 1.101438 -2.151054 -1.407045
	H -3.061662 0.265395 -0.843567	H -3.072292 0.277598 -0.846305	H 1.710848 2.458728 -0.228445
	H -4.073795 -1.071702 -0.222421	H -4.070779 -1.073697 -0.232799	H -1.901917 1.084185 0.516132
	H -4.780872 0.513799 -0.530972	H -4.792851 0.507292 -0.528848	H 1.130811 -0.696904 2.441737
	H -3.711024 0.304085 0.852537	H -3.718003 0.297568 0.851098	H -1.378689 -1.359952 1.589562
	S=3		
$(H_3N)_5FeO^{2+}$	Fe -0.448829 0.004298 0.001610		
	O 0.564654 0.343400 -1.544644		
	N -1.854869 1.655948 -0.402578		
	N -1.436287 -1.294205 -1.468318		
	N 0.868134 -1.612256 0.690185		
	N -1.773948 -0.514054 1.723530		
	N 0.975654 1.436730 0.817617		
	C 3.947576 0.049227 -0.158084		
	H 0.910845 1.701780 1.800730		
	H -1.526113 -0.010464 2.576344		
	H 1.160676 -1.575235 1.666670		
	H -0.738980 -1.909635 -1.889304		
	H -1.488125 2.152204 -1.216160		
	H -1.957256 2.352021 0.335926		

	H -2.805145 1.380263 -0.650549		
	H -2.206689 -1.889647 -1.164086		
	H -1.790283 -0.732529 -2.243535		
	H 0.523295 -2.560639 0.541399		
	H 1.722268 -1.530901 0.133996		
	H -2.758994 -0.301989 1.565364		
	H -1.744981 -1.501402 1.977953		
	H 0.942758 2.304079 0.280572		
	H 1.921173 1.070680 0.672199		
	H 3.150776 -0.066220 -0.896544		
	H 4.187953 1.103812 -0.037612		
	H 4.832030 -0.464960 -0.522767		
	H 3.680883 -0.380653 0.810154		
	S=1/2	S=3/2	S=5/2
$(H_3N)_5CoO^{2+}$	Co -0.443719 -0.000512 -0.006943	Co 0.412225 0.011794 -0.052538	Co -0.400577 -0.002353 -0.077042
	O 0.466118 -0.009189 -1.524216	O -0.365652 -0.420851 -1.437220	O 0.434987 0.014677 -1.619614
	N -1.436859 1.538334 -0.764668	N 1.922780 -1.660068 -0.239327	N -1.726206 1.620317 -0.686123
	N -1.687265 -1.284260 -0.855804	N 1.583097 1.165154 -1.199884	N -1.885552 -1.406539 -0.836291
	N 0.718875 -1.505924 0.517367	N -0.993284 1.728794 0.336120	N 0.856585 -1.635683 0.584297
	N -1.503346 -0.015042 1.746415	N 1.360019 0.592630 1.685097	N -1.418026 -0.054759 1.901033
	N 0.889834 1.283935 0.680282	N -0.836871 -1.223923 0.896416	N 0.991047 1.469427 0.689153
	C 3.848431 -0.008278 -0.113518	C -3.873716 -0.313086 -0.236977	C 3.950445 0.002294 -0.225256
	H 1.480141 1.478412 -0.134298	H -0.785884 -2.149525 0.469376	H 0.840881 1.828141 1.630784
	H -0.976702 0.340328 2.544673	H 0.745137 0.572688 2.498654	H -0.760199 -0.009818 2.678810
	H 1.697059 -1.202065 0.501777	H -1.537216 1.677452 1.196850	H 1.826012 -1.317384 0.644539
	H -1.742513 -2.201759 -0.413151	H 1.073722 1.980524 -1.541248	H -1.673698 -2.379466 -0.616194
	H -0.811521 1.946043 -1.464612	H 1.647955 -2.158907 -1.085978	H -1.497066 1.922814 -1.633389
	H -1.687062 2.283640 -0.114462	H 1.934870 -2.359076 0.502758	H -1.658965 2.452112 -0.100332
	H -2.293384 1.305247 -1.267203	H 2.898052 -1.397671 -0.378628	H -2.715766 1.376195 -0.703459
	H -2.653908 -0.983625 -0.979962	H 2.447173 1.504665 -0.778579	H -2.839825 -1.253768 -0.512576
	H -1.289340 -1.425642 -1.788847	H 1.847101 0.637418 -2.033226	H -1.916166 -1.358162 -1.855165
	H 0.557578 -1.958139 1.417404	H -0.619159 2.676711 0.310122	H 0.638068 -2.078798 1.475261
	H 0.671129 -2.229265 -0.201717	H -1.675184 1.670143 -0.421234	H 0.874109 -2.378662 -0.114780
	H -2.357097 0.542677 1.715450	H 2.162443 0.010207 1.924908	H -2.073782 0.709790 2.058669
	H -1.809195 -0.949248 2.019687	H 1.708699 1.550271 1.641122	H -1.956044 -0.907192 2.054392
	H 1.516656 0.935271 1.406750	H -1.800969 -0.908546 0.751402	H 1.016643 2.280401 0.070650
	H 0.530877 2.175814 1.020950	H -0.700339 -1.350995 1.898465	H 1.943299 1.097075 0.664088
	H 3.063855 -0.200306 -0.849673	H -3.082758 -0.228936 -0.985119	H 3.105032 -0.207941 -0.885005
	H 4.000863 1.063346 -0.001476	H -4.002660 -1.353237 0.056717	H 4.182250 1.064982 -0.249785
	H 4.769447 -0.454906 -0.476101	H -4.802806 0.033881 -0.679910	H 4.812349 -0.549965 -0.588016
	H 3.625340 -0.448124 0.861976	H -3.670938 0.301704 0.642740	H 3.760932 -0.307356 0.805603
	S=0	S=1	S=2
$(H_3N)_5NiO^{2+}$	Ni -0.449160 -0.020195 0.023694	Ni -0.452056 0.024303 0.025779	Ni 0.407668 -0.001977 -0.055336
	O 0.419742 0.322143 -1.467544	O 0.340089 -0.221503 -1.528340	O -0.491352 -0.018971 -1.588168
	N -1.731382 1.652242 -0.422604	N -1.759882 1.199181 -0.933562	N 1.709400 -1.561644 -0.750080
	N -1.623812 -1.171206 -1.173334	N -1.657893 -1.700052 -0.550490	N 1.776712 1.449181 -0.854627
	N 0.886484 -1.658256 0.358382	N 0.915978 -1.198143 0.809743	N -0.849453 1.579407 0.650752
	N -1.411934 -0.430718 1.719316	N -1.325449 0.294762 1.802974	N 1.454459 0.019275 1.836891
	N 0.898525 1.248787 0.839639	N 0.899602 1.699378 0.198300	N -0.958435 -1.467921 0.694522
	C 3.906764 0.161447 -0.231196	C 3.924617 -0.187220 -0.193835	C -3.921579 -0.000064 -0.233490
	H 0.807500 1.469738 1.830683	H 0.841652 2.314210 1.009170	H -0.871482 -1.699323 1.682687
	H -0.780626 -0.474496 2.519317	H -0.644744 0.488214 2.537740	H 0.816220 0.080615 2.629638
	H 1.508064 -1.572613 1.162031	H 1.646209 -0.729873 1.347793	H -1.729164 1.228216 1.029033
	H -1.204026 -2.078991 -1.371867	H -1.294456 -2.597844 -0.233039	H 1.544651 2.409096 -0.603094
	H -1.330976 2.007987 -1.292056	H -1.429595 1.187417 -1.901113	H 1.459741 -1.857080 -1.694086
	H -1.732191 2.428268 0.238767	H -1.780003 2.172326 -0.629272	H 1.678276 -2.402240 -0.174812
	H -2.712548 1.453047 -0.615118	H -2.726026 0.873274 -0.938400	H 2.689966 -1.288168 -0.793884
	H -2.574919 -1.346395 -0.850179	H -2.645149 -1.691606 -0.296433	H 2.746299 1.311900 -0.573097
	H -1.698861 -0.699651 -2.076409	H -1.621041 -1.739592 -1.570018	H 1.771613 1.422684 -1.874409
	H 0.492425 -2.596856 0.412299	H 0.565540 -1.963256 1.385185	H -0.437017 2.171385 1.370154
	H 1.484896 -1.628396 -0.469100	H 1.381962 -1.598168 -0.008167	H -1.117183 2.197038 -0.115771
	H -2.127277 0.257127 1.954624	H -1.986903 1.070696 1.822318	H 2.017534 -0.813552 2.006192
	H -1.881623 -1.335820 1.692381	H -1.845614 -0.527736 2.109117	H 2.094462 0.805426 1.945477
	H 0.868385 2.133299 0.331691	H 0.804141 2.292151 -0.626032	H -0.885707 -2.348232 0.185900
	H 1.843115 0.882155 0.688840	H 1.857851 1.346188 0.157664	H -1.919490 -1.151190 0.548829
	H 3.114146 0.084485 -0.978374	H 3.128458 -0.081307 -0.934171	H -3.063395 0.313373 -0.833111
	H 4.074990 1.203949 0.032647	H 4.857176 0.137368 -0.645494	H -4.095421 -1.066633 -0.361691
	H 4.822192 -0.234058 -0.661702	H 4.024057 -1.230400 0.099582	H -4.797271 0.537115 -0.585879
	H 3.680138 -0.419080 0.665825	H 3.749148 0.426904 0.692745	H -3.785807 0.229004 0.825397

Table S4. Optimized geometries (Cartesian coordinates in Å) for the transition states of the reaction between methane and $(NH_3)_5MO^{2+}$ species.

	S=0	S=1	
$(H_3N)_5TiO^{2+}$	Ti 0.279206 0.003606 -0.027727	Ti 0.325188 0.000628 -0.044742	
	O -1.181580 0.159074 -0.926121	O -0.936985 0.027125 -1.354435	
	N 1.281502 -0.916086 -1.828934	N 1.414868 -1.659463 -1.114305	
	N 1.030490 2.011504 -0.726942	N 1.523480 1.502560 -1.225962	
	N -0.605837 0.910995 1.832233	N -0.657386 1.688492 1.080654	
	N 2.365051 -0.159671 1.059058	N 1.948701 -0.015830 1.601421	
	N -0.202734 -2.049192 0.771146	N -0.797731 -1.542411 1.152955	
	C -3.649777 0.073019 -0.309899	C -3.447680 0.003654 -0.332505	
	H -0.161087 -2.192258 1.779711	H -1.352711 -1.199807 1.937065	
	H 2.475906 0.507695 1.822462	H 1.564907 0.025516 2.545993	
	H -0.325509 0.553431 2.744801	H -1.173046 1.447397 1.926857	
	H 1.199046 2.731592 -0.024966	H 1.597516 2.445413 -0.844287	
	H 0.496626 -1.338676 -2.328185	H 0.887267 -1.771142 -1.983273	
	H 1.984834 -1.646258 -1.723689	H 1.406777 -2.581488 -0.678485	
	H 1.675187 -0.249339 -2.492534	H 2.381755 -1.503463 -1.398413	
	H 1.855377 2.029041 -1.325968	H 2.468850 1.253070 -1.515705	
	H 0.260772 2.354000 -1.304613	H 0.988968 1.607743 -2.091684	
	H -0.512843 1.925127 1.894368	H -0.088263 2.496375 1.332802	
	H -1.612430 0.747039 1.762207	H -1.361022 2.037566 0.426285	
	H 2.540103 -1.073211 1.477253	H 2.538112 -0.848679 1.587242	
	H 3.160643 0.007897 0.443209	H 2.596571 0.770300 1.540826	
	H 0.342956 -2.810211 0.366218	H -0.283827 -2.345760 1.514508	
	H -1.168744 -2.242091 0.501193	H -1.478471 -1.920953 0.490765	
	H -2.371697 0.123916 -0.675394	H -2.453771 0.022539 -0.882698	
	H -3.745591 -0.715883 0.428400	H -3.296683 0.205469 0.726850	
	H -4.101862 -0.155847 -1.268545	H -3.879875 -0.980813 -0.489938	
	H -3.862207 1.071153 0.056490	H -4.058753 0.774544 -0.793216	
	S=1/2	S=3/2	
$(H_3N)_5VO^{2+}$	V 0.287845 -0.000038 -0.021490	V 0.348698 0.000011 -0.022377	
	O -1.133400 -0.001756 -0.977806	O -1.114301 0.000822 -1.098033	
	N 1.334817 -0.002171 -1.954810	N 1.330929 0.001032 -1.964920	
	N 0.449571 2.173662 0.081027	N 0.493515 2.172447 0.043947	
	N -0.831961 0.001810 1.840849	N -0.927239 -0.000848 1.746408	
	N 2.304819 0.002267 1.033408	N 2.212504 -0.000840 1.227299	
	N 0.451995 -2.173261 0.086295	N 0.493242 -2.172522 0.041543	
	C -3.587869 -0.000858 -0.256444	C -3.576113 0.000054 -0.175956	
	H 0.634070 -2.589705 0.998992	H 0.265647 -2.623522 0.927635	
	H 2.882738 -0.805513 0.802001	H 2.289893 -0.810797 1.842590	
	H -0.719990 -0.808506 2.449702	H -1.555452 -0.804939 1.719694	
	H 0.629784 2.592522 0.992988	H 0.265997 2.622554 0.930513	
	H 1.001994 -0.804327 -2.490488	H 0.538454 0.001567 -2.614171	
	H 2.350871 -0.003827 -2.032673	H 1.897165 -0.809658 -2.213886	
	H 1.004719 0.800335 -2.491634	H 1.897497 0.811784 -2.212929	
	H 1.141660 2.580945 -0.547984	H 1.377717 2.585202 -0.252888	
	H -0.450027 2.543853 -0.229279	H -0.208770 2.500558 -0.622275	
	H -0.716658 0.810991 2.450581	H -0.501321 -0.001344 2.673011	
	H -1.817109 0.003897 1.560534	H -1.555313 0.803376 1.720502	
	H 2.880341 0.812083 0.803149	H 3.069110 -0.000925 0.672769	
	H 2.237355 0.001448 2.051221	H 2.290354 0.808739 1.843030	
	H 1.143628 -2.581249 -0.542762	H 1.377414 -2.585059 -0.255684	
	H -0.447621 -2.545241 -0.221822	H -0.209052 -2.499875 -0.625040	
	H -2.246774 -0.001384 -0.638645	H -2.473027 0.000177 -0.566030	
	H -3.998406 -0.003239 -1.259164	H -4.172492 0.000396 -1.082816	
	H -3.750745 0.918108 0.294493	H -3.731701 0.904748 0.405658	
	H -3.750535 -0.917269 0.298791	H -3.731747 -0.905030 0.405042	
	S=0	S=1	S=2
$(H_3N)_5CrO^{2+}$	Cr 0.310764 0.000002 -0.002275	Cr 0.325663 -0.006501 -0.005589	Cr 0.371635 0.001060 0.017716
	O -1.103512 0.000077 -0.978491	O -1.052939 0.944994 0.481648	O -1.023199 0.019729 -1.215297
	N 0.354863 -2.130695 -0.011306	N 1.065081 1.787835 -0.901898	N 1.149945 -1.587525 -1.141983
	N 1.443852 0.000284 -1.809231	N 1.195806 0.607897 1.845412	N 1.281609 1.351685 -1.328016
	N 0.354740 2.130693 -0.010723	N -0.434525 -1.762823 0.889239	N -0.519410 1.642272 1.012096
	N 2.221886 -0.000077 1.094305	N 2.211137 -0.994134 -0.542680	N 2.038102 -0.010556 1.408106
	N -0.810312 -0.000323 1.784259	N -0.613195 -0.646901 -1.780244	N -0.699817 -1.417911 1.161996
	C -3.531634 0.000053 -0.252378	C -3.455728 0.264148 0.112721	C -3.363176 0.003495 -0.172785
	H -0.693844 0.809866 2.392456	H -0.423528 -1.585793 -2.130060	H -0.181550 -2.100650 1.714970
	H 2.329889 0.809123 1.706208	H 2.310123 -1.946766 -0.191632	H 1.754475 0.079392 2.384222
	H -0.049513 2.603466 0.797058	H -0.447888 -2.625279 0.345491	H -1.069928 1.421404 1.841814
	H 2.033748 0.812058 -1.989668	H 1.787548 -0.042964 2.361072	H 1.366526 2.320565 -1.020749
	H -0.249516 -2.374800 -0.797781	H 0.330785 2.468130 -0.696648	H 0.480569 -1.631116 -1.915939
	H -0.049577 -2.603687 0.796252	H 1.177795 1.782360 -1.915369	H 1.147987 -2.513292 -0.713720
	H 1.249137 -2.590941 -0.178525	H 1.935037 2.178929 -0.542244	H 2.076152 -1.480197 -1.554916
	H 2.033642 -0.811505 -1.989948	H 1.717212 1.483355 1.809523	H 2.188696 1.110542 -1.726680
	H 0.732777 0.000452 -2.543032	H 0.383272 0.810754 2.430550	H 0.606947 1.360259 -2.099290
	H 1.248957 2.591016 -0.178042	H 0.011136 -2.003773 1.774917	H 0.084689 2.417468 1.285400

	H -0.249836 2.374976 -0.796992	H -1.406731 -1.549232 1.123113	H -1.185203 2.006213 0.326540
	H 2.329860 -0.809330 1.706145	H 2.361406 -1.063601 -1.549307	H 2.597371 -0.863087 1.367743
	H 3.051960 -0.000071 0.501868	H 3.027164 -0.494953 -0.188388	H 2.700492 0.749513 1.250349
	H -0.693397 -0.810449 2.392455	H -0.455462 -0.019885 -2.569487	H -1.248674 -1.937488 0.473772
	H -1.793660 -0.000580 1.499453	H -1.619149 -0.596779 -1.600679	H -1.392400 -1.013044 1.792446
	H -2.253326 0.000111 -0.616321	H -2.181464 0.637628 0.314939	H -2.275007 0.012888 -0.686712
	H -3.699790 -0.916172 0.302898	H -3.744890 0.841361 -0.757691	H -3.691287 -1.031678 -0.168135
	H -3.973399 0.000412 -1.242358	H -3.895631 0.607395 1.041547	H -3.969009 0.617420 -0.831257
	H -3.699746 0.915895 0.303543	H -3.494907 -0.812824 -0.023829	H -3.298436 0.424905 0.828656
	S=1/2	S=3/2	S=5/2
$(H_3N)_5MnO^{2+}$	Mn 0.321733 0.000302 -0.007187	Mn 0.338501 -0.000384 0.001076	Mn 0.356229 0.031193 0.033650
	O -1.022940 0.093686 -1.090874	O -1.025702 0.158113 -1.107778	O -0.897373 -0.351199 -1.205422
	N 1.121823 -1.449227 -1.265323	N 1.153609 -1.223852 -1.465830	N 1.411604 -1.907055 -0.585005
	N 1.205083 1.508247 -1.139313	N 1.102132 1.660479 -0.992189	N 1.375521 0.987782 -1.626197
	N -0.508206 1.451058 1.216012	N -0.544064 1.261644 1.380724	N -0.707797 1.977428 0.529202
	N 2.096800 -0.121044 1.184179	N 2.085604 -0.197563 1.226433	N 1.842624 0.496219 1.444388
	N -0.585127 -1.463686 1.133234	N -0.512410 -1.639737 0.922876	N -0.774939 -1.125522 1.476412
	C -3.398139 -0.006438 -0.241663	C -3.380845 0.011707 -0.189474	C -3.362276 -0.223930 -0.352519
	H -0.444142 -1.417344 2.142462	H -0.238372 -1.849481 1.882381	H -0.295250 -1.521953 2.284064
	H 2.021151 0.336590 2.092879	H 1.940167 0.050158 2.205071	H 1.458339 0.787580 2.343832
	H -1.017713 1.119392 2.035002	H -1.051848 0.797683 2.133971	H -1.300470 1.974806 1.358822
	H 1.416674 2.384539 -0.662782	H 1.163573 2.531161 -0.464582	H 1.252107 1.998960 -1.675133
	H 0.346660 -1.651495 -1.900015	H 0.339290 -1.417632 -2.053870	H 0.802704 -2.244261 -1.332552
	H 1.403684 -2.339306 -0.855591	H 1.561964 -2.119637 -1.199356	H 1.457768 -2.663763 0.096632
	H 1.901683 -1.163852 -1.856616	H 1.838403 -0.784258 -2.080302	H 2.346630 -1.851685 -0.987179
	H 2.047212 1.271734 -1.663152	H 2.005218 1.562340 -1.455859	H 2.371580 0.816621 -1.760691
	H 0.489463 1.726132 -1.835751	H 0.405480 1.811806 -1.725614	H 0.895711 0.615220 -2.448561
	H 0.111358 2.186913 1.555433	H 0.072005 1.934127 1.837903	H -0.157044 2.832838 0.596727
	H -1.203104 1.910392 0.624006	H -1.242133 1.802519 0.866159	H -1.341368 2.104614 -0.261049
	H 2.371029 -1.082058 1.388728	H 2.446434 -1.151826 1.242116	H 2.461307 -0.291336 1.640871
	H 2.909506 0.301416 0.735648	H 2.869905 0.376561 0.916184	H 2.449571 1.259448 1.143070
	H -0.364898 -2.422978 0.866209	H -0.396136 -2.505650 0.395974	H -1.161148 -1.907387 0.945514
	H -1.590574 -1.364025 0.976548	H -1.521645 -1.475331 0.929430	H -1.585896 -0.624488 1.839839
	H -2.187804 0.039920 -0.678979	H -2.191295 0.085501 -0.653332	H -2.251551 -0.284392 -0.738238
	H -3.704927 -1.034364 -0.402310	H -3.738610 -0.956053 -0.524685	H -3.637576 -1.225635 -0.035364
	H -3.885693 0.698141 -0.906133	H -3.880258 0.841709 -0.676852	H -3.929222 0.094870 -1.221547
	H -3.403127 0.304416 0.799535	H -3.348080 0.113952 0.893345	H -3.418354 0.501331 0.456587
	S=0	S=1	S=2
$(H_3N)_5FeO^{2+}$	Fe 0.351003 -0.001725 0.009023	Fe 0.335253 -0.003224 0.002080	Fe 0.332032 0.000071 0.004069
	O -1.039359 0.109092 -1.078250	O -1.013817 0.302118 -1.059282	O -1.077006 0.000066 -0.995809
	N 1.061182 -1.425779 -1.289947	N 1.148085 -1.076321 -1.526939	N 1.321861 -0.000082 -1.819419
	N 1.143180 1.534159 -1.108193	N 1.168057 1.668125 -0.815929	N 0.410907 2.282929 0.011374
	N -0.510058 1.425494 1.214137	N -0.509023 1.155668 1.448970	N -0.848811 0.000106 1.704311
	N 2.117276 -0.132344 1.125902	N 2.029228 -0.382214 1.180606	N 2.105860 -0.000229 1.101870
	N -0.559931 -1.480301 1.101713	N -0.591065 -1.624164 0.774740	N 0.410359 -2.283099 0.011373
	C -3.397447 -0.009533 -0.201947	C -3.385292 0.021578 -0.223297	C -3.484991 0.000165 -0.222304
	H -0.468630 -1.428016 2.116516	H -0.349401 -1.887216 1.729972	H 0.132722 -2.764804 0.865864
	H 2.102212 0.407429 1.991529	H 1.855024 -0.345695 2.185091	H 2.183729 -0.815243 1.710232
	H -1.065596 1.075102 1.994414	H -1.069233 0.651070 2.135667	H -1.470898 -0.808629 1.683680
	H 1.381571 2.388130 -0.605018	H 1.206013 2.494123 -0.218651	H 0.133173 2.764642 0.865829
	H 0.222508 -1.682501 -1.815770	H 0.450655 -0.990029 -2.269810	H 0.572564 -0.000145 -2.514793
	H 1.456468 -2.280692 -0.900138	H 1.285927 -2.075109 -1.374123	H 1.903803 -0.815480 -2.009133
	H 1.736461 -1.101464 -1.981472	H 2.029878 -0.734769 -1.907596	H 1.903791 0.815295 -2.009262
	H 1.952516 1.322981 -1.691172	H 2.096809 1.586137 -1.228458	H 1.274306 2.740253 -0.279329
	H 0.377680 1.767199 -1.745521	H 0.521605 1.897135 -1.574623	H -0.290021 2.531836 -0.687130
	H 0.088465 2.154092 1.602731	H 0.120264 1.750946 1.988151	H -0.389399 0.000211 2.614230
	H -1.173497 1.883645 0.585724	H -1.158806 1.774547 0.960756	H -1.471036 0.808731 1.683556
	H 2.351690 -1.084909 1.405083	H 2.420751 -1.308500 1.008873	H 2.950648 -0.000384 0.530848
	H 2.929406 0.206690 0.610626	H 2.798027 0.267629 1.015744	H 2.184072 0.814710 1.710292
	H -0.287800 -2.429706 0.848453	H -0.482943 -2.468900 0.213471	H 1.273518 -2.740681 -0.279629
	H -1.557508 -1.412331 0.889231	H -1.594147 -1.426260 0.761487	H -0.290850 -2.531705 -0.686955
	H -2.218934 0.041424 -0.644681	H -2.165329 0.153470 -0.637094	H -2.244939 0.000187 -0.548366
	H -3.722982 -1.027283 -0.389928	H -3.685137 -0.972625 -0.537317	H -3.953781 0.000110 -1.199719
	H -3.905203 0.719560 -0.823600	H -3.882900 0.811159 -0.774992	H -3.654761 0.913350 0.338832
	H -3.386901 0.261377 0.850801	H -3.408331 0.172659 0.853280	H -3.654718 -0.912991 0.338892
	S=3		
$(H_3N)_5FeO^{2+}$	Fe 0.296820 0.002439 -0.014538		
	O -1.015226 -0.005339 -1.316506		
	N 1.314524 -1.676296 -1.044376		
	N 1.430670 1.346114 -1.359575		
	N -0.568633 1.746512 1.027052		
	N 1.947655 0.049717 1.516960		
	N -0.781961 -1.459666 1.232739		
	C -3.378909 -0.008642 -0.294622		
	H -0.255094 -2.026171 1.896586		
	H 1.603886 0.143813 2.472544		
	H -1.113396 1.538453 1.863576		
	H 1.299325 2.338987 -1.168326		
	H 0.786265 -1.805552 -1.909069		
	H 1.289031 -2.576447 -0.566389		

	H	2.286479	-1.539446	-1.319410								
	H	2.434978	1.215569	-1.474795								
	H	1.007704	1.193750	-2.277241								
	H	0.081260	2.482511	1.301859								
	H	-1.223285	2.167508	0.366466								
	H	2.528359	-0.788458	1.517627								
	H	2.594039	0.827328	1.384423								
	H	-1.226935	-2.107358	0.581112								
	H	-1.551892	-1.048676	1.760764								
	H	-2.278611	-0.007965	-0.788602								
	H	-3.676395	-1.051220	-0.236498								
	H	-3.969185	0.551459	-1.012830								
	H	-3.334369	0.478385	0.676375								
	S=1/2				S=3/2				S=5/2			
$(H_3N)_5CoO^{2+}$	Co	0.363379	-0.001304	0.017262	Co	-0.363815	0.001362	0.053159	Co	0.295486	0.001704	-0.002914
	O	-1.033020	0.045215	-1.131031	O	0.973982	-0.013840	-1.134352	O	-1.079452	-0.036270	-1.219014
	N	1.025404	-1.467890	-1.133368	N	-1.154418	1.579385	-1.141058	N	1.220109	-1.678615	-1.057891
	N	1.163770	1.316005	-1.223711	N	-1.291178	-1.369104	-1.292208	N	1.316954	1.307517	-1.430932
	N	-0.450065	1.505304	1.000375	N	0.553069	-1.648637	1.009816	N	-0.482197	1.740894	1.044948
	N	1.997143	-0.047678	1.244181	N	-1.931988	0.016058	1.344352	N	2.042208	0.060963	1.359094
	N	-0.609026	-1.353111	1.078798	N	0.725008	1.443595	1.151179	N	-0.707491	-1.401567	1.316176
	C	-3.337802	0.014178	-0.097210	C	3.356890	-0.012895	-0.292615	C	-3.438594	-0.005517	-0.264117
	H	-1.178787	-1.861413	0.399122	H	1.192932	2.008534	0.441294	H	-0.158983	-1.836030	2.057067
	H	1.754450	0.029271	2.232109	H	-1.638969	-0.075487	2.316986	H	1.780918	0.178021	2.337330
	H	-0.979384	1.265084	1.838109	H	1.173696	-1.419230	1.785615	H	-0.926544	1.530696	1.938083
	H	1.355214	2.245199	-0.849601	H	-1.386785	-2.330684	-0.966976	H	1.070120	2.291784	-1.333476
	H	0.292090	-1.519247	-1.847685	H	-0.466223	1.635326	-1.894519	H	0.661292	-1.869007	-1.889851
	H	1.090170	-2.390756	-0.704002	H	-1.193538	2.503470	-0.711810	H	1.268837	-2.557617	-0.544145
	H	1.913334	-1.323353	-1.613225	H	-2.065410	1.439936	-1.576504	H	2.168361	-1.500529	-1.385892
	H	2.011210	1.036673	-1.717079	H	-2.197496	-1.120626	-1.686931	H	2.334250	1.278490	-1.477620
	H	0.422125	1.422438	-1.923354	H	-0.621544	-1.391956	-2.063950	H	0.972547	1.038734	-2.353069
	H	0.180557	2.257292	1.278510	H	-0.057612	-2.388936	1.354229	H	0.215885	2.454544	1.251727
	H	-1.126184	1.894417	0.338511	H	1.146991	-2.053830	0.284681	H	-1.197157	2.194931	0.476663
	H	2.542586	-0.905855	1.163987	H	-2.478377	0.875584	1.293788	H	2.609841	-0.785037	1.328558
	H	2.659552	0.707670	1.068039	H	-2.591229	-0.743617	1.176423	H	2.685963	0.825148	1.157909
	H	-1.277367	-0.972530	1.748816	H	1.479209	1.042235	1.708367	H	-1.089115	-2.162703	0.754404
	H	-0.048566	-2.035620	1.589586	H	0.221451	2.080375	1.767847	H	-1.516656	-0.980407	1.772089
	H	-2.178980	0.028260	-0.608760	H	2.162866	-0.011325	-0.677009	H	-2.244567	-0.022466	-0.749322
	H	-3.641517	-1.027557	-0.089080	H	3.653182	1.030543	-0.268936	H	-3.701893	-1.054297	-0.178662
	H	-3.910746	0.612121	-0.797172	H	3.858114	-0.578051	-1.070487	H	-3.975241	0.519990	-1.046125
	H	-3.290620	0.457912	0.894686	H	3.395107	-0.500021	0.678534	H	-3.395895	0.528649	0.680057
	S=0				S=1				S=2			
$(H_3N)_5NiO^{2+}$	Ni	0.362623	-0.012391	0.042478	Ni	0.361923	-0.029446	0.033224	Ni	0.341540	0.002852	0.016557
	O	-0.966746	0.222056	-1.120088	O	-0.974408	0.439492	-1.046285	O	-1.180920	-0.169093	-1.178842
	N	1.107455	-1.351541	-1.353451	N	1.155235	-0.863805	-1.630269	N	1.153823	-1.706151	-1.020419
	N	1.301313	1.557663	-1.026753	N	1.251645	1.863034	-0.540636	N	1.183133	1.243301	-1.536877
	N	-0.519439	1.411041	1.241910	N	-0.607237	0.940741	1.513605	N	-0.456880	1.747571	0.975026
	N	1.892688	-0.263145	1.298800	N	1.908003	-0.528559	1.200860	N	2.114557	0.156529	1.219794
	N	-0.732940	-1.596053	0.896100	N	-0.645740	-1.887025	0.426736	N	-0.591953	-1.304771	1.439968
	C	-3.348546	0.097812	-0.267072	C	-3.352598	0.204228	-0.214035	C	-3.541853	-0.010683	-0.196561
	H	-0.209154	-2.354624	1.331314	H	-0.073578	-2.730709	0.432085	H	0.006579	-1.623069	2.200679
	H	1.589199	-0.327127	2.270343	H	1.608186	-0.950782	2.079481	H	1.910688	0.365402	2.196257
	H	-1.134199	1.056515	1.973148	H	-1.363816	0.397811	1.928186	H	-0.838052	1.586634	1.906045
	H	1.437089	2.437965	-0.531432	H	1.320710	2.584334	0.176159	H	1.017711	2.243091	-1.432898
	H	0.424214	-1.273607	-2.109211	H	0.456433	-0.648884	-2.345029	H	0.566403	-1.945623	-1.818901
	H	1.130457	-2.332098	-1.076098	H	1.264665	-1.877014	-1.614290	H	1.232076	-2.556987	-0.465596
	H	2.023345	-1.146513	-1.750334	H	2.044762	-0.479111	-1.945280	H	2.084010	-1.538258	-1.400300
	H	2.187973	1.355931	-1.486621	H	2.155058	1.858410	-1.012039	H	2.187669	1.141532	-1.671865
	H	0.617069	1.741706	-1.762553	H	0.564470	2.194642	-1.219346	H	0.751934	0.987000	-2.424834
	H	0.113926	2.068607	1.695352	H	-0.040073	1.296937	2.282129	H	0.239045	2.483136	1.089972
	H	-1.109247	1.945530	0.602895	H	-1.053514	1.738121	1.059164	H	-1.212699	2.162815	0.431349
	H	2.423518	-1.112804	1.109330	H	2.540048	-1.200520	0.766731	H	2.669279	-0.698218	1.232790
	H	2.562991	0.504134	1.257394	H	2.480707	0.278351	1.447375	H	2.751749	0.890334	0.913279
	H	-1.251193	-1.995692	0.113511	H	-1.296009	-1.972104	-0.354945	H	-0.934987	-2.148886	0.983129
	H	-1.439373	-1.311837	1.573553	H	-1.211790	-1.934960	1.272761	H	-1.408853	-0.887260	1.883452
	H	-2.160519	0.149827	-0.651726	H	-2.164348	0.300080	-0.595022	H	-2.356148	-0.081400	-0.668121
	H	-3.660371	-0.926457	-0.442565	H	-3.875421	-0.072568	-1.122883	H	-3.823993	-1.052856	-0.091264
	H	-3.843344	0.809569	-0.918690	H	-3.616272	1.194254	0.142896	H	-4.056839	0.513525	-0.994217
	H	-3.374119	0.388830	0.779889	H	-3.396550	-0.563808	0.554002	H	-3.468950	0.540710	0.735095

Table S5. Optimized geometries (Cartesian coordinates in Å) for the product of the reaction between methane and $(NH_3)_5MO^{2+}$ species.

	S=0				S=1							
$(H_3N)_5TiO^{2+}$	Ti	-0.549267	0.000448	-0.164714	Ti	-0.334654	0.000004	-0.001478				
	O	-1.454775	0.004625	-1.679163	O	1.416010	-0.002407	-0.010578				
	N	-1.837126	1.586453	0.796696	N	-0.478068	1.609463	1.578918				
	N	0.703477	-0.008392	1.763538	N	-2.653651	0.001872	0.047388				
	N	0.938070	1.549034	-0.834908	N	-0.481893	-1.607738	1.580409				
	N	0.933582	-1.550500	-0.839967	N	-0.498725	-1.587495	-1.602846				
	N	-1.846603	-1.581902	0.790665	N	-0.495313	1.586777	-1.603830				
	C	3.973048	0.000183	0.236237	C	4.461249	-0.000847	0.016266				
	H	4.051535	-0.927593	0.781475	H	4.566884	-0.896636	-0.573208				
	H	4.167874	-0.002654	-0.825197	H	4.565431	0.958466	-0.463273				
	H	4.055538	0.930125	0.777056	H	4.550388	-0.064276	1.088254				
	H	-1.942262	0.005651	-2.508642	H	2.397663	-0.002612	-0.001395				
	H	-2.564997	1.763454	0.102549	H	0.474393	1.971223	1.644420				
	H	-2.330908	1.347655	1.656032	H	-1.070509	2.418494	1.395971				
	H	0.567218	-0.825962	2.357555	H	-3.074740	-0.809362	-0.405057				
	H	0.561695	0.797819	2.371595	H	-3.073994	0.811547	-0.408536				
	H	0.797132	1.655224	-1.840395	H	0.471398	-1.966286	1.651283				
	H	1.917093	1.279734	-0.710757	H	-1.070510	-2.418917	1.394658				
	H	1.913037	-1.278604	-0.726369	H	0.463823	-1.706613	-1.921750				
	H	0.783723	-1.663199	-1.843453	H	-1.041395	-1.376109	-2.439488				
	H	-1.435891	-2.506072	0.919306	H	-1.039514	1.376488	-2.439758				
	H	-2.300689	-1.362267	1.676575	H	-0.795445	2.516467	-1.312338				
	H	-2.604308	-1.716231	0.119292	H	0.467226	1.702856	-1.923901				
	H	0.859707	-2.488323	-0.447579	H	-0.801919	-2.516113	-1.311117				
	H	0.858615	2.489186	-0.449223	H	-0.726438	-1.308821	2.523818				
	H	1.698865	-0.003125	1.523039	H	-3.026171	0.004089	0.996828				
	H	-1.408985	2.494391	0.974568	H	-0.716219	1.310214	2.523858				
	S=1/2				S=3/2							
$(H_3N)_5VO^{2+}$	V	-0.362664	-0.002397	0.028693	V	-0.371621	0.000054	0.024373				
	O	0.987125	-0.024407	1.167593	O	0.943634	0.001032	1.228343				
	N	-1.650146	-0.062756	1.801563	N	-1.679742	0.000011	1.779419				
	N	-0.516894	2.171184	0.033845	N	-0.516369	2.174046	-0.027870				
	N	1.009832	0.063540	-1.668622	N	1.061465	0.000224	-1.627772				
	N	-2.125215	0.036051	-1.351705	N	-2.092291	-0.001378	-1.414496				
	N	-0.497436	-2.173119	-0.101253	N	-0.514301	-2.174070	-0.026623				
	C	4.050255	-0.008718	0.227090	C	4.073891	0.000155	0.215510				
	H	-0.099707	-2.619423	-0.927222	H	-0.041139	-2.649008	-0.794974				
	H	-2.153704	-0.761154	-1.987271	H	-2.094953	-0.809889	-2.036373				
	H	1.022759	-0.749880	-2.283213	H	1.041255	-0.807745	-2.249541				
	H	-0.104876	2.670842	-0.753755	H	-0.044608	2.649048	-0.797042				
	H	-0.956874	-0.093931	2.552273	H	-0.992486	0.000776	2.536752				
	H	-2.252600	-0.873153	1.941107	H	-2.278150	-0.809657	1.939091				
	H	-2.239697	0.745179	1.998763	H	-2.279112	0.809125	1.938294				
	H	-1.445426	2.573516	0.159432	H	-1.450434	2.580900	0.015629				
	H	0.026863	2.455456	0.850642	H	-0.032185	2.480771	0.817951				
	H	0.938617	0.865865	-2.293938	H	1.041938	0.808996	-2.248517				
	H	1.949813	0.111353	-1.259236	H	1.995744	-0.000410	-1.202156				
	H	-3.023887	0.032000	-0.868779	H	-3.007243	-0.002814	-0.963268				
	H	-2.144698	0.857818	-1.955577	H	-2.097147	0.807729	-2.035586				
	H	-1.421903	-2.587127	0.016440	H	-1.448060	-2.581730	0.015920				
	H	0.062356	-2.507457	0.685221	H	-0.030878	-2.479961	0.819933				
	H	1.955031	-0.033908	1.051261	H	1.906503	0.001187	1.095787				
	H	4.094525	-0.009927	1.305505	H	4.080533	0.000098	1.294718				
	H	4.205163	0.922374	-0.294154	H	4.236997	0.931330	-0.302796				
	H	4.190006	-0.940059	-0.297535	H	4.236957	-0.931018	-0.302812				
	S=0				S=1				S=2			
$(H_3N)_5CrO^{2+}$	Cr	0.391879	-0.000002	-0.001183	Cr	-0.402730	-0.011991	0.020522	Cr	-0.401900	0.006046	0.006772
	O	-0.919492	0.006685	-1.214912	O	0.432948	-0.140270	1.602555	O	0.471149	-0.146352	-1.594082
	N	-1.041131	0.000579	1.552151	N	-1.737195	-1.570274	0.584888	N	-1.651465	1.432807	-0.941156
	N	2.063281	-0.006533	1.387886	N	-1.687034	1.396678	0.942542	N	-1.648335	-1.525228	-0.763971
	N	0.423654	2.126187	-0.027228	N	0.911905	1.555414	-0.522073	N	0.873132	-1.472210	0.807752
	N	1.740053	-0.003165	-1.636176	N	-1.478857	0.231290	-1.847161	N	-1.541051	0.141065	1.845123
	N	0.407260	-2.126325	-0.031706	N	0.949930	-1.426142	-0.774325	N	0.918678	1.529770	0.619066
	C	-4.010869	0.004132	-0.196121	C	3.911062	0.001701	0.132013	C	3.860930	-0.002719	-0.154999
	H	-4.061994	1.053577	-0.437855	H	0.884517	-1.657916	-1.765665	H	0.841871	1.918412	1.558881
	H	-4.027034	-0.720504	-0.995089	H	-2.127497	1.018606	-1.849264	H	-0.973003	0.059166	2.688764
	H	-4.289479	-0.317576	0.795258	H	0.694507	2.186978	-1.292337	H	1.804969	-1.095795	1.000247
	H	-1.880702	0.003006	-1.071053	H	-1.632268	2.363389	0.622863	H	-1.596422	-2.439535	-0.315443
	H	-1.968739	-0.020780	1.106030	H	-1.511277	-1.720825	1.570269	H	-1.291078	1.405152	-1.898498
	H	-1.017084	-0.798876	2.185062	H	-1.616710	-2.473085	0.126267	H	-1.582894	2.401982	-0.631701
	H	2.979206	0.000344	0.939014	H	-2.737559	-1.376554	0.545770	H	-2.650335	1.235395	-0.994176
	H	2.070313	-0.821229	2.002129	H	-2.682117	1.184540	1.005749	H	-2.642756	-1.336897	-0.886059
	H	-0.055429	2.347594	-0.902240	H	-1.328574	1.390092	1.901269	H	-1.247641	-1.635284	-1.699885
	H	1.326638	2.598771	-0.058603	H	1.014218	2.131771	0.314568	H	0.597309	-2.007644	1.630627

	H 1.088870 0.000147 -2.425717	H 1.854053 1.192156 -0.695160	H 1.019854 -2.138221 0.047323
	H 2.340172 -0.817431 -1.763536	H -0.864647 0.386550 -2.647189	H -2.044015 1.023293 1.943963
	H 1.306209 -2.607083 -0.053577	H -2.042262 -0.581877 -2.097237	H -2.253529 -0.584286 1.930613
	H -0.138882 -2.596514 0.689591	H 0.902720 -2.313811 -0.273728	H 0.871362 2.320216 -0.024677
	H -0.064354 -2.343234 -0.911849	H 1.907181 -1.084268 -0.620159	H 1.878610 1.174581 0.523582
	H 2.346377 0.806712 -1.762089	H 1.246538 -0.589040 1.859539	H 1.399876 0.048948 -1.764024
	H -0.109832 2.601000 0.700429	H 4.138118 -1.052551 0.162941	H 4.006434 0.975524 -0.586214
	H 2.064275 0.798396 2.014940	H 3.667921 0.510249 1.051932	H 3.689626 -0.839445 -0.814405
	H -1.044303 0.820253 2.159069	H 4.257478 0.576565 -0.712574	H 4.225866 -0.186966 0.843510
	S=1/2	S=3/2	S=5/2
$(H_3N)_5MnO^{2+}$	Mn 0.383777 -0.005396 -0.017130	Mn 0.391396 -0.003795 -0.005957	Mn 0.385721 0.000047 -0.036420
	O -0.571417 0.217361 -1.511239	O -0.555816 0.070031 -1.519600	O -0.847311 -0.000598 1.227819
	N 1.583847 -1.401351 -0.987068	N -0.876467 -1.456703 0.727639	N -1.150072 0.001072 -1.702378
	N 1.589500 1.509779 -0.772447	N 1.602247 -0.070941 1.726116	N 1.922527 0.000792 -1.466233
	N -0.784221 1.428747 0.928973	N -0.810892 1.468844 0.805161	N 0.411050 -2.179119 0.050124
	N 1.593805 -0.209207 1.733935	N 1.587440 1.466263 -0.846880	N 1.890936 -0.001239 1.687632
	N -0.898067 -1.482976 0.645971	N 1.569011 -1.454783 -0.902009	N 0.411470 2.179091 0.052773
	C -3.810373 -0.002697 -0.167987	C -3.822277 0.000076 -0.155497	C -3.987854 -0.000193 0.431445
	H -0.856616 -1.743305 1.631347	H -4.171167 0.033982 0.864962	H -4.190203 -0.930951 -0.073914
	H 1.093462 -0.017892 2.602269	H -3.708897 0.924924 -0.699691	H -3.915638 -0.000653 1.508203
	H -1.632902 1.055951 1.357075	H -3.933119 -0.918718 -0.710329	H -4.189912 0.931001 -0.073220
	H 1.577271 2.403686 -0.282212	H -1.511916 -0.063480 -1.560423	H -1.800365 -0.000591 1.020662
	H 1.239166 -1.359705 -1.948532	H -1.843488 -1.124902 0.627060	H -1.145135 -0.808502 -2.322127
	H 1.503180 -2.374742 -0.694498	H -0.829927 -2.306281 0.165020	H -2.070810 0.000641 -1.252368
	H 2.584492 -1.209784 -1.028282	H 2.325603 0.648426 1.734085	H 2.847622 0.000335 -1.035241
	H 2.572224 1.308862 -0.954557	H 2.097292 -0.956823 1.830943	H 1.897731 0.815324 -2.080122
	H 1.149645 1.672198 -1.682213	H -1.077410 2.052144 0.010353	H 0.056598 -2.368680 0.988702
	H -0.356241 2.042937 1.621628	H -0.416781 2.092419 1.509848	H 1.298455 -2.672871 -0.038360
	H -1.110838 2.020481 0.163014	H 1.188932 1.531954 -1.787226	H 1.245136 -0.001518 2.479039
	H 1.980135 -1.146970 1.844992	H 2.582952 1.278954 -0.962059	H 2.495730 0.808673 1.818151
	H 2.398159 0.418404 1.741764	H 2.556225 -1.242717 -1.043091	H 1.299007 2.672834 -0.034460
	H -0.823670 -2.353871 0.120393	H 1.543221 -2.395967 -0.510530	H -0.234980 2.647773 -0.581266
	H -1.855403 -1.144219 0.475356	H 1.147655 -1.516372 -1.832167	H 0.056446 2.367448 0.991380
	H -1.448843 -0.133608 -1.708901	H 1.526412 2.402424 -0.447184	H 2.495255 -0.811661 1.817210
	H -4.011642 -0.912790 -0.711279	H -1.693290 1.121412 1.185332	H -0.235907 -2.646814 -0.584123
	H -3.615087 0.901380 -0.723144	H 1.082966 0.056352 2.595457	H 1.897511 -0.812768 -2.081402
	H -4.141379 0.070243 0.856319	H -0.769332 -1.752323 1.698151	H -1.145333 0.811589 -2.320898
	S=0	S=1	S=2
$(H_3N)_5FeO^{2+}$	Fe 0.393449 -0.000648 0.011297	Fe 0.392882 -0.000195 0.011194	Fe 0.382535 0.000141 0.011341
	O -0.525033 -0.181340 -1.517022	O -0.547045 0.209094 -1.499283	O -0.862230 -0.005080 -1.244017
	N 1.530965 -1.561041 -0.650191	N 1.570215 -1.239665 -1.099628	N 0.456203 -2.289103 -0.019609
	N 1.597652 1.244448 -1.065556	N 1.533138 1.560315 -0.646475	N 1.927476 0.003645 1.383871
	N -0.823062 1.538395 0.541428	N -0.830653 1.313188 0.958370	N -1.022226 0.005650 1.501550
	N 1.562104 0.265549 1.710050	N 1.586507 -0.301487 1.686988	N 0.447559 2.289955 -0.028725
	N -0.854655 -1.312637 0.923811	N -0.831154 -1.530807 0.546290	N 1.648740 -0.001194 -1.616348
	C -3.833329 -0.009478 -0.156440	C -3.826779 0.009488 -0.161132	C -3.981261 -0.005575 -0.187526
	H -0.678032 -1.570206 1.894998	H -0.682111 -1.995667 1.441637	H -4.128262 -1.022352 0.139981
	H 1.028843 0.366398 2.573860	H 1.069602 -0.371572 2.563685	H -4.210981 0.804175 0.487112
	H -1.794635 1.215358 0.588940	H -1.790162 0.953331 0.961288	H -3.951939 0.199412 -1.246674
	H 1.571805 2.236394 -0.830103	H 1.509712 2.423279 -0.103179	H -1.797585 -0.003005 -0.973307
	H 1.092491 -1.750336 -1.555376	H 1.148499 -1.140807 -2.027149	H -0.002043 -2.482747 -0.910863
	H 1.498396 -2.429631 -0.116445	H 1.548457 -2.233495 -0.871981	H 1.353861 -2.771327 -0.046409
	H 2.519332 -1.385052 -0.826674	H 2.557053 -1.000431 -1.188357	H 1.912498 0.829203 1.983005
	H 2.585912 1.004830 -1.135624	H 2.518946 1.381817 -0.834433	H 2.854345 -0.014417 0.959071
	H 1.195236 1.154116 -2.002459	H 1.086820 1.761708 -1.545162	H -1.967582 0.026538 1.095286
	H -0.635426 2.045496 1.406050	H -0.616400 1.584881 1.918031	H -0.958404 0.810210 2.125429
	H -0.820149 2.227787 -0.211061	H -0.881284 2.173806 0.412092	H -0.011133 2.478436 -0.920849
	H 2.205005 -0.509225 1.876307	H 2.136135 -1.158589 1.625060	H 1.342554 2.777077 -0.056732
	H 2.151806 1.096002 1.654399	H 2.267745 0.444228 1.831842	H 2.248889 0.815616 -1.727259
	H -0.883587 -2.181439 0.389087	H -0.799028 -2.257066 -0.169761	H 2.253351 -0.815157 -1.723823
	H -1.814895 -0.953860 0.883320	H -1.803415 -1.204135 0.536473	H 0.991242 -0.004666 -2.400396
	H -1.490282 -0.234450 -1.471365	H -1.511596 0.262497 -1.436501	H -0.105957 2.791266 0.665165
	H -3.868142 -1.020425 -0.532367	H -3.817877 -0.787698 -0.888497	H -0.986811 -0.818121 2.102247
	H -3.795798 0.803815 -0.864940	H -3.842248 1.029613 -0.512950	H 1.893077 -0.801078 2.009931
	H -4.188338 0.185872 0.843748	H -4.173116 -0.201128 0.839065	H -0.093888 -2.791316 0.676329
	S=3		
$(H_3N)_5FeO^{2+}$	Fe -0.350446 -0.001383 0.000306		
	O 1.408007 -0.004278 -0.027545		
	N -0.542488 1.778164 -1.304305		
	N -0.460460 -1.254471 -1.819106		
	N -0.474484 -1.800250 1.287462		
	N -2.605629 -0.046175 0.094539		
	N -0.393195 1.344565 1.754807		
	C 4.517611 -0.015620 0.009538		
	H -1.242789 1.833733 2.032154		
	H -2.953141 -0.042541 1.053015		
	H -0.826067 -1.651965 2.232658		
	H -0.198651 -2.219857 -1.620919		
	H 0.414108 2.039903 -1.544319		

	H -0.964553 2.603866 -0.881097		
	H -1.030671 1.640691 -2.188445		
	H -1.307991 -1.299063 -2.382800		
	H 0.279610 -0.905714 -2.428758		
	H -1.003259 -2.591354 0.922285		
	H 0.488954 -2.119750 1.391171		
	H -3.062707 0.740378 -0.365499		
	H -3.003243 -0.881938 -0.332955		
	H 0.310464 2.059921 1.570402		
	H -0.055704 0.861526 2.587087		
	H 2.388866 -0.007357 -0.011062		
	H 4.623280 0.940326 -0.476245		
	H 4.605654 -0.915613 -0.576398		
	H 4.613527 -0.074132 1.081126		
	S=1/2	S=3/2	S=5/2
$(H_3N)_5CoO^{2+}$	Co -0.405721 0.004506 0.027107	Co 0.371136 -0.001630 -0.006170	Co -0.332424 -0.001383 -0.001705
	O 0.447403 -0.094110 -1.585603	O -0.877266 0.016866 -1.238685	O 1.393103 -0.018382 -0.008371
	N -1.545333 1.349820 -0.864968	N 0.419615 -2.265745 -0.015070	N -0.526830 0.258191 -2.149586
	N -1.544561 -1.420840 -0.733851	N 1.588243 -0.013446 -1.572994	N -0.459062 -2.151305 -0.308615
	N 0.814612 -1.394800 0.686548	N 0.478121 2.262510 -0.010425	N -0.483177 -0.267620 2.152450
	N -1.446142 0.095650 1.762154	N 1.914902 -0.017267 1.342562	N -2.550632 0.022835 0.082669
	N 0.835544 1.438707 0.559712	N -0.955437 0.006971 1.455318	N -0.405943 2.163236 0.215105
	C 3.839454 -0.003419 -0.071728	C -3.963597 0.017829 -0.122541	C 4.471814 -0.006946 0.020756
	H 0.842510 2.149248 -0.173211	H -1.907627 -0.034891 1.065772	H -1.248351 2.671268 -0.049423
	H -0.850947 0.037084 2.588811	H 1.881556 0.782983 1.974116	H -2.901522 0.752278 0.702235
	H 1.742228 -1.036642 0.930088	H -0.021422 2.775499 0.714802	H -0.885040 0.510378 2.672917
	H -1.503901 -2.334005 -0.281743	H 2.196437 0.798683 -1.670766	H -0.278601 -2.679613 0.543685
	H -1.115930 1.358697 -1.796377	H -0.072859 -2.455117 -0.888335	H 0.427959 0.270147 -2.507626
	H -1.516915 2.302080 -0.501363	H -0.107264 -2.761150 0.703006	H -0.957314 1.129439 -2.455377
	H -2.533422 1.127561 -0.981372	H 1.312035 -2.754391 -0.076635	H -1.010026 -0.487189 -2.648368
	H -2.535331 -1.220190 -0.865983	H 2.177813 -0.839149 -1.671321	H -1.298600 -2.552685 -0.723085
	H -1.118876 -1.518464 -1.661986	H 0.933998 -0.005402 -2.360306	H 0.313506 -2.379561 -0.933605
	H 0.505565 -1.975672 1.466165	H 1.383659 2.724131 -0.086793	H -0.978863 -1.099070 2.469699
	H 0.975113 -2.007732 -0.115272	H -0.021922 2.464626 -0.876546	H 0.478635 -0.363276 2.477561
	H -1.974879 0.961675 1.867363	H 1.903670 -0.849046 1.932847	H -3.015680 0.174444 -0.811617
	H -2.131124 -0.653040 1.865823	H 2.839201 0.006270 0.912892	H -2.933967 -0.848346 0.447581
	H 1.800926 1.087293 0.583850	H -0.917458 0.847519 2.031225	H 0.347714 2.521419 -0.370674
	H 0.670781 1.919920 1.444082	H -0.866179 -0.786154 2.089904	H -0.173828 2.462909 1.160790
	H 1.407568 0.016486 -1.561847	H -1.811704 0.020131 -0.963898	H 2.378896 -0.010870 0.004913
	H 4.007529 1.024070 -0.355040	H -3.974265 -0.468676 -1.085732	H 4.582436 0.191432 -1.032728
	H 3.727119 -0.743059 -0.849671	H -4.054910 1.091319 -0.078809	H 4.561105 -1.020437 0.376180
	H 4.141655 -0.323324 0.913819	H -4.211839 -0.566229 0.750155	H 4.570787 0.806028 0.721243
	S=0	S=1	S=2
$(H_3N)_5NiO^{2+}$	Ni -0.394019 0.032626 0.041124	Ni -0.393882 0.033751 0.041160	Ni 0.371309 -0.001530 0.000546
	O 0.574521 -0.495186 -1.376117	O 0.570362 -0.496807 -1.378067	O -0.973409 -0.082928 -1.332144
	N -1.432933 1.096910 -1.303130	N -1.435199 1.099180 -1.298867	N 1.414533 -1.518004 -1.102170
	N -1.653674 -1.685479 -0.437734	N -1.658900 -1.683101 -0.436623	N 1.338766 1.458848 -1.240227
	N 0.767480 -1.137782 1.151768	N 0.765386 -1.141707 1.147752	N -0.645777 1.565126 1.066625
	N -1.545800 0.569434 1.564354	N -1.540709 0.572381 1.567650	N 1.952824 0.055065 1.448926
	N 0.931429 1.726035 0.298283	N 0.937711 1.722454 0.294803	N -0.677294 -1.482108 1.150892
	C 3.775724 -0.244950 -0.181291	C 3.777205 -0.246691 -0.179938	C -3.866185 -0.006944 -0.228306
	H 0.567819 2.577077 0.726331	H 0.591643 2.565749 0.751738	H -0.345030 -1.631695 2.102341
	H -1.008878 0.944233 2.346423	H -1.000158 0.935084 2.352915	H 1.625434 0.244931 2.395166
	H 1.741690 -0.830452 1.058204	H 1.740139 -0.835375 1.057021	H -1.124280 1.245087 1.907387
	H -1.686222 -2.466330 0.216474	H -1.695394 -2.462737 0.218834	H 1.118133 2.429191 -1.022358
	H -0.961419 0.825724 -2.169822	H -0.972671 0.822111 -2.168522	H 0.933731 -1.652493 -1.991476
	H -1.370240 2.111091 -1.222671	H -1.364738 2.113150 -1.222164	H 1.463445 -2.437322 -0.666112
	H -2.421563 0.868905 -1.398623	H -2.426175 0.877776 -1.385075	H 2.375342 -1.271322 -1.333799
	H -2.619403 -1.531689 -0.723997	H -2.623495 -1.526911 -0.725422	H 2.355204 1.407237 -1.281573
	H -1.145652 -2.010110 -1.262415	H -1.150373 -2.010742 -1.259795	H 1.005465 1.308595 -2.192359
	H 0.564510 -1.229469 2.146684	H 0.561230 -1.238732 2.141911	H -0.028014 2.317123 1.369384
	H 0.738093 -2.070799 0.740641	H 0.735192 -2.072198 0.730984	H -1.362398 2.005602 0.490215
	H -2.230602 1.284921 1.322007	H -2.217002 1.297615 1.330417	H 2.461461 -0.826239 1.506363
	H -2.078026 -0.221561 1.926739	H -2.082143 -0.215255 1.923655	H 2.657410 0.766138 1.258004
	H 1.218569 1.971241 -0.649498	H 1.201534 1.987548 -0.654374	H -0.674572 -2.398608 0.705541
	H 1.795908 1.504935 0.793152	H 1.814151 1.484059 0.760173	H -1.661341 -1.222048 1.227920
	H 1.529733 -0.443341 -1.207003	H 1.526036 -0.435994 -1.215031	H -1.934122 -0.080396 -1.075889
	H 3.795474 0.555822 -0.904365	H 3.805457 0.551372 -0.905704	H -4.033226 -1.052543 -0.435896
	H 3.784964 -1.264153 -0.535435	H 3.774215 -1.267139 -0.530658	H -4.040257 0.711241 -1.015174
	H 4.101752 -0.042213 0.827603	H 4.107093 -0.044264 0.827770	H -3.886012 0.332956 0.795708

Table S6. Fully optimized geometries (Cartesian coordinates in Å) for the encounter complex between methanol and $(NH_3)_5MO^{2+}$ species.

	S=1/2	S=3/2	
$(H_3N)_5VO^{2+}$	V -0.644385 -0.006968 0.186133	V -0.735570 0.007665 0.029151	
	O 0.138687 -0.044632 1.524217	O 0.355647 0.135645 1.501964	
	N -2.090410 1.473781 0.864608	N -1.645234 1.766614 0.930096	
	N 0.585528 1.532507 -0.664858	N 0.537505 1.237450 -1.194100	
	N 0.589694 -1.479317 -0.771219	N 0.541856 -1.676215 -0.361817	
	N -1.850826 0.100202 -1.864658	N -2.068265 -0.144086 -1.758498	
	N -2.055675 -1.578137 0.714608	N -2.172580 -1.292199 1.034780	
	C 3.711610 -0.019942 0.724713	C 3.743687 0.118225 0.671170	
	H -2.061166 -2.390029 0.097619	H -2.214294 -2.251005 0.688799	
	H -1.255113 -0.021989 -2.683376	H -2.922319 -0.678055 -1.598680	
	H 0.652932 -2.321021 -0.198579	H 0.580210 -2.293849 0.449732	
	H 0.354611 1.913447 -1.580726	H 1.499220 0.900110 -1.009314	
	H -1.837116 2.436466 0.642174	H -0.967712 2.118088 1.611825	
	H -2.101505 1.427580 1.884750	H -2.499589 1.603343 1.462519	
	H -3.058118 1.368728 0.561904	H -1.854678 2.550430 0.311918	
	H 0.656624 2.328738 -0.031416	H 0.415425 1.216419 -2.205644	
	H 1.545068 1.151902 -0.726529	H 0.535720 2.223417 -0.934561	
	H 0.364535 -1.791980 -1.714006	H 0.358309 -2.275299 -1.165929	
	H 1.550429 -1.097168 -0.799311	H 1.502496 -1.310735 -0.471121	
	H -2.584877 -0.600469 -1.965162	H -2.381866 0.764980 -2.099575	
	H -2.316532 0.995710 -2.010592	H -1.618495 -0.594946 -2.555694	
	H -3.032507 -1.304326 0.817448	H -3.141185 -0.978088 1.092819	
	H -1.768466 -1.924129 1.631670	H -1.842137 -1.359440 1.999784	
	H 2.909085 -0.046215 1.457856	H 2.986208 0.267016 1.435486	
	H 4.319919 0.866617 0.887912	H 4.354503 1.014091 0.586995	
	O 3.093693 0.021220 -0.577388	O 3.049755 -0.138667 -0.567712	
	H 4.324790 -0.911967 0.829441	H 4.368923 -0.729998 0.940008	
	H 3.781380 0.044082 -1.254411	H 3.701069 -0.288886 -1.264109	
	S=0	S=1	S=2
$(H_3N)_5CrO^{2+}$	Cr -0.678710 -0.001639 -0.204554	Cr -0.674799 0.011914 -0.115310	Cr 0.748825 0.004353 0.010088
	O -0.081302 -0.016511 -1.608438	O 0.110658 0.193465 -1.509334	O -0.087789 0.336523 -1.640949
	N -2.130148 1.486836 -0.603948	N -1.952660 1.635923 -0.639603	N 2.124261 -1.260513 -1.004242
	N -2.122620 -1.506110 -0.584019	N -2.098643 -1.336742 -0.913297	N 1.911679 1.678115 -0.538444
	N 0.614473 -1.475232 0.540247	N 0.592574 -1.586748 0.357198	N -0.638551 1.297938 0.905752
	N -1.536208 0.021271 1.940622	N -1.755692 -0.255195 1.865330	N 1.680097 -0.388398 1.917946
	N 0.616335 1.493207 0.497648	N 0.579368 1.343424 0.890364	N -0.603491 -1.577243 0.078170
	C 3.730524 -0.009791 -0.697951	C 3.676637 0.093186 -0.710203	C -3.666784 0.185699 -0.657999
	H 0.347877 2.011913 1.332277	H 0.348065 1.606645 1.846829	H -0.388303 -2.387338 0.658861
	H -2.117142 0.830780 2.158547	H -1.108935 -0.372143 2.645547	H 0.995922 -0.587264 2.648630
	H 0.344859 -1.968057 1.390018	H 0.366706 -2.231319 1.113177	H -0.431457 1.743176 1.798824
	H -1.948801 -2.403172 -0.131559	H -1.906989 -2.314829 -0.696094	H 1.803565 2.505751 0.048084
	H -2.094149 1.651057 -1.611230	H -1.579717 1.943559 -1.540150	H 1.880402 -1.127610 -1.988572
	H -1.954933 2.393778 -0.172174	H -1.935546 2.448099 -0.023333	H 2.028079 -2.259294 -0.821163
	H -3.101913 1.260593 -0.395072	H -2.939558 1.431245 -0.793582	H 3.121057 -1.062983 -0.919057
	H -3.097834 -1.280607 -0.391025	H -3.072847 -1.179104 -0.657206	H 2.917024 1.537947 -0.638399
	H -2.074179 -1.690866 -1.587224	H -2.050088 -1.264916 -1.930832	H 1.570226 1.943922 -1.467256
	H 0.735626 -2.191213 -0.176061	H 0.705331 -2.140685 -0.492421	H -0.815017 2.043687 0.231226
	H 1.557382 -1.086821 0.701953	H 1.535224 -1.206893 0.545873	H -1.552376 0.825943 1.006335
	H -2.113287 -0.786387 2.175152	H -2.349667 0.532296 2.125250	H 2.315311 -1.186626 1.916988
	H -0.796091 0.030635 2.642675	H -2.365267 -1.071643 1.910916	H 2.232405 0.393429 2.271817
	H 0.737949 2.186636 -0.240437	H 0.658168 2.210714 0.359265	H -0.710896 -1.922456 -0.878158
	H 1.558721 1.109089 0.670790	H 1.536751 0.946145 0.899699	H -1.545667 -1.247267 0.353283
	H 2.915051 -0.016328 -1.419650	H 2.859991 0.155704 -1.426175	H -2.831347 0.417876 -1.317472
	H 4.339978 0.877076 -0.855738	H 4.263573 1.007814 -0.749404	H -4.258074 -0.615340 -1.095569
	H 4.336495 -0.902406 -0.835497	H 4.305800 -0.759555 -0.954088	H -4.283803 1.072468 -0.532307
	O 3.132823 0.006331 0.611401	O 3.080966 -0.072818 0.592902	O -3.102958 -0.225654 0.602572
	H 3.825310 0.014015 1.283698	H 3.776721 -0.155979 1.256887	H -3.810146 -0.442948 1.222507
	S=1/2	S=3/2	S=5/2
$(H_3N)_5MnO^{2+}$	Mn -0.662781 0.018332 -0.126236	Mn -0.674209 0.000357 0.079048	Mn -0.739338 -0.041649 0.024360
	O 0.047167 0.343166 -1.480318	O 0.133818 0.014797 1.487176	O 0.203794 0.458344 -1.446921
	N -1.954655 1.648645 -0.432454	N -1.970728 -1.443313 0.830026	N -2.292446 1.665741 -0.342234
	N -2.082606 -1.233099 -0.967576	N -1.962256 1.471424 0.791391	N -1.772110 -1.205585 -1.437748
	N 0.576092 -1.570607 0.200476	N 0.568592 1.452591 -0.630806	N 0.709903 -1.754580 0.385967
	N -1.661559 -0.433040 1.808217	N -1.761266 -0.026668 -1.850877	N -1.800806 -0.606098 1.741073
	N 0.567199 1.249709 0.952484	N 0.569717 -1.467084 -0.597495	N 0.564547 1.350579 0.891852
	C 3.690557 0.160274 -0.694024	C 3.659953 0.006083 0.702681	C 3.739715 0.251664 -0.709489
	H 0.313523 1.449338 1.918600	H 0.334945 -1.976879 -1.448091	H 0.381088 1.642349 1.851609
	H -0.995170 -0.585886 2.565375	H -1.130745 -0.010280 -2.652644	H -1.186521 -0.761386 2.541350
	H 1.525710 -1.230644 0.424070	H 1.518035 1.049963 -0.735385	H 1.619357 -1.299776 0.543325
	H -1.920132 -2.227799 -0.811633	H -1.868626 2.382969 0.343381	H -1.735028 -2.213578 -1.284997
	H -2.944961 1.448127 -0.568775	H -2.972234 -1.280934 0.727708	H -3.285771 1.456623 -0.435519
	H -1.626220 2.060291 -1.307638	H -1.766172 -1.472002 1.830813	H -1.996563 2.030893 -1.248355
	H -1.919214 2.390356 0.266366	H -1.806760 -2.386120 0.476916	H -2.248111 2.457394 0.298687
	H -3.050100 -1.059319 -0.697081	H -2.960251 1.262103 0.784794	H -2.755265 -0.967243 -1.567235

	H -2.038019 -1.097250 -1.978570	H -1.687207 1.595016 1.767816	H -1.317495 -1.034846 -2.338217
	H 0.321057 -2.254924 0.910874	H 0.335822 1.938218 -1.495980	H 0.602364 -2.457023 1.115614
	H 0.674874 -2.076433 -0.679901	H 0.667304 2.153369 0.104297	H 0.829968 -2.270542 -0.484913
	H -2.272952 0.316144 2.133205	H -2.342496 -0.853592 -1.987352	H -2.488175 0.087650 2.036312
	H -2.245562 -1.269171 1.800237	H -2.388588 0.765421 -1.989681	H -2.315941 -1.478978 1.618443
	H 0.644190 2.149852 0.479608	H 0.674218 -2.146523 0.156621	H 0.575545 2.198164 0.323245
	H 1.525174 0.858402 0.957691	H 1.517496 -1.064784 -0.716574	H 1.530626 0.969601 0.857863
	H 2.878535 0.309877 -1.402781	H 2.845215 0.015693 1.423295	H 2.942931 0.304603 -1.447620
	H 4.288461 1.066630 -0.634197	H 4.266489 -0.884104 0.850865	H 4.305655 1.180357 -0.722901
	H 4.310035 -0.669301 -1.026557	H 4.271235 0.895817 0.832976	H 4.395882 -0.582741 -0.946391
	O 3.086366 -0.135891 0.580991	O 3.057490 -0.005486 -0.609493	O 3.112958 0.055263 0.573024
	H 3.777381 -0.287682 1.237743	H 3.748962 -0.011896 -1.283190	H 3.792970 0.010478 1.256451
	S=0	S=1	S=2
$(H_3N)_5FeO^{2+}$	Fe 0.678109 -0.000528 -0.086860	Fe -0.679380 0.000211 0.086133	Fe 0.677112 0.001074 -0.056490
	O -0.109876 -0.007576 -1.486323	O 0.104053 0.004739 1.484981	O -0.064159 -0.006167 -1.469860
	N 1.948883 -1.446226 -0.754794	N -1.954315 -1.435389 0.764924	N 2.108598 1.496122 -0.773911
	N 1.951254 1.435543 -0.770998	N -1.953260 1.441681 0.754761	N -0.637906 1.550425 0.602085
	N -0.554621 1.435373 0.585475	N 0.555642 1.429670 -0.594235	N -0.637740 -1.541608 0.619286
	N 1.706642 0.010241 1.801549	N -1.698420 -0.006319 -1.802556	N 1.611927 0.016042 1.834704
	N -0.555916 -1.427625 0.601451	N 0.555092 -1.434027 -0.584459	N 2.100194 -1.516525 -0.749439
	C -3.691818 -0.004925 -0.693008	C 3.697567 0.003434 0.693458	C -3.721154 -0.005840 -0.779998
	H -0.304763 -1.905418 1.465852	H 0.306374 -1.919340 -1.445424	H 1.948093 -2.445241 -0.356973
	H 1.062201 0.009901 2.592471	H -2.295685 -0.819914 -1.950142	H 2.224975 -0.783323 1.993257
	H -1.506826 1.050527 0.707732	H 1.507606 1.044475 -0.716095	H -0.714654 -2.240963 -0.118611
	H 1.844487 2.350285 -0.332990	H -1.839051 2.354873 0.315372	H -1.578477 1.129314 0.681842
	H 1.706840 -1.586719 -1.737209	H -1.732366 -1.552176 1.755012	H 2.002519 1.516027 -1.788548
	H 1.846858 -2.353651 -0.300732	H -1.837731 -2.352790 0.335032	H 3.098880 1.342290 -0.588292
	H 2.945088 -1.231061 -0.727146	H -2.951112 -1.227597 0.711740	H 1.909576 2.444105 -0.455357
	H 2.947636 1.222812 -0.732789	H -2.950049 1.232469 0.707331	H -0.456542 2.062513 1.463522
	H 1.715512 1.559335 -1.757180	H -1.728005 1.568410 1.742886	H -0.718376 2.240823 -0.143813
	H -0.303981 1.921542 1.445339	H 0.304094 1.911661 -1.456243	H -0.455763 -2.043439 1.486634
	H -0.646944 2.140995 -0.145082	H 0.649456 2.140033 0.131473	H -1.579806 -1.122904 0.693440
	H 2.313073 -0.796314 1.950327	H -2.303651 0.800943 -1.952372	H 2.184707 0.844302 1.995687
	H 2.304900 0.823923 1.944523	H -1.049291 -0.004313 -2.589638	H 0.930736 -0.001633 2.593735
	H -0.649939 -2.140586 -0.121749	H 0.646826 -2.141466 0.144333	H 3.094227 -1.324310 -0.632162
	H -1.507573 -1.040543 0.720943	H 1.507295 -1.048947 -0.705126	H 1.939985 -1.606699 -1.753094
	H -2.886614 -0.012068 -1.424690	H 2.894606 0.008826 1.427463	H -2.903942 -0.017218 -1.498231
	H -4.303199 -0.895187 -0.819751	H 4.306885 -0.886619 0.831147	H -4.324176 0.885694 -0.936179
	H -4.300719 0.884816 -0.834451	H 4.309298 0.893398 0.820566	O -3.127900 0.003707 0.532604
	O -3.074126 0.004970 0.610348	O 3.076178 -0.003453 -0.608116	H -4.333596 -0.893785 -0.918927
	H -3.757629 0.010401 1.292015	H 3.757960 -0.007607 -1.291499	H -3.825391 0.010048 1.199766
$(H_3N)_5FeO^{2+}$	S=3		
	Fe 0.715564 -0.003858 0.008766		
	O -0.425367 -0.795408 -1.261310		
	N 1.572663 0.718912 -1.890320		
	N -0.544500 1.744141 0.268963		
	N -0.592166 -1.068185 1.354670		
	N 2.233738 1.027328 1.288049		
	N 2.094094 -1.736369 0.034901		
	C -3.791982 -0.296467 -0.605708		
	H 2.256756 -2.158875 0.948525		
	H 3.191103 0.705864 1.148251		
	H -0.613243 -2.063064 1.132315		
	H -1.508948 1.374529 0.251143		
	H 0.809672 1.074274 -2.467750		
	H 1.968698 -0.053976 -2.425131		
	H 2.283996 1.448945 -1.861203		
	H -0.454313 2.285764 1.127191		
	H -0.483662 2.416539 -0.494540		
	H -0.433781 -0.997989 2.359455		
	H -1.546730 -0.714574 1.168163		
	H 2.257514 2.036075 1.139926		
	H 2.051087 0.904666 2.284763		
	H 3.018944 -1.588888 -0.368286		
	H 1.648965 -2.453428 -0.539307		
	H -3.045400 -0.653221 -1.309395		
	H -4.397533 0.472549 -1.079711		
	O -3.081385 0.246615 0.526252		
	H -4.424538 -1.125388 -0.296658		
	H -3.724139 0.558860 1.175195		
	S=1/2	S=3/2	S=5/2
$(H_3N)_5CoO^{2+}$	Co -0.709864 0.000083 0.017786	Co -0.684145 0.017549 0.057313	Co 0.678309 0.001677 -0.091134
	O 0.103714 0.004432 1.588737	O 0.158309 -0.438187 1.398766	O -0.142510 -0.013172 -1.634169
	N -1.895456 -1.416674 0.747533	N -2.186008 -1.685352 0.301749	N 2.100068 -1.531085 -0.726586
	N -1.893955 1.423088 0.737621	N -1.837900 1.174061 1.223480	N 2.116697 1.488207 -0.789591
	N 0.555148 1.407536 -0.503421	N 0.692171 1.738585 -0.291535	N -0.639879 1.542760 0.603229
	N -1.619387 -0.004568 -1.814493	N -1.709958 0.572022 -1.640113	N 1.678129 0.038475 1.906456
	N 0.552751 -1.413175 -0.493795	N 0.509748 -1.186529 -0.967903	N -0.637704 -1.530975 0.628769
	C 3.640127 0.005029 0.662542	C 3.745692 -0.302009 0.699938	C -3.803293 -0.005277 -0.709327
	H 0.685588 -1.986810 0.340566	H 0.500647 -2.115335 -0.547343	H -0.720353 -2.285495 -0.051661

	H -0.940719 -0.013031 -2.576136	H -2.355232 1.350653 -1.509363	H 2.200109 0.895502 2.085371
	H 1.491061 1.026798 -0.722739	H 1.615348 1.322012 -0.463710	H -1.583119 1.131364 0.689050
	H -1.879113 2.301076 0.218886	H -1.761392 2.166160 1.001111	H 1.854252 2.440032 -0.535917
	H -1.509852 -1.616945 1.674084	H -1.942127 -2.081413 1.209509	H 1.966369 -1.739103 -1.716190
	H -1.888795 -2.295827 0.230616	H -2.104668 -2.452897 -0.363888	H 1.981116 -2.416034 -0.235016
	H -2.878060 -1.179856 0.881441	H -3.182364 -1.475682 0.347392	H 3.082683 -1.283685 -0.619307
	H -2.878705 1.190573 0.863073	H -2.828602 0.936278 1.191695	H 3.074252 1.367900 -0.463008
	H -1.515836 1.624632 1.666932	H -1.545375 1.069383 2.196188	H 2.155609 1.476053 -1.808834
	H 0.287473 2.024882 -1.269150	H 0.539590 2.435356 -1.018506	H -0.424009 1.993992 1.489899
	H 0.691822 1.982892 0.329168	H 0.794388 2.256069 0.580150	H -0.730941 2.285825 -0.088608
	H -2.221200 -0.811843 -1.976707	H -1.075742 0.863517 -2.383710	H 1.001131 -0.028627 2.665593
	H -2.210607 0.808683 -1.985590	H -2.267449 -0.191667 -2.022277	H 2.339063 -0.721044 2.064940
	H 1.490451 -1.036021 -0.711930	H 1.487520 -0.842951 -0.894809	H -1.583398 -1.123630 0.705222
	H 0.285285 -2.032080 -1.258352	H 0.290438 -1.303731 -1.955735	H -0.421919 -1.967148 1.522964
	H 2.810401 0.005150 1.369369	H 2.959681 -0.368624 1.448561	H -3.021778 -0.009596 -1.466903
	H 4.248396 -0.881522 0.825286	H 4.290491 -1.242680 0.666414	H -4.415970 -0.896532 -0.824019
	H 4.240313 0.898924 0.814958	H 4.422953 0.507606 0.961574	H -4.417506 0.883351 -0.835528
	O 3.065914 -0.005241 -0.658838	O 3.104991 -0.038397 -0.563394	O -3.149421 0.003564 0.573086
	H 3.766941 -0.009062 -1.322542	H 3.776890 0.029327 -1.253065	H -3.815912 0.008449 1.271259
	S=0	S=1	S=2
$(H_3N)_5NiO^{2+}$	Ni -0.715332 0.023221 0.014917	Ni -0.718445 0.026370 0.010707	Ni -0.696105 -0.000252 -0.072824
	O 0.081910 -0.235937 -1.530917	O 0.082400 -0.447082 -1.485995	O 0.216668 -0.005971 -1.584051
	N -1.914165 1.326570 -0.989287	N -1.944556 1.145303 -1.107617	N -2.043772 1.504556 -0.809531
	N -2.098423 -1.571012 -0.523262	N -2.042344 -1.693489 -0.337482	N -2.040408 -1.516152 -0.792641
	N 0.562351 -1.366784 0.679158	N 0.561311 -1.172426 0.915122	N 0.615190 -1.503961 0.658394
	N -1.575831 0.311090 1.783587	N -1.588624 0.554311 1.731373	N -1.750600 0.011733 1.823483
	N 0.681488 1.587316 0.357131	N 0.681657 1.658409 0.028111	N 0.615595 1.508991 0.646232
	C 3.741602 -0.106424 -0.712458	C 3.738565 -0.320648 -0.650879	C 3.854473 -0.001917 -0.692628
	H 0.499314 2.335965 1.023453	H 0.492518 2.462993 0.623334	H 0.393076 1.891370 1.562909
	H -2.144024 1.157014 1.821308	H -2.231191 1.340981 1.644248	H -2.334476 0.835421 1.963086
	H 1.523542 -0.981018 0.728518	H 1.504115 -0.742530 0.931237	H 1.567764 -1.113119 0.705952
	H -2.039451 -2.430866 0.020734	H -1.886287 -2.486053 0.283833	H -1.930997 -2.405346 -0.307640
	H -1.727200 1.218973 -1.987955	H -1.877778 2.149438 -0.945290	H -1.903990 2.412678 -0.369493
	H -1.732491 2.304349 -0.765329	H -2.934476 0.905299 -1.074565	H -3.030451 1.281941 -0.689467
	H -2.914033 1.175671 -0.861739	H -1.616607 0.967558 -2.059294	H -1.907205 1.652179 -1.809261
	H -3.092965 -1.363066 -0.601393	H -3.046191 -1.519501 -0.318038	H -3.027054 -1.273998 -0.719269
	H -1.772338 -1.799319 -1.463213	H -1.828850 -2.028119 -1.277823	H -1.870759 -1.710254 -1.779280
	H 0.343776 -1.784907 1.582106	H 0.340889 -1.496389 1.855691	H 0.392587 -1.879320 1.577945
	H 0.592439 -2.124312 -0.003295	H 0.645954 -1.988560 0.309551	H 0.666183 -2.299964 0.024847
	H -2.188787 -0.457785 2.053471	H -2.126797 -0.206720 2.145423	H -2.368170 -0.787471 1.960291
	H -0.882081 0.406184 2.525143	H -0.897403 0.830425 2.428697	H -1.107618 -0.002807 2.614214
	H 0.819989 2.017691 -0.556407	H 0.768640 2.009833 -0.924809	H 0.667715 2.300040 0.006619
	H 1.591588 1.172660 0.598219	H 1.612797 1.299761 0.280836	H 1.567838 1.117694 0.697082
	H 2.936740 -0.073806 -1.444781	H 2.932073 -0.521792 -1.354034	H 3.101896 -0.002966 -1.478515
	H 4.391939 0.753439 -0.855487	H 4.377726 0.462523 -1.051791	H 4.472742 0.887194 -0.793043
	H 4.309163 -1.024642 -0.844648	H 4.317292 -1.229075 -0.500618	H 4.470777 -0.892876 -0.788697
	O 3.126508 -0.068231 0.589177	O 3.124941 0.103686 0.581201	O 3.155057 0.001959 0.565321
	H 3.809292 -0.090794 1.271002	H 3.807810 0.277882 1.240693	H 3.798409 0.004112 1.284848

Table S7. Partially optimized geometries (Cartesian coordinates in Å) for the encounter complex between methanol and $(NH_3)_5MO^{2+}$ species.

	S=0	S=1	
$(H_3N)_5TiO^{2+}$	Ti 0.789660 -0.019178 -0.186168	Ti -0.872286 0.002608 -0.023720	
	O 0.022097 -0.065771 -1.567160	O 0.158821 0.182295 -1.539978	
	N 2.238485 -1.627192 -0.740652	N 0.397057 1.712537 0.650035	
	N 2.348315 1.449501 -0.843540	N -2.229599 -0.342441 1.810257	
	N -0.410278 1.688999 0.571927	N 0.540008 -1.421666 0.984366	
	N 1.911935 0.012976 2.017851	N -1.731934 -1.635650 -1.296697	
	N -0.565486 -1.510040 0.749727	N -2.451761 1.438639 -0.741309	
	C -3.508683 0.378522 0.044346	C 3.488627 0.387079 0.057360	
	H -0.346354 -1.938572 1.648139	O 4.644633 -0.370488 -0.169886	
	H 1.272121 0.202309 2.789529	H 3.692602 1.455919 0.036902	
	H -0.164921 2.170337 1.436216	H 3.178201 0.090970 1.061566	
	H 2.338401 2.351361 -0.366114	H 2.734358 0.142706 -0.694974	
	H 1.852309 -2.564521 -0.617683	H 0.353826 2.472545 -0.030889	
	H 3.157243 -1.651180 -0.299077	H 0.216554 2.143625 1.557560	
	H 2.398287 -1.547513 -1.746971	H -2.335736 -1.329791 2.047447	
	H 3.318679 1.137264 -0.801300	H -3.180173 0.014895 1.711465	
	H 2.162229 1.653449 -1.827481	H 1.357598 -1.429522 0.368767	
	H -0.444003 2.399984 -0.161347	H 0.254759 -2.396493 1.078967	
	H -1.387261 1.394988 0.668796	H -1.974409 -1.260944 -2.215363	
	H 2.361721 -0.868057 2.266897	H -2.564046 -2.127526 -0.968738	
	H 2.646343 0.714493 2.112144	H -3.374073 1.058431 -0.954994	
	H -0.710837 -2.273069 0.085747	H -2.626341 2.268643 -0.174151	
	H -1.495911 -1.092693 0.845603	H -2.090557 1.787156 -1.631925	
	H -2.732012 0.075732 -0.661738	H -1.036502 -2.358018 -1.487608	
	H -3.245212 0.155705 1.082216	H 0.899003 -1.153030 1.900984	
	O -4.662517 -0.385912 -0.169898	H -1.870140 0.092699 2.661829	
	H -3.701619 1.444586 -0.056991	H 1.380470 1.426997 0.660947	
	H -5.137060 -0.100058 -0.957696	H 5.149106 -0.033508 -0.917760	
	S=1/2	S=3/2	
$(H_3N)_5VO^{2+}$	V -0.764600 -0.012490 -0.175739	V 0.864083 0.011789 -0.031310	
	O 0.070287 -0.060897 -1.478305	O -0.131006 0.277263 -1.548755	
	N -2.219627 1.413518 -0.928844	N 1.845124 -1.434144 -1.321788	
	N -2.142481 0.026982 1.766254	N 2.344377 1.516435 -0.562938	
	N 0.369122 1.618699 0.680167	N -0.464589 1.544345 0.718071	
	N 0.487447 -1.362569 0.959974	N 2.079981 -0.308866 1.816832	
	N -2.015251 -1.678969 -0.796291	N -0.440058 -1.536873 0.748849	
	C 3.472681 0.345704 0.037833	C -3.470512 -0.307199 0.113378	
	H 3.643914 1.420007 -0.000818	H -1.033370 -1.259283 1.532142	
	H 3.177684 0.056496 1.049001	H 1.514747 -0.535762 2.635555	
	O 4.653861 -0.375590 -0.178488	H -1.424460 1.188603 0.713115	
	H 2.726726 0.063505 -0.708425	H 2.313226 2.381265 -0.022092	
	H -1.848508 1.780557 -1.807142	H 1.407939 -1.337116 -2.242904	
	H -3.138510 1.034890 -1.159034	H 1.729172 -2.416019 -1.070107	
	H -2.003290 0.833711 2.374743	H 2.845635 -1.311617 -1.479315	
	H -2.021303 -0.782971 2.374527	H 3.323777 1.230799 -0.564762	
	H 1.359992 1.375329 0.582858	H 2.129290 1.787688 -1.525069	
	H 0.231943 1.879838 1.655837	H -0.308455 1.941630 1.644640	
	H 1.377271 -1.416447 0.458190	H -0.481429 2.332361 0.067984	
	H 0.156513 -2.323701 1.046211	H 2.754594 -1.070596 1.736227	
	H -1.408366 -2.446875 -1.087715	H 2.628200 0.505920 2.093693	
	H -2.689281 -2.077200 -0.143495	H -0.019712 -2.426312 1.018744	
	H -2.539906 -1.443773 -1.639870	H -1.094338 -1.743643 -0.010418	
	H 0.720802 -1.064609 1.907347	H -2.707714 -0.038269 -0.620887	
	H 0.262045 2.478390 0.140455	H -3.616552 -1.385610 0.120413	
	H -3.137440 0.041801 1.541443	O -4.612492 0.367963 -0.330323	
	H -2.401270 2.223045 -0.335499	H -3.212868 0.027721 1.120973	
	H 5.133050 -0.050681 -0.948075	H -5.404387 0.072197 0.131369	
	S=0	S=1	S=2
$(H_3N)_5CrO^{2+}$	Cr -0.771932 0.018659 -0.181529	Cr 0.802451 0.014219 -0.101201	Cr -0.881557 -0.013630 0.011529
	O 0.019527 0.086321 -1.479338	O -0.110519 0.115388 -1.428839	O 0.176897 0.020976 -1.537487
	N 0.471820 1.369708 0.859511	N 2.048157 -1.392898 -1.089490	N -2.178322 1.384667 -0.914519
	N -2.068261 1.621960 -0.660495	N 2.029647 1.618804 -0.725384	N -1.832068 -1.578751 -1.028583
	N -2.212026 -1.350571 -0.905434	N -0.455576 1.402903 0.883026	N 0.466901 -1.405217 0.857863
	N -1.960811 -0.113668 1.794165	N 2.137697 -0.152498 1.722840	N -2.099465 -0.051592 1.796535
	N 0.359995 -1.573852 0.609044	N -0.372027 -1.529384 0.709237	N 0.379135 1.562541 0.576826
	C 3.474968 -0.333370 0.045981	C -3.458415 -0.325832 0.073125	C 3.451450 0.355512 0.102325
	H 0.151793 -2.480838 0.191532	H -0.238695 -1.787028 1.686638	H 0.062352 2.232955 1.277869
	H -2.369284 -1.034548 1.955824	H 1.833515 0.401439 2.523964	H -1.553939 -0.087115 2.658427
	H -2.227774 -2.266372 -0.457134	H -0.880678 1.096448 1.758415	H 0.923147 -1.131984 1.728740
	H -3.069604 1.429608 -0.655606	H 2.517692 2.154573 -0.007980	H -1.930387 -2.472998 -0.547104
	H 0.949266 1.928504 0.149902	H 1.631778 -1.476100 -2.019703	H -1.827706 1.440864 -1.874014
	H 1.223569 0.924627 1.387538	H 2.072314 -2.334351 -0.697660	H -2.142980 2.336137 -0.547425
	H 0.029705 2.033921 1.494305	H 3.026070 -1.139303 -1.229678	H -3.170764 1.156392 -0.975167
	H -1.948055 2.474281 -0.113569	H 2.738320 1.344226 -1.406452	H -2.754554 -1.370278 -1.411468

	H -1.844292 1.882486 -1.622602	H 1.430930 2.276322 -1.228291	H -1.230053 -1.757537 -1.838919
	H -3.181892 -1.036140 -0.920175	H -0.063641 2.324956 1.074661	H 0.125378 -2.353217 1.016475
	H -1.965183 -1.526540 -1.881061	H -1.226172 1.544526 0.225002	H 1.210600 -1.475675 0.157851
	H -1.397712 0.071817 2.624636	H 2.220476 -1.106097 2.076229	H -2.708053 0.761275 1.898045
	H -2.745359 0.534869 1.862711	H 3.100662 0.138099 1.551542	H -2.723652 -0.858047 1.844167
	H 0.340430 -1.717756 1.618069	H -0.293344 -2.391939 0.169311	H 0.577819 2.094369 -0.274851
	H 1.337992 -1.387976 0.366966	H -1.354129 -1.251367 0.606471	H 1.297898 1.229537 0.881521
	H 2.702693 0.086452 -0.602098	H -2.724232 -0.072100 -0.694544	H 2.712726 0.125528 -0.668345
	H 3.263446 -0.160821 1.103018	H -3.604659 -1.404012 0.111862	H 3.614058 1.430331 0.153817
	O 4.681379 0.345117 -0.167194	O -4.620252 0.333084 -0.347381	O 4.640438 -0.336100 -0.159383
	H 3.579540 -1.401168 -0.139175	H -3.168038 0.044843 1.058750	H 3.151418 -0.020063 1.084006
	H 5.105013 0.071456 -0.987537	H -5.395628 0.034349 0.139687	H 5.123417 0.053030 -0.896163
	S=1/2	S=3/2	S=5/2
$(H_3N)_5MnO^{2+}$	Mn 0.785947 0.014788 -0.125171	Mn -0.816104 -0.010840 -0.078104	Mn 0.884184 0.035659 0.023650
	O -0.128341 0.132431 -1.381317	O 0.000550 -0.042953 -1.478445	O -0.119366 -0.431948 -1.412120
	N 1.895570 -1.472556 -1.029362	N 0.358794 1.564512 0.527270	N -0.369191 -1.447130 0.894000
	N 2.115959 1.473422 -0.728169	N -1.897112 0.023771 1.855771	N 2.035210 0.597821 1.686404
	N -0.386619 1.483608 0.774565	N 0.504183 -1.387079 0.697143	N -0.612408 1.661618 0.626388
	N 2.136259 -0.147046 1.641372	N -2.023678 -1.555055 -0.746612	N 1.716554 1.303547 -1.483314
	N -0.373840 -1.389393 0.827154	N -2.170266 1.338720 -0.876784	N 2.448529 -1.572543 -0.487749
	C -3.462596 -0.313506 0.084624	C 3.485322 0.366923 0.059243	C -3.496374 -0.421870 0.096592
	H -0.555813 -1.198356 1.812590	H 3.222175 0.158710 1.100544	H -3.201982 0.090570 1.015391
	H 2.049574 0.624844 2.303018	O 4.659886 -0.372022 -0.130496	O -4.700389 0.201836 -0.255798
	H -1.353297 1.148721 0.770045	H 3.654199 1.434802 -0.063674	H -3.635573 -1.483075 0.293665
	H 2.176006 2.277798 -0.103759	H 2.723027 0.027224 -0.646055	H -2.764079 -0.283633 -0.701567
	H 1.240027 -1.967145 -1.637184	H 1.340060 1.269962 0.499490	H -1.344909 -1.139057 0.834547
	H 2.341362 -2.173565 -0.437966	H 0.304856 2.289338 -0.189935	H -0.321749 -2.295742 0.327704
	H 2.622346 -1.129495 -1.657614	H -2.436651 -0.821542 2.043567	H 2.315527 1.579265 1.655952
	H 3.076828 1.169122 -0.884042	H -2.565280 0.788777 1.951132	H 2.896875 0.060587 1.787908
	H 1.785668 1.832440 -1.625713	H 0.757456 -1.998937 -0.081275	H -1.363862 1.547882 -0.056545
	H -0.190844 1.806713 1.721721	H 0.214865 -1.972828 1.480375	H -0.322106 2.637457 0.568649
	H -0.405072 2.312249 0.179411	H -1.945456 -1.526250 -1.765491	H 1.931283 0.733969 -2.303757
	H 1.996737 -0.989209 2.200091	H -3.015825 -1.528992 -0.510681	H 2.561367 1.832515 -1.264375
	H 3.123800 -0.165219 1.385633	H -3.127899 1.003463 -0.983613	H 3.386947 -1.265991 -0.743781
	H -0.039055 -2.351661 0.778428	H -2.246002 2.236112 -0.397079	H 2.589279 -2.326000 0.185073
	H -1.289494 -1.381527 0.367534	H -1.821148 1.530071 -1.818536	H 2.065000 -2.016338 -1.323564
	H -2.722173 0.022616 -0.645139	H -1.698449 -2.483300 -0.474573	H 1.019521 1.978226 -1.801095
	H -3.550522 -1.398974 0.061755	H 1.387782 -0.947430 0.965565	H -1.058819 1.569870 1.538830
	O -4.691777 0.301769 -0.179934	H -1.271653 0.118171 2.656514	H 1.530645 0.490240 2.567617
	H -3.225159 0.013511 1.099101	H 0.197734 2.012732 1.429068	H -0.200441 -1.728713 1.860106
	H -5.104366 -0.051374 -0.975297	H 5.129928 -0.094896 -0.924178	H -5.182095 -0.304571 -0.918316
	S=0	S=1	S=2
$(H_3N)_5FeO^{2+}$	Fe -0.808695 -0.009381 -0.079822	Fe -0.810775 -0.009965 -0.080039	Fe -0.817017 0.007758 -0.059048
	O 0.045624 -0.050308 -1.439667	O 0.039472 -0.062498 -1.437257	O 0.068081 0.002607 -1.386330
	N -2.101984 1.321605 -0.903837	N -2.102191 1.318007 -0.910107	N 0.551598 1.410263 0.919769
	N -1.970864 -1.536570 -0.748081	N -1.976838 -1.537674 -0.737423	N -2.028072 -0.011982 1.671934
	N 0.453397 -1.348449 0.758097	N 0.450091 -1.345216 0.764079	N 0.389316 -1.596120 0.698219
	N -1.927249 0.046340 1.755622	N -1.925949 0.061979 1.753290	N -2.233888 -1.402632 -0.950041
	N 0.343629 1.516124 0.573329	N 0.346683 1.513516 0.566078	N -1.917458 1.686169 -0.848891
	C 3.475945 0.325296 0.072610	C 3.482048 0.321150 0.068212	C 3.504172 -0.361866 0.005989
	H 0.260096 1.810389 1.546164	H 0.301501 1.772444 1.551477	H 3.223609 -0.025580 1.006550
	H -1.331028 0.032674 2.583727	H -1.328804 0.042305 2.580621	H 3.663883 -1.438313 0.010718
	H 1.274797 -0.916736 1.184834	H 1.282489 -0.910877 1.166541	O 4.689189 0.337146 -0.256702
	H -1.711906 -2.457695 -0.394266	H -1.710390 -2.458906 -0.389534	H 2.751120 -0.101505 -0.740898
	H -1.700117 1.568365 -1.810586	H -1.700600 1.564718 -1.816809	H 1.353884 1.494973 0.293163
	H -2.232437 2.194748 -0.392707	H -2.236130 2.191780 -0.400986	H 0.200376 2.356326 1.067178
	H -3.040073 0.970282 -1.095551	H -3.039159 0.964364 -1.103353	H -2.117733 -0.942068 2.081276
	H -2.976651 -1.454103 -0.599909	H -2.980839 -1.458924 -0.575297	H -2.983122 0.296803 1.489619
	H -1.820578 -1.577446 -1.757971	H -1.841416 -1.576003 -1.749403	H 0.319255 -2.402949 0.077467
	H 0.067863 -1.990901 1.450165	H 0.070626 -1.968272 1.476949	H 0.272089 -1.944852 1.648693
	H 0.824914 -1.911118 -0.009686	H 0.805292 -1.929877 0.005316	H -1.916532 -1.555194 -1.908246
	H -2.508933 0.877857 1.861531	H -2.496783 0.901058 1.858400	H -3.205448 -1.099341 -1.014282
	H -2.565889 -0.739805 1.878760	H -2.573651 -0.716401 1.878303	H -2.534072 1.396146 -1.608324
	H 0.205194 2.349452 0.001374	H 0.179184 2.366825 0.032898	H -2.476322 2.273778 -0.231144
	H 1.322928 1.258748 0.416020	H 1.320467 1.268541 0.361727	H -1.229459 2.294364 -1.294783
	H 2.715977 -0.061064 -0.610726	H 2.726203 -0.075698 -0.613565	H -2.265951 -2.323489 -0.512679
	H 3.581423 1.401388 -0.053678	H 3.579531 1.397294 -0.064483	H 1.362452 -1.288898 0.609882
	O 4.686820 -0.339536 -0.155161	O 4.698864 -0.334865 -0.153670	H -1.682818 0.587959 2.421305
	H 3.252314 0.097648 1.117594	H 3.258503 0.098265 1.114125	H 0.929957 1.109651 1.818078
	H 5.114439 -0.035153 -0.962657	H 5.122574 -0.035826 -0.965195	H 5.163624 -0.036232 -1.006945
$(H_3N)_5FeO^{2+}$	S=3		
	Fe -0.856550 -0.002292 -0.003444		
	O 0.230201 0.268406 -1.512991		
	N -2.343406 1.495809 -0.646264		
	N -1.654050 -1.500076 -1.397184		
	N 0.520609 -1.446135 0.913356		
	N -2.252694 -0.458149 1.679594		
	N 0.409574 1.601187 0.753303		
	C 3.527356 0.355827 0.091494		
	H 0.262500 1.949618 1.700893		

	H -2.096419 0.138547 2.493134		
	H 0.745392 -1.298071 1.897239		
	H -0.890013 -2.097720 -1.715444		
	H -1.959222 1.941418 -1.480843		
	H -2.541532 2.247026 0.014650		
	H -3.254202 1.128409 -0.921920		
	H -2.399919 -2.120972 -1.082868		
	H -1.994360 -1.039784 -2.242213		
	H 0.252300 -2.427242 0.836417		
	H 1.401681 -1.352559 0.402998		
	H -3.237529 -0.335583 1.444263		
	H -2.172588 -1.413105 2.028975		
	H 0.352303 2.410797 0.134397		
	H 1.385517 1.294949 0.699457		
	H 2.790414 0.114058 -0.677925		
	H 3.686223 1.432001 0.123966		
	O 4.717647 -0.336616 -0.163917		
	H 3.228086 -0.002234 1.079237		
	H 5.204522 0.052312 -0.898194		
	S=1/2	S=3/2	S=5/2
$(H_3N)_5CoO^{2+}$	Co -0.842584 -0.009045 -0.012634	Co 0.809833 0.013215 -0.055687	Co -0.797342 -0.012694 -0.077923
	O 0.159034 -0.072069 -1.469612	O -0.073362 -0.424371 -1.374058	O 0.141732 -0.045852 -1.559485
	N -1.887240 1.389747 -0.951036	N 2.329793 -1.628312 -0.382646	N -2.200446 1.439422 -0.904958
	N -1.941652 -1.441906 -0.821301	N 1.859026 1.196638 -1.287063	N -2.108985 -1.592503 -0.810824
	N 0.383189 -1.380842 0.700418	N -0.589366 1.700445 0.464019	N 0.534813 -1.477099 0.797563
	N -2.006592 0.038778 1.672620	N 1.889201 0.599704 1.601435	N -1.945413 0.004027 1.827958
	N 0.356188 1.418218 0.640291	N -0.332416 -1.252615 0.983842	N 0.415643 1.626910 0.650209
	C 3.438706 0.283366 0.126707	C -3.469799 -0.391661 0.116532	C 3.539265 0.348111 0.054602
	H 0.981990 1.589702 -0.152672	H -0.300470 -2.174079 0.546088	H 0.171080 2.044230 1.546829
	H -1.555994 0.493024 2.467277	H 1.345180 0.562370 2.463409	H -1.350119 0.165157 2.639964
	H 1.337855 -1.009617 0.715385	H -1.058169 1.632732 1.366789	H 1.469315 -1.074243 0.893201
	H -1.962883 -2.323173 -0.307768	H 1.307959 2.004571 -1.577562	H -1.837068 -2.523815 -0.496766
	H -1.248031 1.784815 -1.645679	H 1.994125 -2.126312 -1.207577	H -1.929216 1.685146 -1.857521
	H -2.227081 2.164145 -0.380365	H 2.416882 -2.332167 0.349783	H -2.240944 2.317208 -0.387843
	H -2.693049 1.056717 -1.480441	H 3.284973 -1.346473 -0.600841	H -3.163875 1.111902 -0.965445
	H -2.916596 -1.222304 -1.024905	H 2.749021 1.549521 -0.936668	H -3.092926 -1.496100 -0.563167
	H -1.477484 -1.627549 -1.715162	H 2.061844 0.679883 -2.144088	H -2.070866 -1.626774 -1.830016
	H 0.196514 -1.770992 1.624278	H -0.236177 2.655453 0.414946	H 0.289260 -1.866012 1.706611
	H 0.429179 -2.160265 0.042415	H -1.331186 1.634186 -0.234068	H 0.660625 -2.268358 0.165809
	H -2.892193 0.529470 1.546793	H 2.719449 0.030948 1.768212	H -2.669083 0.720377 1.878404
	H -2.265638 -0.890497 2.004744	H 2.215354 1.564078 1.536662	H -2.423905 -0.876217 2.017272
	H 0.958237 1.173992 1.427766	H -1.310914 -0.954642 0.922761	H 0.420936 2.386244 -0.031160
	H -0.082386 2.306087 0.884616	H -0.110033 -1.384140 1.969813	H 1.393714 1.335590 0.717806
	H 2.716367 -0.021922 -0.634619	H -2.746044 -0.287265 -0.694451	H 2.762059 0.016369 -0.638472
	H 3.511533 1.368764 0.157519	H -3.554576 -1.436124 0.411030	H 3.687374 1.421493 -0.044218
	O 4.676770 -0.310075 -0.137188	O -4.704886 0.130107 -0.286691	O 4.716109 -0.366290 -0.193174
	H 3.184907 -0.092901 1.120942	H -3.205263 0.220655 0.981435	H 3.302012 0.105246 1.093275
	H 5.108616 0.085713 -0.901786	H -5.156338 -0.456930 -0.902423	H 5.182243 -0.031535 -0.966636
	S=0	S=1	S=2
$(H_3N)_5NiO^{2+}$	Ni -0.849875 -0.023893 0.017304	Ni 0.856016 -0.026945 0.018994	Ni -0.801434 -0.008272 -0.058023
	O 0.124166 0.290175 -1.413983	O -0.051873 0.211065 -1.471741	O 0.201165 -0.042464 -1.524899
	N -2.158469 1.567863 -0.610562	N 2.048845 -1.258941 -1.015620	N -2.166910 1.375152 -0.969807
	N -1.873714 -1.278601 -1.213538	N 2.058982 1.649119 -0.690380	N -1.993079 -1.629188 -0.814079
	N 0.519700 -1.583926 0.536692	N -0.409637 1.250863 0.882602	N 0.520549 -1.416048 0.864048
	N -1.931434 -0.401099 1.647807	N 1.862181 -0.287537 1.726070	N -1.976421 0.038914 1.756895
	N 0.372822 1.339306 0.876976	N -0.523466 -1.657519 0.336605	N 0.389665 1.622899 0.647592
	C 3.501113 0.341129 0.105009	C -3.516545 0.309400 0.146280	C 3.516251 0.335831 0.045673
	H 0.191468 1.599936 1.845715	H -0.417391 -2.257462 1.153737	H 0.214428 1.926792 1.603747
	H -1.366924 -0.380834 2.497152	H 1.237902 -0.446039 2.517156	H -1.393844 0.096306 2.591682
	H 1.068915 -1.434772 1.382661	H -1.106806 0.815250 1.487754	H 1.341007 -0.962899 1.266327
	H -1.402124 -2.174622 -1.333784	H 1.747534 2.563436 -0.364932	H -1.705981 -2.542508 -0.464823
	H -1.702715 1.902057 -1.461109	H 1.641966 -1.256758 -1.953612	H -1.873841 1.611782 -1.917898
	H -2.245591 2.371797 0.010509	H 2.066336 -2.225977 -0.692288	H -2.242340 2.259838 -0.469663
	H -3.111441 1.316533 -0.871533	H 3.020152 -0.961918 -1.105138	H -3.117668 1.018271 -1.052959
	H -2.840225 -1.481630 -0.959846	H 3.062908 1.616554 -0.516133	H -2.988073 -1.550485 -0.609145
	H -1.893376 -0.851625 -2.141356	H 1.941042 1.669341 -1.704303	H -1.916933 -1.686249 -1.829770
	H 0.161362 -2.535895 0.603835	H 0.007315 2.016627 1.410898	H 0.104694 -1.979200 1.604322
	H 1.182526 -1.564225 -0.240475	H -0.928810 1.648065 0.095947	H 0.889603 -2.070123 0.173621
	H -2.691337 0.263823 1.791483	H 2.501149 -1.082156 1.709648	H -2.613015 0.833026 1.815640
	H -2.359793 -1.326631 1.626763	H 2.428094 0.525171 1.971118	H -2.559726 -0.786228 1.892265
	H 0.348388 2.198046 0.326295	H -0.511441 -2.269219 -0.479296	H 0.285633 2.449430 0.059909
	H 1.340863 1.008992 0.820418	H -1.471650 -1.277024 0.365022	H 1.380380 1.376717 0.592422
	H 2.776577 0.195496 -0.698907	H -2.785969 0.165432 -0.653057	H 2.730511 -0.096351 -0.578404
	H 3.604134 1.400848 0.331074	O -4.764676 -0.172617 -0.261737	H 3.616126 1.399040 -0.163691
	O 4.727393 -0.226717 -0.260973	H -3.562744 1.360870 0.423163	O 4.710753 -0.352950 -0.185570
	H 3.225348 -0.208384 1.007786	H -3.287213 -0.291695 1.029302	H 3.323411 0.184098 1.109682
	H 5.188973 0.310833 -0.913214	H -5.177500 0.406084 -0.911597	H 5.156331 -0.043414 -0.981448

Table S8. Optimized geometries (Cartesian coordinates in Å) for the transitions states of the reaction between methanol and $(NH_3)_5MO^{2+}$ species.

	S=1/2	S=3/2	
$(H_3N)_5VO^{2+}$	V -0.560056 -0.028913 0.040168	V -0.614791 0.012771 -0.023553	
	O 0.605583 -0.611808 1.144934	O 0.546776 0.922032 -1.099820	
	N -1.764271 0.368190 1.838170	N -1.726705 -0.119006 -1.894618	
	N 0.252020 1.982290 0.003921	N 0.405899 -1.849895 -0.410895	
	N 0.653139 -0.454628 -1.718391	N 0.643568 0.335322 1.717471	
	N -2.272489 0.826136 -1.196689	N -2.087796 -1.183934 1.160572	
	N -1.557704 -1.956021 -0.219094	N -1.753372 1.794909 0.518353	
	C 3.042638 -0.709345 0.459404	C 3.039026 0.758385 -0.365509	
	H -1.702403 -2.282681 -1.174036	H -1.723424 2.078040 1.497642	
	H -2.046187 1.014386 -2.173017	H -2.786781 -0.614511 1.637597	
	H 0.911797 -1.440917 -1.750146	H 0.901783 1.320882 1.774899	
	H 0.062256 2.562947 -0.811651	H 1.400542 -1.613971 -0.290180	
	H -1.255940 1.013498 2.442462	H -1.116288 0.365992 -2.558145	
	H -1.790770 -0.499210 2.374753	H -2.633352 0.343525 -1.951637	
	H -2.722185 0.712044 1.791936	H -1.875325 -1.051525 -2.279477	
	H 0.039301 2.558745 0.817028	H 0.222781 -2.662910 0.175254	
	H 1.263936 1.826795 0.024751	H 0.315820 -2.168477 -1.375037	
	H 0.282819 -0.237272 -2.642788	H 0.294878 0.080574 2.640655	
	H 1.540942 0.049781 -1.636860	H 1.531778 -0.162293 1.588924	
	H -3.096498 0.226301 -1.223905	H -2.620926 -1.829258 0.577275	
	H -2.601517 1.714852 -0.819742	H -1.667122 -1.758258 1.890936	
	H -2.462738 -2.044358 0.242512	H -2.738140 1.818298 0.254688	
	H -0.952960 -2.645162 0.230227	H -1.306070 2.551029 -0.004433	
	H 1.760757 -0.773480 0.918043	H 1.941629 0.975769 -0.714693	
	H 3.659632 -0.785554 1.348034	H 3.639682 0.813640 -1.270172	
	O 3.102834 0.497467 -0.210233	O 3.023923 -0.520375 0.220058	
	H 3.092961 -1.566166 -0.208793	H 3.289053 1.545308 0.347278	
	H 3.800063 1.069484 0.137866	H 3.898761 -0.930922 0.195314	
	S=0	S=1	S=2
$(H_3N)_5CrO^{2+}$	Cr 0.578921 -0.016422 -0.026524	Cr -0.596381 0.000120 -0.002564	Cr -0.637727 -0.020095 0.000005
	O -0.567165 -0.594341 -1.160447	O 0.547125 0.801781 -1.099574	O 0.548080 1.426934 0.000730
	N 1.512394 -1.938813 -0.011818	N -1.571297 1.888710 0.173913	N -1.683116 1.078861 1.479766
	N 1.804810 0.592654 -1.661562	N -1.740783 -0.419256 -1.753100	N -1.682930 1.079910 -1.479127
	N -0.330671 1.892177 0.042512	N 0.429228 -1.816157 -0.236866	N 0.519247 -0.924068 -1.499159
	N 2.187073 0.729484 1.292003	N -2.143633 -0.941481 1.237993	N -2.066261 -1.648486 -0.000760
	N -0.629136 -0.620176 1.603852	N 0.581396 0.458566 1.680038	N 0.519031 -0.925151 1.498671
	C -3.014332 -0.624356 -0.525475	C 2.994068 0.644036 -0.505487	C 3.031239 0.803481 0.000006
	H -0.229785 -0.587151 2.541281	H 0.211021 0.283373 2.613313	H 0.220226 -1.803103 1.921335
	H 2.912885 0.040178 1.487187	H -1.765861 -1.532778 1.978507	H -1.628963 -2.570281 -0.000712
	H -0.061446 2.557064 0.766191	H 0.154855 -2.639292 0.297325	H 0.220359 -1.801640 -1.922551
	H 2.030526 1.584047 -1.734709	H -1.945379 -1.398383 -1.950615	H -1.724424 0.667292 -2.410772
	H 0.948286 -2.477824 -0.670790	H -0.992961 2.486844 -0.420143	H -1.103973 1.919612 1.561259
	H 1.499377 -2.455115 0.867190	H -1.582980 2.318354 1.098512	H -1.724925 0.665478 2.411057
	H 2.472488 -1.997774 -0.349019	H -2.525896 1.968381 -0.174631	H -2.631700 1.392245 1.276713
	H 2.689038 0.101860 -1.788644	H -2.626888 0.072272 -1.864366	H -2.631625 1.392953 -1.276067
	H 1.237336 0.367522 -2.480588	H -1.132647 -0.087591 -2.504885	H -1.103914 1.920827 -1.559781
	H -0.252013 2.386856 -0.845242	H 0.429390 -2.090023 -1.219061	H 0.643534 -0.256863 -2.260885
	H -1.331396 1.705542 0.160750	H 1.414065 -1.617936 -0.018579	H 1.468322 -1.071047 -1.127530
	H 2.679641 1.532983 0.902436	H -2.749527 -0.272423 1.712894	H -2.685717 -1.644107 0.809900
	H 1.858355 1.037022 2.207434	H -2.774155 -1.545407 0.710827	H -2.685088 -1.643892 -0.811898
	H -0.929602 -1.584161 1.458366	H 0.818127 1.450662 1.642369	H 0.643106 -0.258517 2.260930
	H -1.494728 -0.074112 1.634963	H 1.483878 -0.026092 1.617545	H 1.468187 -1.071657 1.127059
	H -1.785883 -0.673761 -0.938510	H 1.784029 0.760423 -0.880432	H 1.882798 1.158997 0.000337
	H -3.120083 -1.559814 0.021480	H 3.182716 1.580945 0.016158	H 3.456777 1.225746 0.907968
	H -3.606540 -0.573071 -1.433719	H 3.554944 0.532399 -1.428707	H 3.456293 1.225773 -0.908174
	O -3.096512 0.488305 0.295751	O 3.033653 -0.472755 0.333684	O 3.006621 -0.606263 -0.000018
	H -3.826454 1.070960 0.046022	H 3.881420 -0.935705 0.281698	H 3.899340 -0.979235 0.000214
	S=1/2	S=3/2	S=5/2
$(H_3N)_5MnO^{2+}$	Mn -0.586386 -0.006278 0.017715	Mn 0.592857 0.001048 -0.003268	Mn -0.623893 0.031260 0.037398
	O 0.522120 -0.573742 1.209780	O -0.522816 -1.175581 -0.693696	O 0.411357 -0.566767 -1.325084
	N -1.553405 -1.849665 0.121893	N 1.580353 -1.639374 0.823060	N -1.716276 1.137132 -1.452606
	N -1.749972 0.715622 1.589413	N 1.672516 -0.268400 -1.761769	N -1.998864 -1.768797 -0.470896
	N 0.404615 1.797797 -0.070415	N -0.428749 1.552935 -0.856341	N 0.517093 -1.184633 1.337811
	N -2.081609 0.685435 -1.357425	N 2.059804 1.357176 0.774267	N -1.862610 0.679134 1.604102
	N 0.577092 -0.724803 -1.529206	N -0.537753 0.172340 1.704621	N 0.763102 1.822328 0.350485
	C 2.981004 -0.574710 0.572671	C -2.984166 -0.774625 -0.249662	C 2.993929 -0.215159 -0.879225
	H 0.145310 -0.806723 -2.449157	H -0.162565 0.711068 2.484228	H 0.472843 2.646974 0.873619
	H -1.689763 1.118262 -2.194163	H 1.659976 2.159548 1.260327	H -1.332332 0.987761 2.419440
	H 1.392582 1.590552 -0.252417	H -1.413824 1.439947 -0.574793	H 1.496876 -0.873698 1.255902
	H -1.947759 1.715878 1.592543	H 1.887727 0.556702 -2.321058	H -2.074451 -2.506607 0.228382
	H -2.523636 -1.858405 0.434505	H 2.535013 -1.819738 0.513783	H -2.724162 0.995954 -1.510237
	H -1.016502 -2.346548 0.835274	H 1.010209 -2.420709 0.492131	H -1.319814 0.808383 -2.335327
	H -1.528391 -2.433075 -0.713957	H 1.608546 -1.700046 1.840728	H -1.570841 2.146293 -1.435586
	H -2.644301 0.256784 1.758950	H 2.547949 -0.785989 -1.690616	H -2.951938 -1.607616 -0.793523

	H -1.175584 0.532668 2.414383	H 1.038477 -0.852601 -2.311988	H -1.500502 -2.184305 -1.259667
	H 0.111082 2.492965 -0.755464	H -0.153629 2.507785 -0.630982	H 0.296674 -1.242677 2.330746
	H 0.388779 2.260152 0.837704	H -0.436609 1.478538 -1.873318	H 0.502549 -2.136125 0.971384
	H -2.694136 -0.055795 -1.697173	H 2.696928 0.925435 1.443860	H -2.461612 1.464483 1.348527
	H -2.702692 1.387712 -0.956177	H 2.661530 1.750908 0.050821	H -2.488649 -0.057017 1.932382
	H 0.884821 -1.663629 -1.273863	H -0.754292 -0.757591 2.064828	H 1.024691 2.152044 -0.578435
	H 1.439231 -0.186729 -1.652759	H -1.451154 0.579611 1.470171	H 1.638392 1.502996 0.773585
	H 1.791574 -0.585669 0.964924	H -1.758151 -1.028854 -0.512306	H 1.870314 -0.421878 -1.121370
	H 3.107302 -1.573039 0.155093	H -3.218688 -1.472941 0.551916	H 3.223168 0.713966 -1.402587
	H 3.566504 -0.404178 1.471447	H -3.512678 -0.969399 -1.178161	H 3.544589 -1.060896 -1.284243
	O 3.080674 0.426465 -0.386600	O -3.008757 0.565564 0.156794	O 3.101537 -0.088394 0.513895
	H 3.869800 0.971535 -0.263853	H -3.871796 0.977277 0.008439	H 3.967687 -0.373431 0.834644
	S=0	S=1	S=2
$(H_3N)_5FeO^{2+}$	Fe -0.608532 0.004479 0.005777	Fe 0.591236 -0.000637 -0.003619	Fe -0.592030 0.043885 0.009791
	O 0.539157 -0.404687 -1.282704	O -0.515599 -1.025737 -0.874423	O 0.516878 -0.584758 1.164903
	N -1.691423 0.921534 -1.483818	N 1.600419 -1.690522 0.538552	N -1.771025 0.415159 1.683246
	N -1.522428 -1.798931 -0.370738	N 1.679544 0.033607 -1.724624	N 0.442662 2.028888 0.098514
	N 0.563255 -0.945403 1.377498	N -0.428934 1.629239 -0.630401	N 0.628838 -0.531868 -1.569361
	N -2.066626 0.493254 1.423426	N 1.999650 1.188023 0.999248	N -2.009458 0.881848 -1.265763
	N 0.419403 1.746530 0.274719	N -0.552389 -0.102096 1.653764	N -1.659070 -1.986383 -0.161909
	C 2.989668 -0.455443 -0.644215	C -2.973947 -0.750685 -0.311994	C 2.964046 -0.802342 0.487290
	H 0.099322 2.439055 0.950614	H -0.139727 0.210705 2.531790	H -1.603202 -2.487289 -1.047843
	H -1.662198 0.857025 2.286983	H 2.573299 0.661252 1.657866	H -2.639479 0.182991 -1.659331
	H 1.431526 -0.433253 1.562477	H -1.410090 1.497192 -0.353991	H 0.880111 -1.513098 -1.449362
	H -1.526859 -2.473130 0.393888	H 1.847336 0.947763 -2.144574	H 1.423067 1.776618 -0.047687
	H -1.075090 0.845878 -2.296703	H 1.155661 -2.421656 -0.020227	H -1.316375 -0.097362 2.441088
	H -1.887126 1.913058 -1.350042	H 1.504834 -1.977513 1.512434	H -2.743404 0.112479 1.646096
	H -2.578681 0.496090 -1.750057	H 2.599115 -1.724418 0.337212	H -1.787356 1.394263 1.968268
	H -2.476542 -1.779046 -0.728902	H 2.585945 -0.432367 -1.709871	H 0.236612 2.805400 -0.527409
	H -0.935994 -2.192376 -1.110470	H 1.092164 -0.489969 -2.377788	H 0.409369 2.406984 1.044236
	H 0.157251 -1.191681 2.279530	H -0.139252 2.549544 -0.301900	H 0.257135 -0.438083 -2.513339
	H 0.864766 -1.816493 0.939296	H -0.444897 1.670149 -1.648903	H 1.515911 -0.018726 -1.538528
	H -2.717590 1.211137 1.106517	H 2.662526 1.644434 0.372572	H -2.605959 1.569043 -0.805554
	H -2.645982 -0.296358 1.707287	H 1.580377 1.939864 1.546419	H -1.593307 1.367165 -2.060056
	H 0.474011 2.213536 -0.629777	H -0.828579 -1.074999 1.790122	H -2.637153 -2.049380 0.117366
	H 1.391822 1.507114 0.502797	H -1.434441 0.406617 1.536648	H -1.140482 -2.549486 0.512565
	H 1.811465 -0.453920 -1.024539	H -1.751875 -0.924288 -0.639413	H 1.767806 -0.784534 0.859667
	H 3.552926 -0.073965 -1.491235	H -3.145457 -1.533416 0.425738	H 3.543701 -0.832735 1.405002
	H 3.189900 -1.504566 -0.429037	H -3.531190 -0.887416 -1.233867	O 3.141906 0.339321 -0.279643
	O 3.038089 0.356035 0.492226	O -3.047386 0.535957 0.225131	H 3.039075 -1.716464 -0.101803
	H 3.883680 0.818416 0.572675	H -3.888351 0.969676 0.024354	H 3.908535 0.855258 0.003161
$(H_3N)_5FeO^{2+}$	S=3		
	Fe 0.574158 -0.005545 -0.018735		
	O -0.521174 -0.957971 -1.177060		
	N 1.888666 0.257809 -1.785253		
	N -0.520672 1.834444 -0.405742		
	N -0.666692 -0.400285 1.740578		
	N 2.024395 1.191117 1.226805		
	N 1.742049 -1.840156 0.439522		
	C -3.030017 -0.727820 -0.456340		
	H 1.685038 -2.176375 1.400037		
	H 2.719974 0.628687 1.715749		
	H -0.904313 -1.392282 1.751491		
	H -1.502677 1.610424 -0.201983		
	H 1.504557 -0.385429 -2.480068		
	H 2.880206 0.038523 -1.700024		
	H 1.845857 1.182298 -2.212774		
	H -0.297856 2.693572 0.093595		
	H -0.495291 2.058384 -1.399744		
	H -0.298177 -0.181981 2.665063		
	H -1.561938 0.090788 1.652887		
	H 2.555058 1.859691 0.668991		
	H 1.565424 1.745229 1.949382		
	H 2.734928 -1.832239 0.210063		
	H 1.320889 -2.567059 -0.140796		
	H -1.918663 -0.841029 -0.845267		
	H -3.649385 -0.753983 -1.349329		
	O -3.111824 0.473225 0.256178		
	H -3.179461 -1.601777 0.179178		
	H -3.962411 0.913129 0.124389		
	S=1/2	S=3/2	S=5/2
$(H_3N)_5CoO^{2+}$	Co -0.613650 -0.016740 0.005388	Co -0.587724 -0.029333 -0.024641	Co -0.568556 -0.003386 0.017583
	O 0.534314 1.276784 -0.528271	O 0.545894 0.563590 -1.168475	O 0.581488 -0.783388 1.215703
	N -1.548527 1.444691 0.965187	N -1.623446 1.995327 0.148133	N -1.644402 -1.898001 -0.228078
	N -1.605977 0.437308 -1.648305	N -1.684964 -0.429218 -1.637511	N -1.848326 0.382621 1.757468
	N 0.449346 -1.298444 -1.037399	N 0.376176 -2.061248 -0.097503	N 0.429244 1.898011 0.206376
	N -1.971110 -1.428015 0.576117	N -2.018074 -0.801253 1.241062	N -2.093137 0.973553 -1.273895
	N 0.496778 -0.289456 1.606672	N 0.590494 0.455042 1.502877	N 0.639502 -0.487368 -1.716592
	C 2.984157 0.728567 -0.203442	C 2.979823 0.827381 -0.413636	C 3.052276 -0.707773 0.454439
	H 0.743413 0.637055 1.956877	H 0.839395 1.441624 1.439048	H 0.905005 -1.471421 -1.701490

	H	-1.529568	-2.283511	0.913760	H	-2.768958	-1.300604	0.765674	H	-2.641050	1.666563	-0.765864
	H	1.395997	-1.375732	-0.641559	H	1.366649	-1.823378	-0.021717	H	1.410465	1.731626	-0.029524
	H	-1.722801	-0.315995	-2.325613	H	-1.894544	-1.417562	-1.772799	H	-1.787701	1.329875	2.128273
	H	-0.947772	2.247244	0.753345	H	-1.144648	2.527141	-0.578635	H	-1.228952	-2.583130	0.402574
	H	-1.597001	1.360783	1.980291	H	-1.499151	2.526422	1.008958	H	-1.605898	-2.308692	-1.159888
	H	-2.491970	1.681684	0.660505	H	-2.618007	2.062682	-0.063673	H	-2.633577	-1.857255	0.012436
	H	-2.526680	0.861653	-1.541995	H	-2.570038	0.073179	-1.690593	H	-2.843765	0.194416	1.650820
	H	-0.994642	1.140724	-2.074250	H	-1.118212	-0.128183	-2.433328	H	-1.512347	-0.228577	2.501924
	H	0.085343	-2.241584	-1.167782	H	0.198054	-2.800103	0.580620	H	0.108033	2.674239	-0.368646
	H	0.594765	-0.894223	-1.962725	H	0.275885	-2.492012	-1.014921	H	0.432946	2.230759	1.169310
	H	-2.585688	-1.122289	1.330390	H	-1.626237	-1.465194	1.908312	H	-1.685824	1.474033	-2.062601
	H	-2.593245	-1.721716	-0.176716	H	-2.472161	-0.073532	1.792702	H	-2.773432	0.333496	-1.681161
	H	1.398013	-0.723640	1.373882	H	1.474377	-0.063042	1.458860	H	1.519693	0.032349	-1.698889
	H	0.092411	-0.812308	2.382734	H	0.196491	0.310365	2.431294	H	0.222004	-0.319249	-2.630317
	H	1.770860	1.029200	-0.390393	H	1.815370	0.822225	-0.810908	H	1.891987	-0.754376	0.889270
	H	3.299236	1.405546	0.588085	H	3.048065	1.716386	0.214107	H	3.084738	-1.584835	-0.192188
	H	3.459298	0.919648	-1.161990	H	3.583040	0.895678	-1.314314	H	3.684861	-0.796822	1.333277
	O	2.989748	-0.622697	0.177570	O	3.144842	-0.342425	0.311729	O	3.194557	0.467086	-0.254994
	H	3.877572	-1.007232	0.148038	H	3.897069	-0.864256	0.003002	H	3.901634	1.023775	0.098673
	S=0				S=1				S=2			
$(H_3N)_5NiO^{2+}$	Ni	-0.616841	0.026215	0.039734	Ni	-0.612509	0.039567	0.031621	Ni	-0.608752	-0.007318	0.002870
	O	0.472467	-0.518633	-1.266242	O	0.482151	-0.676734	-1.184250	O	0.604098	0.894951	-1.180121
	N	-1.542995	1.113195	-1.401751	N	-1.504031	0.939856	-1.519823	N	-1.595409	1.894213	0.273321
	N	-1.826233	-1.682435	-0.532315	N	-1.857344	-1.735782	-0.345172	N	-1.821755	-0.314678	-1.762632
	N	0.449403	-1.150178	1.251662	N	0.417102	-0.986541	1.374264	N	0.412523	-1.847747	-0.338811
	N	-1.883943	0.581517	1.479389	N	-1.900752	0.768160	1.383291	N	-2.099196	-0.987992	1.214585
	N	0.669976	1.707612	0.418626	N	0.697455	1.749726	0.232347	N	0.608396	0.352321	1.729773
	C	2.969145	-0.133125	-0.824370	C	2.972374	-0.229605	-0.796117	C	3.128441	0.720099	-0.374182
	H	0.300065	2.514406	0.919002	H	0.331173	2.608152	0.641029	H	0.163648	0.136977	2.620408
	H	-2.501618	1.343526	1.201997	H	-2.520488	1.487194	1.011800	H	-2.655574	-0.345813	1.776946
	H	1.420408	-0.811826	1.259536	H	1.394928	-0.668443	1.340834	H	1.392774	-1.726191	-0.080970
	H	-1.934203	-2.422677	0.159729	H	-1.979071	-2.390269	0.426449	H	-1.920529	-1.287338	-2.048354
	H	-0.992046	0.886961	-2.232314	H	-1.469341	1.958389	-1.518146	H	-1.432394	2.340468	1.174251
	H	-1.511838	2.124898	-1.283076	H	-2.473084	0.677496	-1.693270	H	-2.607328	1.845053	0.168969
	H	-2.512486	0.872428	-1.602035	H	-0.947758	0.613591	-2.313117	H	-1.274109	2.558048	-0.430567
	H	-2.758830	-1.518872	-0.908373	H	-2.784569	-1.616533	-0.750168	H	-2.770269	0.048432	-1.686551
	H	-1.266351	-2.071588	-1.292374	H	-1.291080	-2.214634	-1.046856	H	-1.399685	0.170715	-2.553778
	H	0.143421	-1.268304	2.216082	H	0.106246	-0.975892	2.344357	H	0.070279	-2.653663	0.180586
	H	0.483020	-2.071685	0.818090	H	0.431941	-1.954948	1.057373	H	0.411071	-2.120703	-1.319790
	H	-2.490491	-0.184654	1.770355	H	-2.506095	0.038743	1.758882	H	-2.769646	-1.524365	0.666105
	H	-1.397639	0.900665	2.316904	H	-1.425189	1.189621	2.180797	H	-1.696628	-1.650172	1.876221
	H	0.969053	2.041035	-0.497030	H	1.006794	1.981927	-0.710627	H	0.888874	1.329910	1.788794
	H	1.528170	1.429021	0.899130	H	1.549069	1.516915	0.747314	H	1.476487	-0.184364	1.707260
	H	1.788693	-0.359386	-1.063305	H	1.788335	-0.476423	-1.021009	H	2.018312	0.796670	-0.822515
	H	3.141724	0.828828	-1.306886	H	3.152490	0.686477	-1.358287	H	3.136351	1.531473	0.354895
	H	3.517041	-0.947366	-1.290542	H	3.519630	-1.083312	-1.185909	H	3.791914	0.899858	-1.216644
	O	3.097715	-0.070039	0.561858	O	3.090416	-0.046993	0.580147	O	3.268994	-0.506990	0.230352
	H	3.905661	-0.499278	0.874568	H	3.884540	-0.466408	0.938345	H	3.934198	-1.057192	-0.206032

Table S9. Optimized geometries (Cartesian coordinates in Å) for the products of the reaction between methanol and $(NH_3)_5MO^{2+}$ species.

	S=1/2	S=3/2	
$(H_3N)_5VO^{2+}$	V -0.651399 -0.024458 0.038241	V 0.657243 -0.024739 -0.039539	
	O 0.454777 -0.477120 1.356900	O -0.419696 -0.481658 -1.384211	
	N -1.916163 0.745771 1.655863	N 1.934477 0.786699 -1.625545	
	N 0.323985 1.896008 -0.190572	N -0.356739 1.875307 0.188140	
	N 0.689664 -0.839168 -1.473200	N -0.691612 -0.884256 1.440113	
	N -2.114246 0.680963 -1.508180	N 2.073577 0.688533 1.543287	
	N -1.758461 -1.904935 0.078404	N 1.799678 -1.883544 -0.084805	
	C 3.505921 -0.610141 0.486813	C -3.533247 -0.588156 -0.510279	
	H -1.647297 -2.528468 -0.720686	H 1.677447 -2.524076 0.699096	
	H -1.686187 1.025299 -2.367276	H 2.728383 -0.029511 1.852784	
	H 0.933398 -1.803149 -1.245286	H -0.923985 -1.845406 1.189451	
	H 0.162086 2.465674 -1.019355	H -1.350384 1.623011 0.180865	
	H -2.113386 1.745288 1.685055	H 1.367528 0.583092 -2.451660	
	H -1.332310 0.542345 2.470043	H 2.841575 0.351443 -1.788102	
	H -2.812604 0.292393 1.827678	H 2.114046 1.789889 -1.639083	
	H 0.179774 2.508251 0.611608	H -0.201662 2.453803 1.012077	
	H 1.321096 1.659431 -0.178781	H -0.224932 2.483596 -0.619233	
	H 0.392405 -0.851165 -2.448124	H -0.402375 -0.914924 2.417051	
	H 1.576284 -0.326179 -1.447035	H -1.583080 -0.379046 1.418228	
	H -2.765769 -0.044238 -1.807653	H 2.655265 1.462627 1.222733	
	H -2.699293 1.443996 -1.167900	H 1.619851 1.019765 2.394345	
	H -2.762666 -1.845975 0.244578	H 2.806877 -1.804865 -0.222268	
	H -1.370991 -2.404783 0.881007	H 1.442407 -2.374538 -0.906715	
	H 1.392601 -0.739802 1.352438	H -1.351371 -0.754215 -1.407891	
	H 4.321135 -0.531128 1.185741	H -4.391125 -0.491826 -1.153233	
	O 3.195329 0.473813 -0.288720	O -3.202687 0.456363 0.311260	
	H 3.206652 -1.564585 0.082890	H -3.178798 -1.554840 -0.188178	
	H 3.877436 1.156156 -0.221013	H -3.899378 1.127149 0.316100	
	S=0	S=1	S=2
$(H_3N)_5CrO^{2+}$	Cr -0.674042 0.005626 -0.016848	Cr -0.679321 -0.007725 -0.003969	Cr 0.680368 0.007235 -0.005062
	O 0.236301 0.794369 -1.349956	O 0.126397 1.125790 -1.195251	O -0.106194 -1.183125 -1.154652
	N -1.845233 1.784801 0.041633	N -1.933687 1.656180 0.392423	N 1.942589 -1.630904 0.463791
	N -2.002466 -0.804922 -1.457569	N -1.989762 -0.560968 -1.579180	N 2.001787 0.509774 -1.587962
	N 0.539994 -1.709926 -0.175442	N 0.631404 -1.556819 -0.542079	N -0.635625 1.527708 -0.609931
	N -1.843355 -0.920865 1.559603	N -1.731260 -1.272385 1.408514	N 1.710186 1.332245 1.367131
	N 0.650745 0.883086 1.378286	N 0.634465 0.667963 1.496632	N -0.640251 -0.619256 1.510439
	C 3.480656 0.465837 -0.595947	C 3.476540 0.524855 -0.550244	C -3.491486 -0.542554 -0.531831
	H 0.350098 0.978471 2.347853	H 0.348492 0.634411 2.474446	H -0.359479 -0.554703 2.488199
	H -2.493774 -0.274185 2.006453	H -1.108814 -1.762548 2.051276	H 1.078523 1.836752 1.989502
	H 0.295331 -2.563620 0.324547	H 0.400394 -2.514052 -0.278462	H -0.412603 2.493461 -0.371564
	H -1.993366 -1.815140 -1.594749	H -1.862837 -1.490079 -1.979339	H 1.866476 1.418560 -2.029877
	H -1.465886 2.297798 -0.757725	H -1.557385 2.328793 -0.281709	H 1.575952 -2.330067 -0.188400
	H -1.731734 2.393579 0.851448	H -1.858297 2.090903 1.311468	H 1.861171 -2.031850 1.397543
	H -2.850920 1.712168 -0.107276	H -2.932696 1.575679 0.207101	H 2.942730 -1.551034 0.284456
	H -2.986150 -0.543350 -1.403487	H -2.994127 -0.457862 -1.438668	H 3.005594 0.428351 -1.430129
	H -1.639059 -0.394532 -2.321168	H -1.716987 0.105868 -2.305384	H 1.748982 -0.191817 -2.288103
	H 0.608114 -1.959544 -1.162460	H 0.736916 -1.550192 -1.557305	H -0.731195 1.493127 -1.625461
	H 1.496701 -1.463546 0.101793	H 1.571718 -1.364183 -0.175429	H -1.578478 1.341155 -0.246046
	H -2.417834 -1.700533 1.239502	H -2.380720 -0.756600 2.002556	H 2.361950 0.844429 1.981944
	H -1.273172 -1.297410 2.317212	H -2.292452 -2.000451 0.966318	H 2.265710 2.051228 0.903408
	H 0.854280 1.825437 1.043889	H 0.806579 1.648023 1.267148	H -0.810453 -1.606191 1.310645
	H 1.557311 0.404347 1.399684	H 1.554867 0.217434 1.443139	H -1.560775 -0.171261 1.437976
	H 1.194806 0.753592 -1.489065	H 1.019324 1.012417 -1.545446	H -0.981869 -1.079376 -1.546869
	H 3.080528 1.461160 -0.481018	H 2.963416 1.463987 -0.418657	H -2.946557 -1.462671 -0.394759
	H 4.321414 0.271421 -1.239873	H 4.377678 0.449617 -1.134177	H -4.415110 -0.506872 -1.083149
	O 3.250297 -0.377547 0.458912	O 3.256326 -0.397303 0.441388	O -3.257372 0.414232 0.423795
	H 3.995831 -0.975952 0.607235	H 4.029496 -0.960582 0.584578	H -4.032763 0.974644 0.565741
	S=1/2	S=3/2	S=5/2
$(H_3N)_5MnO^{2+}$	Mn -0.662802 0.009069 -0.021206	Mn -0.672094 0.000418 0.014466	Mn -0.660493 0.013175 0.016286
	O 0.225577 1.057534 -1.163699	O 0.163136 -0.699017 1.432516	O 0.253310 -0.156565 -1.489514
	N -1.847611 1.681196 0.335934	N -1.883400 -1.681826 0.089970	N -1.852603 1.484412 -1.045878
	N -1.959057 -0.548657 -1.555022	N -1.951377 0.972885 1.326416	N -2.126052 -1.597318 -0.766896
	N 0.572397 -1.582334 -0.467693	N 0.592147 1.623535 0.053450	N 0.560693 -1.489523 0.910709
	N -1.784318 -1.181200 1.360214	N -1.743521 0.769836 -1.638642	N -1.816384 0.221626 1.757740
	N 0.596404 0.609541 1.511750	N 0.615797 -1.018859 -1.236361	N 0.750808 1.656070 0.740372
	C 3.476471 0.534040 -0.535341	C 3.471295 -0.371887 0.654548	C 3.442119 0.000461 -0.867551
	H 0.274099 0.541039 2.476400	H 0.290372 -1.275094 -2.168078	H 0.500498 2.296522 1.492223
	H -1.197902 -1.662811 2.042077	H -1.137797 1.081913 -2.398280	H -1.251794 0.335053 2.599855
	H 1.531860 -1.365971 -0.172384	H 1.543319 1.357302 -0.225246	H 1.530040 -1.149745 0.909594
	H -1.875303 -1.500900 -1.909377	H -1.867051 1.986480 1.396901	H -2.114672 -2.515630 -0.325076
	H -2.840847 1.632041 0.112402	H -2.885920 -1.556685 0.225028	H -2.859637 1.341129 -1.108517
	H -1.432690 2.351859 -0.316186	H -1.526411 -2.138077 0.933571	H -1.481593 1.402434 -1.994355
	H -1.792866 2.103938 1.261943	H -1.786676 -2.360762 -0.664470	H -1.722689 2.457110 -0.769818
	H -2.956669 -0.392748 -1.414411	H -2.949899 0.781697 1.252197	H -3.112232 -1.371901 -0.888960

	H -1.669867 0.068838 -2.316520	H -1.645714 0.595726 2.226549	H -1.747134 -1.733746 -1.704939
	H 0.341726 -2.493619 -0.073165	H 0.337336 2.438951 -0.503141	H 0.355235 -1.811562 1.855226
	H 0.621111 -1.711525 -1.478250	H 0.672179 1.948442 1.017250	H 0.554094 -2.314442 0.311006
	H -2.450156 -0.637268 1.909202	H -2.375304 0.086079 -2.055570	H -2.435670 1.031658 1.726769
	H -2.336046 -1.912101 0.910931	H -2.326620 1.572943 -1.403352	H -2.417649 -0.586247 1.921075
	H 0.788282 1.596085 1.334329	H 0.817955 -1.891501 -0.746935	H 0.937502 2.237163 -0.076553
	H 1.511490 0.149241 1.469993	H 1.524356 -0.560239 -1.356766	H 1.656652 1.255137 0.995659
	H 1.101290 0.876926 -1.534200	H 1.090531 -0.510576 1.636318	H 1.204726 -0.361389 -1.497267
	H 2.971475 1.473717 -0.379740	H 2.998493 -1.338578 0.730161	H 2.999546 0.898355 -1.270156
	H 4.384111 0.467920 -1.110381	H 4.345510 -0.127824 1.233643	H 4.267765 -0.485633 -1.359165
	O 3.236201 -0.416034 0.424342	O 3.264276 0.280913 -0.532196	O 3.288728 -0.163369 0.481721
	H 3.999219 -0.997448 0.548113	H 4.029104 0.816509 -0.785132	H 4.045074 -0.623808 0.871566
	S=0	S=1	S=2
$(H_3N)_5FeO^{2+}$	Fe 0.665666 -0.005409 -0.000163	Fe -0.665302 0.004906 0.004820	Fe 0.660325 0.037076 -0.001311
	O -0.121598 1.253804 -1.007025	O 0.129769 -1.571030 0.323801	O -0.373711 -0.552765 -1.310568
	N 1.969042 -0.209462 -1.556589	N -1.882456 -1.079463 -1.219399	N 1.899532 0.561067 -1.564369
	N 1.834550 1.517682 0.690861	N -1.910858 -0.457684 1.558246	N -0.422277 2.016538 -0.079941
	N -0.629126 0.354381 1.516396	N 0.586472 0.913189 1.304150	N -0.664149 -0.655158 1.399206
	N 1.679438 -1.453920 1.094594	N -1.690098 1.763174 -0.418273	N 1.946824 0.798664 1.425230
	N -0.572192 -1.397130 -0.789311	N 0.614059 0.355623 -1.533433	N 1.772767 -1.979616 0.082797
	C -3.463614 0.630334 -0.419600	C 3.462329 -0.752255 0.104242	C -3.484891 -0.760751 -0.448386
	H -0.310881 -2.378427 -0.701167	H 0.294220 0.962305 -2.287790	H 1.606634 -2.596505 0.877007
	H 1.062758 -2.076850 1.616163	H -2.303001 1.681160 -1.229227	H 2.468506 0.060766 1.898617
	H -1.537368 -0.106061 1.394189	H 1.531418 0.982828 0.909591	H -0.867026 -1.636469 1.206532
	H 1.722076 1.790680 1.666915	H -1.828434 0.102611 2.406282	H -1.405444 1.754374 0.013750
	H 1.671870 0.555893 -2.167395	H -1.561376 -2.031875 -1.025555	H 1.385975 0.197388 -2.371075
	H 1.897649 -1.064996 -2.106694	H -1.773860 -0.934737 -2.222826	H 2.831718 0.149462 -1.578777
	H 2.962452 -0.082545 -1.367702	H -2.885693 -1.053821 -1.042051	H 2.027517 1.561512 -1.714314
	H 2.839865 1.460256 0.533951	H -2.909413 -0.523919 1.365376	H -0.261770 2.799109 0.551843
	H 1.481654 2.293922 0.124732	H -1.582750 -1.397931 1.792686	H -0.344975 2.389410 -1.025342
	H -0.315594 0.132117 2.461195	H 0.335545 1.843777 1.637179	H -0.388221 -0.601177 2.378798
	H -0.826066 1.355865 1.498632	H 0.681798 0.319470 2.128711	H -1.562225 -0.163904 1.308681
	H 2.252549 -2.060409 0.508229	H -2.294754 2.065580 0.345844	H 2.645937 1.434802 1.042860
	H 2.321801 -1.061968 1.783484	H -1.077523 2.555102 -0.613229	H 1.466084 1.321481 2.157010
	H -0.656669 -1.201099 -1.787412	H 0.798880 -0.558820 -1.948151	H 2.781798 -1.998876 -0.059313
	H -1.522512 -1.302156 -0.414720	H 1.529831 0.708186 -1.235985	H 1.364794 -2.449821 -0.726068
	H -1.086377 1.218302 -1.095625	H 1.078280 -1.568300 0.523775	H -1.300705 -0.784152 -1.115176
	H -4.290391 0.646285 -1.109205	H 3.021287 -1.405986 -0.631948	H -4.237617 -0.717126 -1.217030
	H -3.059996 1.542289 -0.007567	H 4.313259 -1.058498 0.688470	O -3.256826 0.349793 0.316846
	O -3.264225 -0.489816 0.344244	O 3.259675 0.585926 -0.113027	H -3.201956 -1.697888 0.005634
	H -4.017853 -1.095241 0.302424	H 4.020771 1.115795 0.162845	H -3.952694 1.011438 0.200082
$(H_3N)_5FeO^{2+}$	S=3		
	Fe 0.639303 0.034910 -0.100600		
	O 0.137118 0.530438 -1.724649		
	N 2.171647 1.629376 0.004304		
	N -0.802412 1.328513 0.906780		
	N -0.719798 -1.674897 -0.065170		
	N 1.241681 -0.588171 1.984866		
	N 2.244122 -1.297726 -0.854154		
	C -3.523363 0.359034 -0.687302		
	H 2.065206 -2.295587 -0.749013		
	H 1.876444 -1.384943 2.020039		
	H -0.830799 -1.976543 -1.033203		
	H -1.666245 0.822153 1.126605		
	H 2.510295 1.744860 -0.951014		
	H 2.994079 1.518691 0.595219		
	H 1.763533 2.530308 0.251764		
	H -0.523519 1.820440 1.753876		
	H -1.082507 2.037192 0.227702		
	H -0.475075 -2.503415 0.474314		
	H -1.657871 -1.391203 0.242690		
	H 1.698220 0.150918 2.518131		
	H 0.427817 -0.859338 2.535985		
	H 3.187344 -1.144526 -0.500304		
	H 2.281361 -1.125429 -1.859067		
	H -0.145819 0.812811 -2.601198		
	H -4.525703 0.580407 -1.006646		
	O -3.323275 -0.291146 0.503703		
	H -2.684293 0.318629 -1.362082		
	H -4.151602 -0.411265 0.986813		
	S=1/2	S=3/2	S=5/2
$(H_3N)_5CoO^{2+}$	Co -0.670326 0.023713 -0.000454	Co 0.645246 0.013915 -0.018021	Co 0.620913 -0.039319 -0.068094
	O 0.102824 -1.554562 0.504653	O -3.231992 0.412807 0.270301	O -0.464239 -0.703812 -1.256367
	N -1.841587 -1.167738 -1.060604	O -0.470741 -0.547235 -1.252502	N 1.786689 -1.860377 0.090078
	N -1.852225 -0.252374 1.560566	N -0.648757 -0.448449 1.393793	N 2.030827 0.669913 -1.558679
	N 0.580029 1.008688 1.153015	N 2.028532 0.704501 1.335155	N -0.448101 1.837928 -0.081661
	N -1.620609 1.711806 -0.589997	N -0.224857 2.093454 -0.100995	N 1.939122 0.875606 1.452089
	N 0.573771 0.067903 -1.524036	N 1.800760 0.422690 -1.578900	N -0.640867 -0.702496 1.565042
	C 3.465368 -0.682350 0.226646	N 1.623871 -2.019938 0.133492	C -3.577622 -0.592901 -0.515291
	H 0.764426 -0.918204 -1.712626	C -3.510846 -0.791151 -0.310652	H -0.815243 -1.699102 1.444151

	H -0.975478 2.439488 -0.898108	H -3.822836 1.108546 -0.047490	H 2.488015 1.648359 1.077117
	H 1.515924 1.076624 0.735449	H -4.236160 -0.829843 -1.105810	H -1.430097 1.555871 -0.006765
	H -1.753908 0.400602 2.337283	H -3.316694 -1.651921 0.310234	H 1.993344 1.669810 -1.749228
	H -1.461322 -2.081286 -0.790974	H -1.375701 -0.815374 -1.001528	H 1.376139 -2.475537 -0.613030
	H -1.771522 -1.098248 -2.075330	H -0.373777 -0.224582 2.349286	H 1.718306 -2.353138 0.979017
	H -2.838514 -1.170139 -0.848411	H -1.537804 0.033911 1.204484	H 2.780491 -1.802624 -0.125397
	H -2.853738 -0.335336 1.390025	H 2.306252 -0.016968 2.000396	H 3.015119 0.445532 -1.423551
	H -1.509213 -1.164007 1.880813	H 1.669475 1.483664 1.886588	H 1.741250 0.205730 -2.419753
	H 0.304726 1.950565 1.430227	H 0.363282 2.901414 -0.299491	H -0.275082 2.539199 0.635610
	H 0.706676 0.468799 2.010368	H -0.819035 2.369687 0.679190	H -0.358423 2.308677 -0.980110
	H -2.259426 1.563673 -1.371061	H 1.227015 0.093495 -2.360030	H 1.421940 1.263762 2.239806
	H -2.185393 2.137031 0.145101	H 2.693226 -0.067234 -1.621093	H 2.613726 0.228580 1.858775
	H 1.485467 0.487843 -1.312341	H 2.629977 -2.095595 -0.009649	H -1.551508 -0.247683 1.454255
	H 0.244204 0.493483 -2.389993	H 1.428164 -2.593019 0.953078	H -0.354407 -0.574084 2.533697
	H 1.034436 -1.498347 0.761473	H 1.187201 -2.499080 -0.654328	H -1.333019 -0.783557 -1.679523
	H 3.018794 -1.469350 -0.360422	H 1.995378 1.410506 -1.736296	H -3.065721 -1.520809 -0.316330
	H 4.336411 -0.858442 0.834342	H -0.847406 1.980917 -0.900947	H -4.546341 -0.571367 -0.983775
	O 3.227853 0.589101 -0.230556	H 2.892735 1.039250 0.910653	O -3.216609 0.448732 0.299041
	H 3.982966 1.174780 -0.078803	H -0.854834 -1.446960 1.373018	H -3.944313 1.078026 0.395822
	S=0	S=1	S=2
$(H_3N)_5NiO^{2+}$	Ni -0.662166 0.031016 0.036688	Ni -0.662519 0.030903 0.036851	
	O 0.187366 -0.483683 -1.460585	O 0.192526 -0.480159 -1.458697	
	N -1.770052 1.134780 -1.217049	N -1.765473 1.139557 -1.217046	
	N -1.997607 -1.659198 -0.368913	N -1.997286 -1.656792 -0.379197	
	N 0.531883 -1.181732 1.049411	N 0.528382 -1.185849 1.048906	
	N -1.685572 0.580246 1.646870	N -1.691477 0.574895 1.645736	
	N 0.714661 1.685240 0.226306	N 0.715309 1.683493 0.237057	
	C 3.428993 -0.219736 -0.767490	C 3.429723 -0.211894 -0.768919	
	H 0.418126 2.535527 0.703240	H 0.418843 2.530770 0.719381	
	H -2.320298 1.358817 1.473269	H -2.327397 1.352425 1.471886	
	H 1.489511 -0.809807 1.072610	H 1.486131 -0.814698 1.075186	
	H -1.965292 -2.463700 0.255525	H -1.969188 -2.462900 0.243364	
	H -1.355527 0.874741 -2.115613	H -1.681583 2.150155 -1.116589	
	H -1.687107 2.145775 -1.119828	H -2.760970 0.926941 -1.259205	
	H -2.765401 0.920728 -1.255398	H -1.348420 0.881635 -2.115042	
	H -2.986398 -1.494492 -0.550684	H -2.984913 -1.490281 -0.565523	
	H -1.574111 -1.952499 -1.250774	H -1.569791 -1.948394 -1.259700	
	H 0.259791 -1.405573 2.005501	H 0.254237 -1.413569 2.003482	
	H 0.577292 -2.057565 0.528565	H 0.574227 -2.059288 0.524090	
	H -2.260459 -0.181888 2.005304	H -2.265672 -0.188986 2.001522	
	H -1.076948 0.875306 2.410088	H -1.085337 0.869850 2.410961	
	H 0.921668 1.943069 -0.738210	H 0.923019 1.947844 -0.725538	
	H 1.609866 1.415456 0.640432	H 1.610241 1.410834 0.649879	
	H 1.146581 -0.591221 -1.333048	H 1.152536 -0.580476 -1.330018	
	H 3.060822 0.570140 -1.403787	H 3.065558 0.586129 -1.397234	
	H 4.203933 -0.889463 -1.100526	H 4.201130 -0.882522 -1.108288	
	O 3.275628 -0.002972 0.574300	O 3.275234 -0.009405 0.574771	
	H 4.013505 -0.365495 1.084918	H 4.011284 -0.379999 1.082276	

Table S10. Numerical E_a values (kcal/mol) for the data used to make Figure 6 of the manuscript and Figure S3 of SI.

Species	E_a (CH_4)	E_a (CH_3OH)-FOS	E_a (CH_3OH)-POS
Ti/LS	51.2		
Ti/HS	2.3		
V/LS	43.5	43.8	23.0
V/HS	5.3	6.8	-13.1
Cr/LS	34.0	34.2	13.4
Cr/IS	16.2	16.8	-4.8
Cr/HS	6.1	7.1	-13.8
Mn/LS	15.5	15.9	-5.0
Mn/IS	25.6	26.8	4.4
Mn/HS	5.2	6.4	-13.4
Fe/LS	15.5	16.4	-5.7
Fe/IS	20.9	22.0	0.0
Fe/HS	19.0	20.4	-0.3
Fe/S=3	6.5	7.4	-12.6
Co/LS	11.5	12.7	-9.2
Co/IS	21.0	14.6	-5.9
Co/HS	12.6	12.6	-7.6
Ni/LS	9.3	10.4	-10.5
Ni/IS	10.0	10.9	-10.0
Ni/HS	9.5	8.9	-10.8

Table S11. Spin density on the oxygen atoms at the transition states (TS) of the $(NH_3)_5MO^{2+}$ + CH_4 and $(NH_3)_5MO^{2+}$ + CH_3OH reactions.

Species	TS/CH_4	TS/CH_3OH
Ti/LS	0.33	
Ti/HS	0.76	
V/LS	0.40	0.40
V/HS	0.66	0.67
Cr/LS	0.43	0.42
Cr/IS	0.37	0.50
Cr/HS	0.63	0.65
Mn/LS	0.44	0.47
Mn/IS	0.52	0.52
Mn/HS	0.67	0.72
Fe/LS	0.54	0.56
Fe/IS	0.59	0.60
Fe/HS	0.59	0.63
Fe/S=3	1.13	1.18
Co/LS	0.58	0.63
Co/IS	0.64	0.83
Co/HS	1.23	1.32
Ni/LS	0.68	0.72
Ni/IS	0.65	0.74
Ni/HS	1.46	1.58

Table S12. Optimized geometries (Cartesian coordinates in Å) for the singlet, triplet, quintet, and septet N_5FeO^{2+} species, their encounter complex (EC) with methane and the transition state (TS) for the C-H activation of methane.

Species	N_5FeO^{2+}	EC	TS
S=0	Fe 0.047593 -0.000034 -0.650732	N 1.552600 1.359646 -0.369069	N 1.470865 -1.351336 0.293684
	O 0.159149 -0.000039 -2.258501	N 1.552683 -1.360201 -0.369051	N 1.470938 1.351254 0.293396
	N 1.419374 1.360641 -0.311811	N -1.239834 1.353640 -0.238647	N -1.341992 -1.360169 0.225046
	N 1.419362 -1.360726 -0.311807	N -1.240266 -1.353817 -0.238700	N -1.342174 1.359949 0.225527
	N -1.379414 1.348494 -0.482448	N 0.188317 -0.000339 1.504819	N 0.047744 0.000115 -1.538616
	N -1.379497 -1.348477 -0.482466	C 1.818541 1.752461 0.894781	C 1.717120 -1.732685 -0.977526
	N -0.133120 -0.000042 1.407493	C -1.831205 1.209713 0.966761	C -1.958602 -1.214821 -0.967783
	C 1.551734 1.752632 0.973161	C 1.818666 -1.752912 0.894839	C 1.716860 1.733052 -0.977750
	C -2.083929 1.211481 0.662175	C -1.831654 -1.209670 0.966679	C -1.958810 1.214580 -0.967269
	C 1.551715 -1.752716 0.973168	C -1.627165 -2.324087 -1.077692	C -1.670331 2.375255 1.037546
	C -2.084012 -1.211424 0.662152	C -1.626069 2.324348 -1.077434	C -1.670265 -2.375393 1.037098
	C -1.676702 -2.321478 -1.355041	C 2.861974 2.631252 1.170825	C 2.774908 -2.582560 -1.285110
	C -1.676552 2.321531 -1.355006	C -3.263455 3.069076 0.515859	C -3.321139 -3.130747 -0.536973
	C 2.560342 2.631073 1.358004	C 2.862511 -2.631158 1.171004	C 2.774436 2.583216 -1.285256
	C -3.445790 3.087345 0.079919	C 0.874528 1.238160 1.957001	C 0.728914 -1.237556 -2.008623
	C 2.560358 -2.631104 1.358031	C 0.874137 -1.239114 1.956829	C 0.728549 1.237920 -2.008743
	C 0.501516 1.238192 1.930006	C 2.290775 -1.822920 -1.395302	C 2.245847 1.796072 1.298146
	C 0.501434 -1.238332 1.929972	C 2.290321 1.822775 -1.395417	C 2.245513 -1.796575 1.298456
	C 2.260998 -1.824775 -1.254610	C 3.336948 2.715197 -1.191398	C 3.309949 -2.661909 1.064693
	C 2.260965 1.824738 -1.254633	C -1.295579 -0.000111 1.714302	C -1.443837 0.000055 -1.720313
	C 3.280352 2.716870 -0.941108	C 3.630358 -3.120571 0.112460	C 3.580173 3.056437 -0.246869
	C -1.630618 0.000006 1.460165	C -3.265053 -3.068075 0.515433	C -3.321303 3.130562 -0.536406
	C 3.434748 -3.121657 0.386290	C 3.629432 3.121075 0.112203	C 3.580423 -3.056134 -0.246715
	C -3.446013 -3.087174 0.079857	C -2.647887 -3.207510 -0.727589	C -2.668760 3.283125 0.687513
	C 3.434685 3.121672 0.386247	C 3.337830 -2.714826 -1.191163	C 3.310177 2.661550 1.064442
	C -2.716663 -3.215576 -1.102122	C -2.646197 3.208376 -0.727134	C -2.668697 -3.283255 0.687010
	C 3.280423 -2.716859 -0.941065	C -2.850714 2.051435 1.386485	C -2.958341 -2.080271 -1.389389
	C -2.716441 3.215706 -1.102065	C -2.851697 -2.050829 1.386234	C -2.958558 2.080069 -1.388807
	C -3.127658 2.066017 0.985041	H -1.110831 -2.360337 -2.039511	H -1.114957 2.435013 1.976164
	C -3.127811 -2.065883 0.984996	H -1.109740 2.360419 -2.039265	H -1.115014 -2.435125 1.975796
	H -1.070177 -2.352739 -2.262642	H 3.065695 2.931209 2.199942	H 2.961426 -2.873400 -2.320065
	H -1.070033 2.352758 -2.262614	H -4.064125 3.748905 0.812107	H -4.103892 -3.830472 -0.834776
	H 2.654846 2.930099 2.403104	H 3.066258 -2.931031 2.200139	H 2.960660 2.874502 -2.320138
	H -4.261946 3.777391 0.300719	H 1.403745 1.067156 2.905997	H 1.217787 -1.067462 -2.979036
	H 2.654854 -2.930129 2.403132	H 0.113918 2.011106 2.151814	H -0.032807 -2.017527 -2.166640
	H 0.927834 1.066728 2.929379	H 1.402779 -1.068690 2.906249	H 1.217190 1.068005 -2.979305
	H -0.275297 2.011059 2.044292	H 0.113309 -2.012077 2.150706	H -0.033350 2.017774 -2.166515
	H 0.927662 -1.066965 2.929400	H 2.009881 -1.452997 -2.383671	H 1.979084 1.431194 2.292751
	H -0.275413 -2.011186 2.044112	H 2.009475 1.452731 -2.383755	H 1.978511 -1.432056 2.293127
	H 2.085687 -1.455737 -2.267352	H 3.913023 3.077106 -2.042856	H 3.917828 -3.010366 1.899473
	H 2.085649 1.455695 -2.267372	H -1.589367 -0.000090 2.775261	H -1.756640 0.000403 -2.775835
	H 3.942607 3.079314 -1.727170	H 4.452153 -3.812155 0.305399	H 4.414286 3.726214 -0.462468
	H -2.034116 0.000010 2.484451	H -4.066150 -3.747458 0.811552	H -4.104034 3.830312 -0.834208
	H 4.231501 -3.813376 0.664794	H 4.450890 3.813084 0.305047	H 4.414708 -3.725675 -0.462378
	H -4.262225 -3.777159 0.300640	H -2.952735 -3.985822 -1.427082	H -2.924988 4.095498 1.367472
	H 4.231409 3.813431 0.664736	H 3.914193 -3.076428 -2.042557	H 3.918322 3.009639 1.899181
	H -2.947454 -3.995352 -1.827828	H -2.950543 3.987040 -1.426456	H -2.925003 -4.095590 1.366986
	H 3.942712 -3.079267 -1.727115	H -3.312789 1.920176 2.365830	H -3.438445 -1.945038 -2.359493
	H -2.947179 3.995510 -1.827759	H -3.313722 -1.919431 2.365585	H -3.438700 1.944850 -2.358896
	H -3.680400 1.941302 1.917138	H -4.453970 -0.890942 -2.353722	H -2.185250 0.912204 3.926881
	H -3.680552 -1.941138 1.917090	C -3.907359 0.000343 -2.019336	C -2.170166 0.000063 3.320547
		H -4.452283 0.894434 -2.348962	H -2.185538 -0.913145 3.925269
		H -3.843430 -0.002721 -0.918688	H -2.893415 0.000842 2.495875
		H -2.898458 0.000523 -2.459530	H -0.985398 0.000354 2.776739
		O 0.081472 -0.000315 -2.170892	O 0.112098 -0.000218 2.240358
		Fe 0.152485 -0.000318 -0.560126	Fe 0.059955 0.000069 0.488323
S=1	Fe 0.047253 -0.000028 -0.646156	N 1.552009 1.358645 -0.369566	N -1.471970 1.348087 0.294228
	O 0.158595 -0.000029 -2.250691	N 1.552144 -1.359288 -0.369547	N -1.471798 -1.348404 0.294259
	N 1.419005 1.359788 -0.312356	N -1.238726 1.353184 -0.239127	N 1.342150 1.361000 0.224935
	N 1.418995 -1.359857 -0.312353	N -1.239167 -1.353506 -0.239198	N 1.342401 -1.360904 0.224982
	N -1.377942 1.348224 -0.482658	N 0.187559 -0.000441 1.502632	N -0.045868 -0.000079 -1.535507
	N -1.378008 -1.348209 -0.482673	C 1.817721 1.752734 0.894076	C -1.717596 1.730310 -0.976853
	N -0.133215 -0.000034 1.405407	C -1.832002 1.209606 0.965499	C 1.960371 1.214783 -0.966976
	C 1.551055 1.753311 0.972321	C 1.817975 -1.753224 0.894141	C -1.717562 -1.730451 -0.976856
	C -2.084300 1.211407 0.660955	C -1.832475 -1.209726 0.965393	C 1.960577 -1.214611 -0.966946
	C 1.551039 -1.753379 0.972327	C -1.624871 -2.324325 -1.077970	C 1.671103 -2.376878 1.036000
	C -2.084368 -1.211360 0.660936	C -1.623661 2.324563 -1.077598	C 1.670745 2.376979 1.035992
	C -1.674228 -2.321704 -1.354898	C 2.860834 2.631864 1.170237	C -2.776643 2.578295 -1.285512
	C -1.674107 2.321746 -1.354870	C -3.263071 3.069537 0.513634	C 3.323650 3.130347 -0.536858
	C 2.558973 2.632585 1.357064	C 2.861674 -2.631596 1.170426	C -2.776781 -2.578201 -1.285548
	C -3.445436 3.087370 0.077707	C 0.873479 1.238504 1.955849	C -0.727095 1.237415 -2.006525
	C 2.558987 -2.632609 1.357086	C 0.873068 -1.239682 1.955638	C -0.726908 -1.237704 -2.006448
	C 0.500945 1.238682 1.928867	C 2.291269 -1.821158 -1.395138	C -2.249292 -1.791313 1.297804

	C	0.500879	-1.238795	1.928840	C	2.290669	1.821071	-1.395260	C	-2.249482	1.790983	1.297770
	C	2.261248	-1.823459	-1.254524	C	3.337018	2.714013	-1.191500	C	-3.315630	2.654018	1.063309
	C	2.261222	1.823428	-1.254542	C	-1.296968	-0.000205	1.713035	C	1.445360	0.000034	-1.719246
	C	3.279977	2.716569	-0.941485	C	3.630136	-3.120340	0.112050	C	-3.585025	-3.049058	-0.248105
	C	-1.631411	0.000005	1.458884	C	-3.264873	-3.068520	0.513021	C	3.324084	-3.130025	-0.536872
	C	3.433595	-3.122897	0.385454	C	3.628809	3.121165	0.111783	C	-3.584892	3.049145	-0.248076
	C	-3.445618	-3.087231	0.077656	C	-2.645931	-3.207863	-0.729120	C	2.670496	-3.283857	0.686289
	C	3.433543	3.122910	0.385419	C	3.338213	-2.713393	-1.191257	C	-3.315577	-2.654177	1.063313
	C	-2.714536	-3.215759	-1.103228	C	-2.644024	3.208790	-0.728466	C	2.670037	3.284078	0.686303
	C	3.280033	-2.716560	-0.941451	C	-2.851624	2.051638	1.384445	C	2.960921	2.079315	-1.388557
	C	-2.714356	3.215865	-1.103182	C	-2.852719	-2.051128	1.384096	C	2.961214	-2.079029	-1.388555
	C	-3.128356	2.065932	0.982948	H	-1.107336	-2.361598	-2.039069	H	1.115084	-2.439025	1.974044
	C	-3.128481	-2.065822	0.982911	H	-1.106140	2.361679	-2.038714	H	1.114728	2.439023	1.974044
	H	-1.066575	-2.354047	-2.261672	H	3.063948	2.932420	2.199297	H	-2.962073	2.869630	-2.320519
	H	-1.066459	2.354061	-2.261649	H	-4.063850	3.749603	0.808999	H	4.107007	3.829436	-0.834538
	H	2.652803	2.932382	2.402001	H	3.064871	-2.932030	2.199505	H	-2.962335	-2.869375	-2.320578
	H	-4.261813	3.777437	0.297566	H	1.401899	1.066601	2.905077	H	-1.213528	1.067364	-2.978164
	H	2.652812	-2.932405	2.402024	H	0.112265	2.010845	2.150490	H	0.034337	2.018110	-2.162179
	H	0.926715	1.066330	2.928275	H	1.400755	-1.068474	2.905397	H	-1.213110	-1.067921	-2.978248
	H	-0.276520	2.010841	2.043164	H	0.111633	-2.012097	2.149101	H	0.034622	-2.018380	-2.161712
	H	0.926576	-1.066522	2.928292	H	2.011906	-1.450511	-2.383625	H	-1.983654	-1.427402	2.292952
	H	-0.276613	-2.010944	2.043020	H	2.011422	1.450244	-2.383713	H	-1.983943	1.426949	2.292898
	H	2.087726	-1.453407	-2.267157	H	3.913435	3.075220	-2.043020	H	-3.925480	3.000386	1.897521
	H	2.087697	1.453371	-2.267172	H	-1.589133	-0.000186	2.774393	H	1.756656	0.000052	-2.775215
	H	3.942379	3.078482	-1.727660	H	4.451770	-3.812123	0.304877	H	-4.420407	-3.717011	-0.464408
	H	-2.033324	0.000008	2.483749	H	-4.066149	-3.748083	0.808193	H	4.107519	-3.829019	-0.834572
	H	4.229788	-3.815354	0.663671	H	4.449974	3.813533	0.304511	H	-4.420137	3.717277	-0.464355
	H	-4.262040	-3.777249	0.297501	H	-2.949500	-3.986431	-1.428879	H	2.926499	-4.096628	1.365861
	H	4.229712	3.815400	0.663624	H	3.914979	-3.074195	-2.042712	H	-3.925401	-3.000579	1.897529
	H	-2.944131	-3.995807	-1.829017	H	-2.947003	3.987807	-1.427984	H	2.925951	4.096859	1.365896
	H	3.942463	-3.078445	-1.727615	H	-3.314695	1.920348	2.363315	H	3.441672	1.942948	-2.358185
	H	-2.943908	3.995935	-1.828961	H	-3.315742	-1.919725	2.362973	H	3.441921	-1.942605	-2.358197
	H	-3.682071	1.941065	1.914446	H	-4.454434	-0.890610	-2.353838	H	2.171418	-0.914099	3.933666
	H	-3.682195	-1.940931	1.914406	C	-3.907911	0.000590	-2.019100	C	2.170649	0.000165	3.330904
					H	-4.452740	0.894817	-2.348498	H	2.170822	0.914108	3.934155
					H	-3.843990	-0.002832	-0.918490	H	2.897725	0.000616	2.510041
					H	-2.898924	0.000916	-2.459024	H	0.970749	-0.000017	2.762942
					O	0.083001	-0.000355	-2.163284	O	-0.104628	0.000019	2.232277
					Fe	0.152139	-0.000382	-0.555621	Fe	-0.058245	-0.000081	0.487397
S=2	Fe	0.065056	-0.000269	-0.781376	N	1.644770	-1.435303	0.416156	N	1.704699	-1.375960	0.249075
	O	0.203868	-0.000354	-2.372389	N	1.644477	1.435198	0.416210	N	1.409688	1.456086	0.415514
	N	1.502000	1.439533	-0.364341	N	-1.340000	-1.415899	0.270337	N	-1.323864	-1.461542	0.317362
	N	1.503057	-1.439336	-0.363842	N	-1.340208	1.415704	0.270198	N	-1.597680	1.373434	0.171666
	N	-1.491957	1.405623	-0.509865	N	0.186812	-0.000195	-1.383506	N	0.048084	-0.003509	-1.431423
	N	-1.491446	-1.405986	-0.510389	C	1.887160	-1.740424	-0.875881	C	1.930866	-1.583799	-1.060856
	N	-0.114033	-0.000289	1.289683	C	-1.877063	-1.214485	-0.951028	C	-1.896385	-1.344781	-0.904571
	C	1.623038	1.743881	0.944909	C	1.886836	1.740399	-0.875821	C	1.623810	1.829582	-0.866965
	C	-2.126848	1.216266	0.666328	C	-1.877133	1.214145	-0.951199	C	-2.107786	1.084903	-1.039308
	C	1.624045	-1.743445	0.945460	C	-1.809115	2.393862	1.058122	C	-2.127427	2.368376	0.895429
	C	-2.126461	-1.217187	0.665852	C	-1.808778	-2.393993	1.058418	C	-1.647224	-2.493546	1.113007
	C	-1.882276	-2.387620	-1.335815	C	2.967313	-2.537320	-1.242303	C	3.092119	-2.198090	-1.519477
	C	-1.883064	2.387506	-1.334848	C	-3.406144	-3.021667	-0.620233	C	-3.197598	-3.317884	-0.522240
	C	2.662377	2.543469	1.410207	C	2.966667	2.537782	-1.242143	C	2.635308	2.726373	-1.194260
	C	-3.594197	3.048796	0.214135	C	0.866544	-1.236563	-1.868425	C	0.792308	-1.180848	-1.968264
	C	2.663946	-2.542070	1.411141	C	0.866587	1.236112	-1.868531	C	0.653622	1.282252	-1.888027
	C	0.516274	1.236054	1.837847	C	2.447855	1.910346	1.385642	C	2.168778	1.955690	1.407479
	C	0.516524	-1.236458	1.837922	C	2.448486	-1.910025	1.385507	C	2.614761	-1.769413	1.155258
	C	2.392157	-1.916897	-1.254231	C	3.536329	-2.727124	1.096292	C	3.794100	-2.408786	0.780527
	C	2.390455	1.917769	-1.255024	C	-1.289605	-0.000208	-1.653092	C	-1.429805	-0.117844	-1.674648
	C	3.444266	2.737743	-0.865749	C	3.799231	3.040655	-0.238702	C	3.424642	3.253428	-0.168296
	C	-1.609865	-0.000523	1.418279	C	-3.406256	3.021370	-0.620806	C	-3.732120	2.823462	-0.832162
	C	3.585098	-3.047325	0.489673	C	3.800217	-3.039779	-0.238932	C	4.035228	-2.621137	-0.578026
	C	-3.593363	-3.049877	0.212797	C	-2.849808	3.224010	0.641907	C	-3.205907	3.119726	0.425292
	C	3.582853	3.049418	0.488451	C	3.535344	2.727933	1.096509	C	3.187174	2.867833	1.152484
	C	-2.939563	-3.235384	-1.005105	C	-2.849538	-3.224175	0.642433	C	-2.588382	-3.445381	0.726199
	C	3.446585	-2.735919	-0.864593	C	-2.913705	-1.996889	-1.439613	C	-2.843158	-2.252459	-1.359129
	C	-2.940521	3.234916	-1.003738	C	-2.913725	1.996490	-1.440003	C	-3.174732	1.783909	-1.588279
	C	-3.183981	2.018713	1.071402	H	-1.337978	2.493194	2.038836	H	-1.668877	2.555221	1.870090
	C	-3.183448	-2.020035	1.070499	H	-1.337556	-2.493187	2.039103	H	-1.128519	-2.541755	2.072343
	H	-1.332412	-2.477754	-2.275367	H	3.151085	-2.767800	-2.292853	H	3.255474	-2.350762	-2.587500
	H	-1.333300	2.478171	-2.274409	H	-4.221217	-3.656948	-0.971279	H	-3.937868	-4.049024	-0.851875
	H	2.747655	2.773272	2.473440	H	3.150445	2.768316	-2.292680	H	2.799913	3.013896	-2.233781
	H	-4.422185	3.699822	0.500080	H	1.322814	-1.061708	-2.853994	H	1.142236	-0.974357	-2.990693
	H	2.749142	-2.771683	2.474422	H	0.102844	-2.019177	-2.002247	H	0.088901	-2.026345	-2.031326
	H	0.879940	1.059588	2.860919	H	1.323225	1.061020	-2.853888	H	1.142077	1.151543	-2.864945
	H	-0.257717	2.017266	1.902834	H	0.102871	2.018622	-2.002864	H	-0.153686	2.018106	-2.027941
	H	0.879439	-1.060115	2.861283	H	2.191623	1.612911	2.405286	H	1.926874	1.595478	2.410294
	H	-0.257141	-2.018056	1.902150	H	2.192241	-1.612680	2.405176	H	2.373297	-1.553071	2.199332
	H	2.232194	-1.620023	-2.293449	H	4.165227	-3.103422	1.902989	H	4.509819	-2.725103	1.539283
	H	2.230470	1.620625	-2.294161	H	-1.518057	-0.000291	-2.730562	H	-1.671007	-0.200959	-2.746258

	H 4.143822 3.116790 -1.610595<br>H -1.938523 -0.000785 2.469521<br>H 4.407614 -3.679166 0.829391<br>H -4.421226 -3.701198 0.498429<br>H 4.404907 3.682009 0.827891<br>H -3.240602 -4.023555 -1.695184<br>H 4.146660 -3.114447 -1.609216<br>H -3.241779 4.023292 -1.693489<br>H -3.678657 1.848934 2.028887<br>H -3.678251 -1.850735 2.028003	H 4.650645 3.671534 -0.499903<br>H -4.221277 3.656625 -0.972020<br>H 4.651893 -3.670276 -0.500201<br>H -3.215370 4.010336 1.302317<br>H 4.163978 3.104559 1.903259<br>H -3.215031 -4.010411 1.302987<br>H -3.330064 -1.816192 -2.431740<br>H -3.329960 1.815669 -2.432160<br>H -4.602403 0.893048 2.292554<br>C -4.046631 0.000022 1.978667<br>H -4.602758 -0.892819 2.292460<br>H -3.944316 0.000063 0.881074<br>H -3.053447 -0.000256 2.453339<br>O 0.137862 0.000166 2.291857<br>Fe 0.166165 -0.000056 0.694449	H 4.222455 3.960664 -0.401761<br>H -4.571632 3.397820 -1.227688<br>H 4.954249 -3.110040 -0.905796<br>H -3.620053 3.922013 1.036061<br>H 3.784028 3.260851 1.975275<br>H -2.833395 -4.270292 1.395046<br>H -3.289728 -2.138273 -2.347921<br>H -3.562886 1.531023 -2.576140<br>H -2.050031 0.574507 4.226159<br>C -2.050880 -0.306093 3.575452<br>H -1.966031 -1.251481 4.121396<br>H -2.825771 -0.294350 2.799709<br>H -0.906266 -0.172463 2.935557<br>O 0.117339 0.085046 2.345873<br>Fe 0.063591 -0.009832 0.615039
S=3	Fe 0.059624 0.000001 -0.883036<br>O 0.320732 0.000012 -2.749245<br>N 1.494334 1.451880 -0.339988<br>N 1.494328 -1.451886 -0.339990<br>N -1.500811 1.415862 -0.515721<br>N -1.500812 -1.415860 -0.515722<br>N -0.131449 0.000000 1.345769<br>C 1.603784 1.743451 0.971891<br>C -2.126484 1.219569 0.664412<br>C 1.603781 -1.743454 0.971890<br>C -2.126484 -1.219567 0.664411<br>C -1.883806 -2.422188 -1.319008<br>C -1.883804 2.422189 -1.319008<br>C 2.639828 2.541194 1.450544<br>C -3.573849 3.081212 0.250984<br>C 2.639826 -2.541195 1.450542<br>C 0.499517 1.231298 1.871696<br>C 0.499516 -1.231299 1.871696<br>C 2.391182 -1.944007 -1.218200<br>C 2.391190 1.944000 -1.218198<br>C 3.442731 2.758664 -0.814561<br>C -1.619442 0.000001 1.421608<br>C 3.568870 -3.056986 0.544133<br>C -3.573849 -3.081211 0.250986<br>C 3.568874 3.056983 0.544136<br>C -2.926001 -3.279163 -0.968229<br>C 3.442724 -2.758670 -0.814564<br>C -2.926001 3.279163 -0.968230<br>C -3.168457 2.033188 1.087745<br>C -3.168456 -2.033186 1.087746<br>H -1.338368 -2.527836 -2.260196<br>H -1.338366 2.527836 -2.260195<br>H 2.714758 2.760818 2.516864<br>H -4.390601 3.738506 0.554481<br>H 2.714758 -2.760817 2.516862<br>H 0.880085 1.070736 2.892352<br>H -0.269148 2.017761 1.943176<br>H 0.880085 -1.070737 2.892352<br>H -0.269150 -2.017760 1.943177<br>H 2.241025 -1.668338 -2.265925<br>H 2.241035 1.668330 -2.265923<br>H 4.147440 3.145112 -1.550628<br>H -1.995593 0.000000 2.457848<br>H 4.387317 -3.686619 0.897579<br>H -4.390601 -3.738505 0.554484<br>H 4.387319 3.686617 0.897582<br>H -3.218655 -4.083988 -1.642473<br>H 4.147432 -3.145119 -1.550632<br>H -3.218654 4.083988 -1.642474<br>H -3.654236 1.857503 2.048808<br>H -3.654235 -1.857501 2.048809	N 1.579037 -1.444393 0.365933<br>N 1.578901 1.444564 0.365836<br>N -1.407438 -1.432166 0.225565<br>N -1.407836 1.432109 0.225574<br>N 0.125804 0.000172 -1.473587<br>C 1.821661 -1.733035 -0.928489<br>C -1.925009 -1.221452 -1.002537<br>C 1.821375 1.733572 -0.928527<br>C -1.925473 1.221066 -1.002490<br>C -1.855433 2.453053 0.975532<br>C -1.854722 -2.453217 0.975614<br>C 2.908022 -2.518986 -1.303580<br>C -3.399307 -3.089648 -0.745717<br>C 2.907648 2.519734 -1.303503<br>C 0.807701 -1.229907 -1.932857<br>C 0.807336 1.230489 -1.932833<br>C 2.390439 1.928659 1.327331<br>C 2.390508 -1.928726 1.327330<br>C 3.485430 -2.731959 1.028720<br>C -1.345859 -0.000071 -1.701832<br>C 3.748316 3.027593 -0.310457<br>C -3.400549 3.088674 -0.745610<br>C 3.748630 -3.027094 -0.310612<br>C -2.857943 3.307531 0.520900<br>C 3.485293 2.732023 1.028834<br>C -2.856808 -3.308136 0.520921<br>C -2.925430 -2.028661 -1.527446<br>C -2.926317 2.027809 -1.527309<br>H -1.397513 2.568772 1.961371<br>H -1.396877 -2.568625 1.961521<br>H 3.089822 -2.735340 -2.357629<br>H -4.184910 -3.743106 -1.129226<br>H 3.089319 2.736438 -2.357504<br>H 1.284337 -1.071012 -2.912650<br>H 0.052483 -2.019771 -2.074916<br>H 1.283770 1.071865 -2.912775<br>H 0.051920 2.020202 -2.074560<br>H 2.135193 1.655576 2.355031<br>H 2.135191 -1.655923 2.355090<br>H 4.117912 -3.111898 1.830841<br>H -1.614045 -0.000114 -2.771182<br>H 4.604130 3.648224 -0.581681<br>H -4.186519 3.741734 -1.129047<br>H 4.604522 -3.647573 -0.581939<br>H -3.203414 4.125909 1.152447<br>H 4.117876 3.111743 1.830980<br>H -3.202066 -4.126578 1.152507<br>H -3.324519 -1.839433 -2.525136<br>H -3.325484 1.838301 -2.524916<br>H -3.406845 0.893752 3.442370<br>C -3.053121 -0.000277 2.912913<br>H -3.406077 -0.894923 3.441871<br>H -3.436713 -0.000215 1.881746<br>H -1.947287 0.000166 2.913569<br>O 0.266623 0.000213 2.645005<br>Fe 0.079815 0.000151 0.770875	

Table S13. Optimized geometries (Cartesian coordinates in Å) for the singlet, triplet, quintet, and septet N_5FeO^{2+} species, their encounter complex (EC) with methane and the transition state (TS) for the C-H activation of methane.

Species	EC	TS
S=1	N 1.649872 1.359898 -0.387467	N -1.577091 1.338228 0.353396
	N 1.650629 -1.360277 -0.387523	N -1.552702 -1.359607 0.334575
	N -1.134243 1.350908 -0.146028	N 1.206603 1.384718 0.077644
	N -1.134711 -1.351610 -0.146314	N 1.221984 -1.363614 0.048219
	N 0.364025 -0.000764 1.536498	N -0.274598 0.015468 -1.587686
	C 1.966649 1.753528 0.864302	C -1.917190 1.726233 -0.893970
	C -1.672819 1.210873 1.082863	C 1.754532 1.247248 -1.146042
	C 1.967281 -1.754165 0.864217	C -1.881744 -1.739197 -0.918288
	C -1.673417 -1.211425 1.082515	C 1.782201 -1.179151 -1.164662
	C -1.551921 -2.326239 -0.964946	C 1.596307 -2.397490 0.813415
	C -1.550550 2.326277 -0.964238	C 1.568225 2.402001 0.872162
	C 3.019725 2.633226 1.098315	C -3.003636 2.566285 -1.118683
	C -3.098737 3.089182 0.706599	C 3.128025 3.170121 -0.787500
	C 3.021124 -2.632907 1.098292	C -2.949199 -2.600104 -1.154824
	C 1.067219 1.237523 1.963027	C -1.001250 1.248011 -1.998048
	C 1.066742 -1.239452 1.962610	C -0.974347 -1.227550 -2.014653
	C 2.347821 -1.822390 -1.441854	C -2.250677 -1.816464 1.388708
	C 2.346151 1.823187 -1.441900	C -2.283770 1.766883 1.413641
	C 3.399687 2.716277 -1.280528	C -3.372145 2.620946 1.263622
	C -1.112184 -0.000521 1.806157	C 1.201969 0.033419 -1.871637
	C 3.746248 -3.121736 0.010021	C -3.675487 -3.085666 -0.064949
	C -3.100963 -3.088345 0.705468	C 3.205647 -3.069760 -0.837081
	C 3.743909 3.123250 0.009971	C -3.738905 3.022218 -0.022085
	C -2.545898 -3.218955 -0.567557	C 2.598380 -3.274124 0.402066
	C 3.402172 -2.714523 -1.280431	C -3.319649 -2.692406 1.226346
	C -2.543649 3.219772 -0.566426	C 2.537593 3.319573 0.469919
	C -2.656484 2.066609 1.554415	C 2.727104 2.118632 -1.619685
	C -2.657895 -2.066437 1.553669	C 2.784040 -2.008664 -1.647742
	H -1.075050 -2.364190 -1.946968	H 1.075665 -2.497945 1.768265
	H -1.073752 2.364111 -1.946304	H 1.063266 2.453545 1.840117
	H 3.264102 2.933273 2.118504	H -3.266570 2.862566 -2.135354
	H -3.871742 3.781997 1.043459	H 3.887520 3.876707 -1.126710
	H 3.265372 -2.933160 2.118449	H -3.204169 -2.889859 -2.175387
	H 1.634421 1.064623 2.889435	H -1.558534 1.078928 -2.931127
	H 0.314031 2.009104 2.189110	H -0.261985 2.037671 -2.206756
	H 1.632871 -1.067579 2.889864	H -1.533891 -1.058121 -2.946309
	H 0.313270 -2.011269 2.186909	H -0.217618 -1.998158 -2.231301
	H 2.028716 -1.451517 -2.418124	H -1.918869 -1.452664 2.364059
	H 2.026997 1.452412 -2.418193	H -1.942884 1.398441 2.384116
	H 3.941188 3.077811 -2.154498	H -3.923726 2.955671 2.141969
	H -1.360017 -0.000616 2.878636	H 1.441595 0.044565 -2.946088
	H 4.574899 -3.813598 0.169608	H -4.515188 -3.763961 -0.224976
	H -3.874613 -3.780591 1.042019	H 3.994195 -3.740796 -1.181564
	H 4.571943 3.815858 0.169517	H -4.593826 3.683458 -0.172637
	H -2.872446 -4.003464 -1.250122	H 2.894588 -4.100911 1.047379
	H 3.944390 -3.075140 -2.154336	H -3.864733 -3.050396 2.099552
	H -2.869530 4.004853 -1.248652	H 2.817562 4.139839 1.131299
	H -3.068363 1.940536 2.556486	H 3.154363 1.987875 -2.614750
	H -3.069798 -1.940302 2.555722	H 3.219524 -1.839816 -2.633495
	H -3.988875 -0.895768 -2.757108	H 2.409944 -1.131534 3.394944
	C -3.569444 0.000902 -2.272859	C 2.421828 -0.131562 2.940041
	H -3.989288 0.897590 -2.756715	H 2.554661 0.663836 3.685931
	O -3.792981 0.000538 -0.866244	O 3.300300 -0.079416 1.869995
	H -2.479448 0.001178 -2.428602	H 1.205463 -0.017230 2.544215
	O 0.116822 -0.000543 -2.123713	O -0.020184 0.005310 2.161154
	Fe 0.242362 -0.000560 -0.518524	Fe -0.143972 -0.001004 0.432575
	H -4.745017 0.000312 -0.701204	H 3.789523 0.754311 1.865022
S=2	N 1.741534 1.433745 -0.430766	N 1.781095 -1.388693 0.323035
	N 1.744663 -1.433180 -0.429482	N 1.511491 1.445266 0.440978
	N -1.234536 1.414927 -0.176669	N -1.240899 -1.458869 0.141269
	N -1.233363 -1.415349 -0.178419	N -1.461517 1.394936 -0.040093
	N 0.350588 -0.000680 1.419690	N 0.277434 -0.018475 -1.496462
	C 2.030661 1.739624 0.851211	C 2.111683 -1.606990 -0.962917
	C -1.724281 1.215859 1.063355	C -1.717280 -1.339622 -1.117856
	C 2.033377 -1.738534 0.852676	C 1.825601 1.804177 -0.824194
	C -1.723436 -1.217894 1.061811	C -1.892270 1.088991 -1.277071
	C -1.722901 -2.400205 -0.944763	C -2.038963 2.398890 0.631027
	C -1.724640 2.400542 -0.941602	C -1.638449 -2.478155 0.918222
	C 3.123533 2.536985 1.177388	C 3.304217 -2.228306 -1.321362
	C -3.235284 3.047666 0.806986	C -3.072671 -3.291151 -0.825880
	C 3.127624 -2.533579 1.179861	C 2.871203 2.684697 -1.082566
	C 1.047332 1.235171 1.880143	C 1.053319 -1.204469 -1.962931
	C 1.047799 -1.236126 1.880425	C 0.927970 1.259139 -1.911647

	C	2.514070	-1.905687	-1.427023	C	2.201983	1.942671	1.482440
	C	2.509081	1.907899	-1.428991	C	2.613390	-1.777969	1.302394
	C	3.606772	2.725168	-1.180446	C	3.817354	-2.423024	1.029182
	C	-1.117124	-0.001236	1.741125	C	-1.177030	-0.121511	-1.853979
	C	3.925227	-3.033633	0.147218	C	3.589153	3.210228	-0.004854
	C	-3.233482	-3.050159	0.802812	C	-3.527955	2.827240	-1.202249
	C	3.919177	3.038759	0.144086	C	4.166834	-2.646471	-0.303772
	C	-2.732525	-3.243780	-0.484512	C	-3.083814	3.142871	0.081281
	C	3.613296	-2.720648	-1.177562	C	3.249292	2.838993	1.297621
	C	-2.734619	3.243093	-0.480169	C	-2.560097	-3.420465	0.466915
	C	-2.723163	2.014301	1.600718	C	-2.643058	-2.235654	-1.638056
	C	-2.722045	-2.017502	1.597907	C	-2.921624	1.777466	-1.904563
	H	-1.287524	-2.498247	-1.942220	H	-1.648846	2.599686	1.631997
	H	-1.289533	2.500174	-1.939024	H	-1.192209	-2.522468	1.914960
	H	3.345397	2.767998	2.220430	H	3.552849	-2.389301	-2.371565
	H	-4.020365	3.697989	1.196789	H	-3.794588	-4.015506	-1.207280
	H	3.349054	-2.764161	2.223090	H	3.116938	2.960590	-2.109167
	H	1.539728	1.059740	2.848084	H	1.487113	-1.009141	-2.955049
	H	0.288786	2.017431	2.041944	H	0.351664	-2.046084	-2.077060
	H	1.538145	-1.061043	2.849472	H	1.486966	1.118686	-2.848698
	H	0.289733	-2.019309	2.040011	H	0.140466	2.001595	-2.114810
	H	2.220803	-1.608045	-2.436585	H	1.881878	1.593425	2.467142
	H	2.215527	1.609630	-2.438281	H	2.288092	-1.552704	2.321543
	H	4.205836	3.100863	-2.009790	H	4.468622	-2.735121	1.845539
	H	-1.305729	-0.002001	2.826188	H	-1.336059	-0.207011	-2.940733
	H	4.786996	-3.662477	0.377514	H	4.411775	3.904821	-0.183750
	H	-4.018303	-3.701356	1.191683	H	-4.340562	3.394005	-1.659882
	H	4.779813	3.669414	0.373672	H	5.107875	-3.140000	-0.552616
	H	-3.111996	-4.038863	-1.126329	H	-3.537103	3.952456	0.653096
	H	4.213869	-3.095063	-2.006394	H	3.789275	3.231041	2.159233
	H	-3.114566	4.038784	-1.120951	H	-2.862263	-4.242962	1.115519
	H	-3.094638	1.838028	2.611194	H	-3.013772	-2.120389	-2.657578
	H	-3.093848	-1.842624	2.608501	H	-3.243570	1.509312	-2.912010
	H	-4.069946	-0.897308	-2.753042	H	-2.237636	0.942252	3.742255
	C	-3.653409	-0.000357	-2.266710	C	-2.313266	-0.044001	3.263292
	H	-4.071692	0.896291	-2.752078	H	-2.400105	-0.862857	3.990453
	O	-3.879941	-0.001357	-0.861592	O	-3.267675	-0.041267	2.264568
	H	-2.563011	0.000790	-2.421364	H	-1.150870	-0.129641	2.760791
	O	0.167038	-0.000961	-2.251488	O	0.040375	0.049968	2.262154
	Fe	0.250557	-0.000642	-0.654842	Fe	0.127222	-0.007570	0.548929
	H	-4.832238	-0.001993	-0.698115	H	-3.761675	-0.872087	2.249792
